# Repeated Administration of D-Amphetamine Induces Distinct Alterations in Behavior and Metabolite Levels in 129Sv and Bl6 Mouse Strains

**DOI:** 10.3389/fnins.2018.00399

**Published:** 2018-06-12

**Authors:** Taavi Vanaveski, Jane Narvik, Jürgen Innos, Mari-Anne Philips, Aigar Ottas, Mario Plaas, Liina Haring, Mihkel Zilmer, Eero Vasar

**Affiliations:** ^1^Department of Physiology, Institute of Biomedicine and Translational Medicine, University of Tartu, Tartu, Estonia; ^2^Center of Excellence for Genomics and Translational Medicine, University of Tartu, Tartu, Estonia; ^3^Department of Biochemistry, Institute of Biomedicine and Translational Medicine, University of Tartu, Tartu, Estonia; ^4^Psychiatry Clinic and Center of Excellence for Genomics and Translational Medicine, Institute of Biomedicine and Translational Medicine, University of Tartu, Tartu, Estonia; ^5^Psychiatry Clinic, Tartu University Hospital, Tartu, Estonia

**Keywords:** amphetamine, repeated administration, locomotor activity, behavioral sensitization, 129Sv strain, Bl6 strain, metabolomics, branched chain amino acids

## Abstract

The main goal of the study was to characterize the behavioral and metabolomic profiles of repeated administration (for 11 days) of d-amphetamine (AMPH, 3 mg/kg i. p.), indirect agonist of dopamine (DA), in widely used 129S6/SvEvTac (129Sv) and C57BL/6NTac (Bl6) mouse strains. Acute administration of AMPH (acute AMPH) induced significantly stronger motor stimulation in Bl6. However, repeated administration of AMPH (repeated AMPH) caused stronger motor sensitization in 129Sv compared acute AMPH. Body weight of 129Sv was reduced after repeated saline and AMPH, whereas no change occurred in Bl6. In the metabolomic study, acute AMPH induced an elevation of isoleucine and leucine, branched chain amino acids (BCAA), whereas the level of hexoses was reduced in Bl6. Both BCAAs and hexoses remained on level of acute AMPH after repeated AMPH in Bl6. Three biogenic amines [asymmetric dimethylarginine (ADMA), alpha-aminoadipic acid (alpha-AAA), kynurenine] were significantly reduced after repeated AMPH. Acute AMPH caused in 129Sv a significant reduction of valine, lysophosphatidylcholines (lysoPC a C16:0, lysoPC a C18:2, lysoPC a C20:4), phosphatidylcholine (PC) diacyls (PC aa C34:2, PC aa C36:2, PC aa C36:3, PC aa C36:4) and alkyl-acyls (PC ae C38:4, PC ae C40:4). However, repeated AMPH increased the levels of valine and isoleucine, long-chain acylcarnitines (C14, C14:1-OH, C16, C18:1), PC diacyls (PC aa C38:4, PC aa C38:6, PC aa C42:6), PC acyl-alkyls (PC ae C38:4, PC ae C40:4, PC ae C40:5, PC ae C40:6, PC ae C42:1, PC ae C42:3) and sphingolipids [SM(OH)C22:1, SM C24:0] compared to acute AMPH in 129Sv. Hexoses and kynurenine were reduced after repeated AMPH compared to saline in 129Sv. The established changes probably reflect a shift in energy metabolism toward lipid molecules in 129Sv because of reduced level of hexoses. Pooled data from both strains showed that the elevation of isoleucine and leucine was a prominent biomarker of AMPH-induced behavioral sensitization. Simultaneously a significant decline of hexoses, citrulline, ADMA, and kynurenine occurred. The reduced levels of kynurenine, ADMA, and citrulline likely reflect altered function of N-methyl-D-aspartate (NMDA) and NO systems caused by repeated AMPH. Altogether, 129Sv strain displays stronger sensitization toward AMPH and larger variance in metabolite levels than Bl6.

## Introduction

Bl6 and 129Sv are widely used inbred mouse lines in biomedical research. The method of creating transgenic mice often involves these two strains. 129Sv derived embryonic stem cells are used for introducing targeted mutations into mouse genome (Linder and Davisson, 2004) and the Bl6 strain is employed as a background line in transgenic studies (Yoshiki and Moriwaki, 2006). Behavioral studies demonstrate that Bl6 are more active and venturous, while 129Sv tend to be idle and display higher level of anxiety (Contet et al., 2001; Võikar et al., 2001; Abramov et al., 2008; Heinla et al., 2014). Therefore, these marked behavioral differences between the mouse lines can be used to study the genetic susceptibility of animals to pharmacological treatments. Chen and colleagues performed a profound analysis by comparing AMPH effects on locomotor activity and DA efflux in Bl6 and 129Sv (Chen et al., 2007). They did not find differences in basal motor activity and DA levels between these strains. However, Bl6 showed greater AMPH-stimulated locomotor activity and stronger AMPH-induced striatal DA efflux than 129Sv (Chen et al., 2007). A similar difference was established when Bl6 and DBA/2 strains were compared. Zocchi and colleagues found that Bl6 demonstrated a greater, dose-dependent locomotor stimulant response to an acute injection of AMPH than DBA/2, which corresponded to larger increases in DA levels in the nucleus accumbens after AMPH treatment (Zocchi et al., 1998).

Comparing 129Sv and Bl6 strains, one has to keep in mind that the 129Sv and all related 129Sv strains carry a 25 base pair frameshift deletion within exon 6 of the *Disc1* gene resulting in a premature termination codon at exon 7 (Chubb et al., 2008). Koike and colleagues discovered the deletion while modifying the 129Sv *Disc1* allele to imitate the production of the hypothetical C-terminally truncated protein product (Koike et al., 2006). Moreover, they reported a significant difference in delayed non-match to place test, a specific test of working memory, for both 129Sv *Disc1* heterozygotes and homozygotes, compared to Bl6 mice. Recent evidence suggests a prominent role of DISC1 in the genetics of major psychiatric disorders like schizophrenia, bipolar disorder, and major depressive disorder (Niwa et al., 2016). Studies in rats demonstrate that misassembly of full-length DISC1 protein compromises DA homeostasis, leading to apparent behavioral deficits (Trossbach et al., 2016).

Although AMPH has considerable affinities for DA, noradrenaline and serotonin transporters, the DA transporter is associated with the stimulating and rewarding properties of AMPH (Koob and Nestler, 1997; Heal et al., 2013; Sitte and Freissmuth, 2015). AMPH exerts its actions through an increase in DA extracellular levels in the terminal and cell body regions of midbrain DA neurons, by causing reverse transport of DA and preventing its uptake via the DA transporter (Seiden et al., 1993; Sulzer et al., 1995). Repeated administration of AMPH has been used to model psychotic-like behavior in rodents (Ham et al., 2017). The majority of studies evaluating the development of AMPH-induced motor sensitization have been performed in rats. Repeated AMPH administration to adult rats produced robust sensitization toward AMPH, disrupted latent inhibition, and decreased attentional vigilance; this effect lasted for 90 days after the last injection (Murphy et al., 2001; Russig, 2002; Russig et al., 2003; Ham et al., 2017). Even though deficits in the attention set-shifting task were observed, spatial memory was not impaired in the Morris water maze, indicating that cognitive impairments in the model appear to be restricted to some prefrontal cortex dependent tasks (Stefani and Moghaddam, 2002; Featherstone et al., 2008).

**Figure 1:**
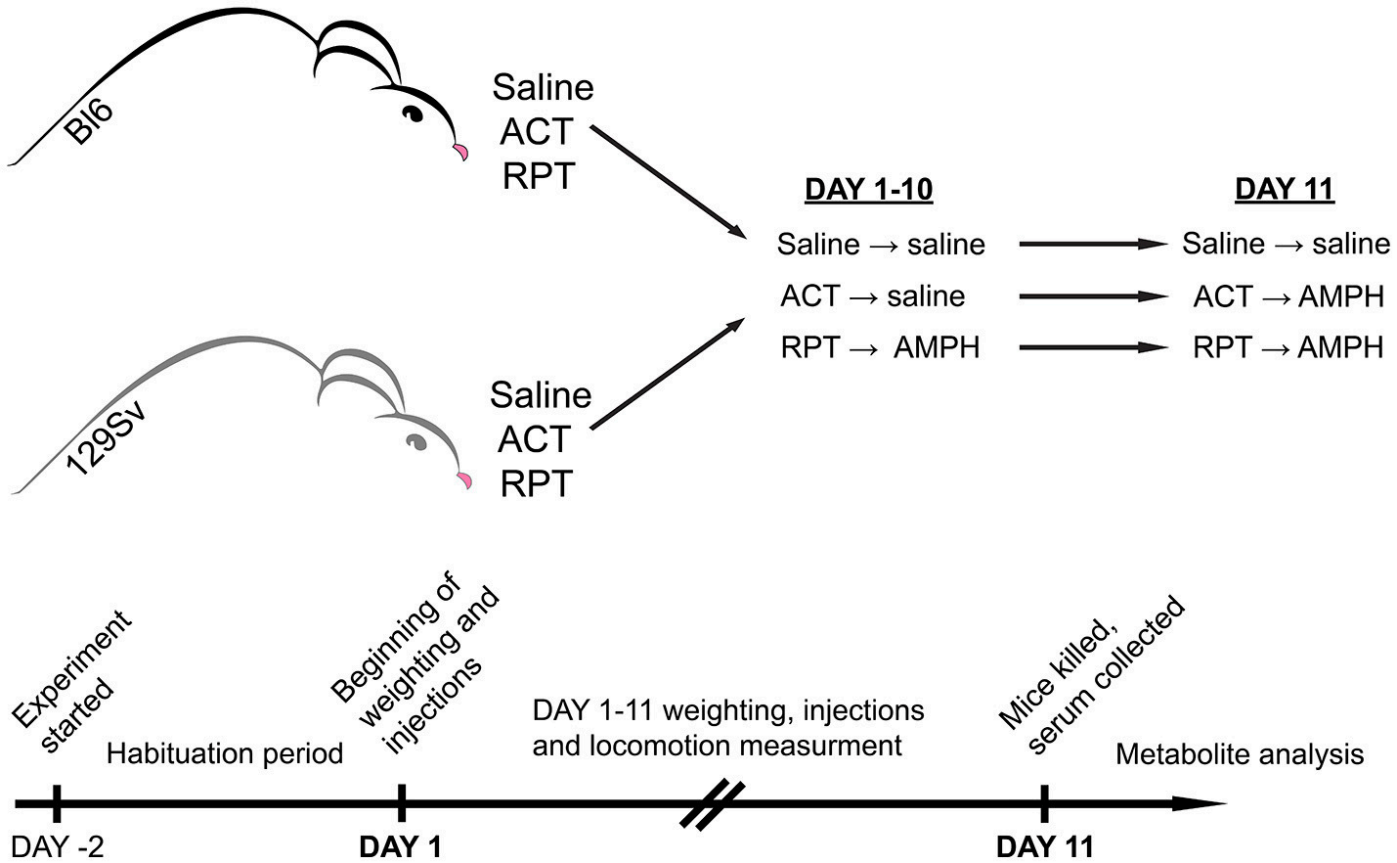
Representation of experimental design. Both mouse strains were studied for a period of 13 days. The first 2 days were allocated for adaptation to the testing environment, followed by experimental days 1–11. On experimental day 11 mice were sacrificed, blood was collected, and metabolites were stored for analysis. In both strains three groups were formed: saline (Saline), acute AMPH (ACT), and repeated AMPH (RPT).

So far few studies have been performed to examine mouse strain differences in behavioral sensitization to AMPH (Phillips et al., 2008). In comparison to Bl6, DBA/2 mice were more receptive to the development of motor sensitization (Badiani et al., 1992; Phillips et al., 1994). Despite extensive biomedical comparisons of 129Sv and Bl6 strains, we could not find any comprehensive studies comparing the effects of repeated AMPH in these two strains. Therefore, we hypothesize that these two mouse strains respond differently to repeated AMPH in terms of behavior and metabolomics. Based on above described data of DBA/2 mice we expect that 129Sv mice display stronger sensitization toward AMPH-induced hyper-locomotion compared to Bl6 strain. Previously, we have found the significant differences in the blood metabolite levels in Bl6 and 129Sv mice (Narvik et al., 2018). Thus, we expected to see significant differences in metabolite levels between these two mouse strains in response to repeated AMPH as well, especially in the levels of hexoses and lipid metabolites that are necessary to fuel the strong locomotor response. First, we aimed to study the effect of repeated AMPH on the locomotor activity of 129Sv and Bl6 strains. Simultaneously, changes in body weight were evaluated during repeated administrations. To study the effect of AMPH, both 129Sv and Bl6 mice were divided into three groups (Figure 1). The control group received saline injections for 11 days, the acute group received 10 days of saline, followed by AMPH administration on day 11, and the repeated AMPH group received 11 days of AMPH. Each day, after injections, locomotor activity was measured in all three administration groups (saline, acute AMPH and repeated AMPH) in both strains. The second aim was to identify possible metabolic consequences of distinct behavioral responses of Bl6 and 129Sv strains in all three administration groups. Blood serum was extracted after the last locomotor activity test and metabolite levels were determined with the Absolute*IDQ*™ p180 kit, using a combination of flow injection analysis and liquid chromatography tandem mass spectrometry technique. This method allowed the quantification of 160 metabolites, such as acylcarnitines, amino acids, biogenic amines, etc. Metabolic data of both lines were analyzed separately and together to establish the strain-specific effects and the effects of repeated AMPH independently from strain background. We would like to stress that such kind of comparative study has not been performed yet in these widely used mouse lines.

## Materials and methods

### Animals

Inbred male mice (C57BL/6NTac; Taconic Germantown, New York; *n* = 41 and 129S6/SvEvTac; Taconic Germantown, New York; *n* = 39) were used for the study. These animals were bred in the local animal facility, weaned from the mother at the age of 3 weeks, and thereafter divided into home cages with up to 10 pups. The animals were housed under a 12 h light/dark cycle with lights on at 7:00 a.m. Room temperature was kept at 21°C. Animals were housed in their respective home cages (1290D Eurostandard type III cages; 425 × 276 × 153 mm; Tecniplast, Italy) with bedding and nesting material. The bedding (aspen chips) and nesting material (aspen wool) were changed weekly. The animals had *ad libitum* access to Ssniff universal mouse and rat maintenance diet (cat# V1534; Ssniff, Germany) and reverse osmosis-purified water, except during testing. Behavioral testing, including habituation, started at the age of 6–9 weeks, and lasted for 13 days. At the time of sample collection, animals were on average 8–11 weeks old.

### Behavioral testing

Mouse strains were studied for a period of 13 days. The animals were divided randomly into three different administration groups. The first 2 days were allocated for adaptation to the testing environment, followed by experimental days 1–11 for locomotor activity measurements (Figure 1). On days 1–10 the following routine was used: animals were weighed, two groups of mice received an i.p. injection of 0.9% saline in the volume of 10 ml/kg, whereas the third group received AMPH (d-amphetamine, 3 mg/kg i.p., Sigma-Aldrich). We chose this dose of AMPH by comparing the results from our previous study (Innos et al., 2013) and performing a preliminary study with AMPH. Innos et al. (2013) achieved a dose-dependent motor activation with AMPH doses of 2.5, 5, and 7.5 mg/kg. We aimed to find a dose of AMPH that does not cause a robust elevation of locomotor activation in both strains. However, we thought that the 2.5 mg/kg of AMPH is too weak to achieve proper motor stimulation, because Innos et al. (2013) had used F2-hybrids having mixed background of Bl6 and 129Sv mice. To validate the dose we conducted a preliminary experiment with 3 mg/kg of AMPH and achieved a reasonable elevation of locomotor activity in both strains. In Bl6 mice the increase was 2-fold, whereas in 129Sv this change was almost 5-fold. Based on our sensitization curve, depicted in Supplementary Figure S1A, it is apparent that the chosen dose of AMPH is valid. AMPH was dissolved in 0.9% saline (3 mg of amphetamine in 10 ml). After administration of saline or AMPH, the animals were placed for 30 min into single housing cages (1284L Eurostandard type II cages, 42.5 × 27.6 × 15.3 cm, Tecniplast, Italy). After that the animals were transferred into individual motility boxes for 30 min where their motor activity was recorded after which the animals were returned to single cages. This approach helped to avoid the aggregation effect and increased aggressiveness among mice due to the strong stimulating effect of AMPH. It has been demonstrated, that AMPH is much more toxic to grouped mice than to mice housed singly in the individual cages (Chance, 1946). The development of hyperthermic condition in grouped mice is the reason for that (Hoehn and Lasagna, 1960). An additional argument for keeping mice separately is the increased aggressiveness induced by AMPH in male mice (Winslow and Miczek, 1983). Hodge and Butcher showed that d-AMPH tended to increase the frequency of fights in male mice at doses 0.5, 1, 2, and 4 mg/kg (Hodge and Butcher, 1975). Only after calming down from the stimulating effect of AMPH (no more than 30 min) the animals were returned to their home cages. Locomotor activity of individual mice was measured in a lit room (around 400 ± 25 lx) in sound-proof photoelectric motility boxes (44.8 × 44.8 × 45 cm) made of transparent Plexiglas and connected to a computer (TSE Technical & Scientific Equipment GmbH, Germany). After each mouse the floor of boxes was cleaned with 5% of ethanol solution. Software registered the distance traveled. Latin square design was used to randomize daily measurement cycles. On day 11, one of the saline groups received saline and the other AMPH (3 mg/kg). The latter group was used as a control for acute AMPH. Repeated AMPH group received AMPH (3 mg/kg) as usual. Immediately after the locomotor activity recordings, one by one (a time period between two mice was 8 min) animals were sacrificed by cervical dislocation, decapitated and trunk blood was collected for the metabolomic analysis.

### Sample collection

Blood sampling tubes were pre-processed with 20 μl of EDTA (ethylene-diamine-tetra-acetic acid). Tubes with blood samples were shaken and kept at room temperature for about 30 min, followed by centrifugation at 4°C 2,000 g for 10 min. Plasma was transferred to new tubes and stored at −80°C until use (Tuck et al., 2009).

### Measurement of metabolites in serum samples

The endogenous metabolites were analyzed with AbsoluteIDQ™ p180 Kit (Biocrates Life Sciences AG, Innsbruck, Austria). This validated assay allows comprehensive identification and quantification of amino acids, acylcarnitines, biogenic amines, hexoses, and phospho- and sphingolipids (phosphatidylcholines, lysophosphatidylcholines, sphingomyelins). Analyzed glycerophospholipids (lysophosphatidylcholines, phosphatidylcholines) are differentiated according to the presence of ester and ether bonds in the glycerol moiety. The “aa” indicates that fatty acids at the sn-1 and the sn-2 position are bound to the glycerol backbone via ester bonds, while “ae” denotes that fatty acid at the sn-1 position are bound via ether bond. The total number of carbon atoms and double bonds present in lipid's fatty acid chains are denoted as “C x:y,” where x indicates the number of carbons and y the number of double bonds. Serum levels of metabolites were determined using a flow injection analysis tandem mass spectrometry (FIA-MS/MS) as well as a liquid chromatography (LC-MS/MS) technique on a QTRAP 4500 mass-spectrometer (Sciex, USA). All preparations and measurements were performed as described in the manufacturer's kit manual. Identification and quantification of the metabolites were achieved using multiple reaction monitoring (MRM) along with internal standards. Calculations of metabolite concentrations were automatically performed by MetIDQ™ software (Biocrates Life Sciences AG, Innsbruck, Austria). Data quality was checked based both on the level of detection and the level of quantification (see also quality control data in Supplementary Table S4).

### Statistical analyses

Shapiro–Wilk test was applied to test for the normality assumption of data. The behavioral and body weight outcomes corresponded to the normal distribution. The distance traveled was analyzed by two-way ANOVA. The independent factors were strain (129Sv, Bl6) and administration (saline, acute AMPH, repeated AMPH) on the 11th day, followed by *post-hoc* unequal *N* Tukey HSD test. In all the following analyses, *p* < 0.05 was considered indicative of statistical significance. The body weight changes in 129Sv and Bl6 strain were analyzed by repeated measures ANOVA [strain × test day (1st and 11th day)], followed by *post-hoc* unequal *N* Tukey HSD test. The locomotor activity of 129Sv strong and weak responders was also analyzed with repeated measures ANOVA [subgroup × test day (1st and 11th day)], followed by *post hoc* Tukey HSD test. Since a part of metabolite data was not normally distributed, the Kruskal–Wallis analysis (multiple comparisons of mean ranks for all groups) was performed to analyze the effects of saline, acute and repeated AMPH on metabolite levels. Significant Kruskal–Wallis analysis was followed by Dunn's multiple comparison tests. The magnitudes of effect sizes were interpreted as moderate (eta-squared ranging 0.06–0.13) or large (eta-squared ≥ 0.14) (Cohen, 1992). The associations between distance traveled, metabolites and their ratios in repeated AMPH 129Sv mice were analyzed using the Spearman's rank correlation. Mann–Whitney *U*-test was applied to compare the raw data of two independent samples (strong and weak responders to repeated AMPH in 129Sv). A general linear model (GLM) multivariate analysis with a backward elimination procedure was performed to examine the associations between distance traveled, metabolites and their ratios in 129Sv mice responding differently to repeated AMPH. To normalize the distribution, we performed logarithmic transformation (log_10_) of the values of dependent characteristics prior to analysis. In the GLM analysis *p* < 0.05 was considered to be statistically significant. Partial eta^2^ value of ≥ 0.26 was defined as a large effect (Cohen, 1992). All the statistical analyses were performed using Statistica software (StatSoft Inc., 13th edition). Mean values and standard error means (SEM) are presented in figures.

### Ethics

All animal procedures in this study were performed in accordance with the European Communities Directive (2010/63/EU) and permit (No. 87, May 4, 2016) from the Estonian National Board of Animal Experiments.

## Results

### Amph-induced locomotor activity and body weight changes (Figures 2–5)

**Figure 2:**
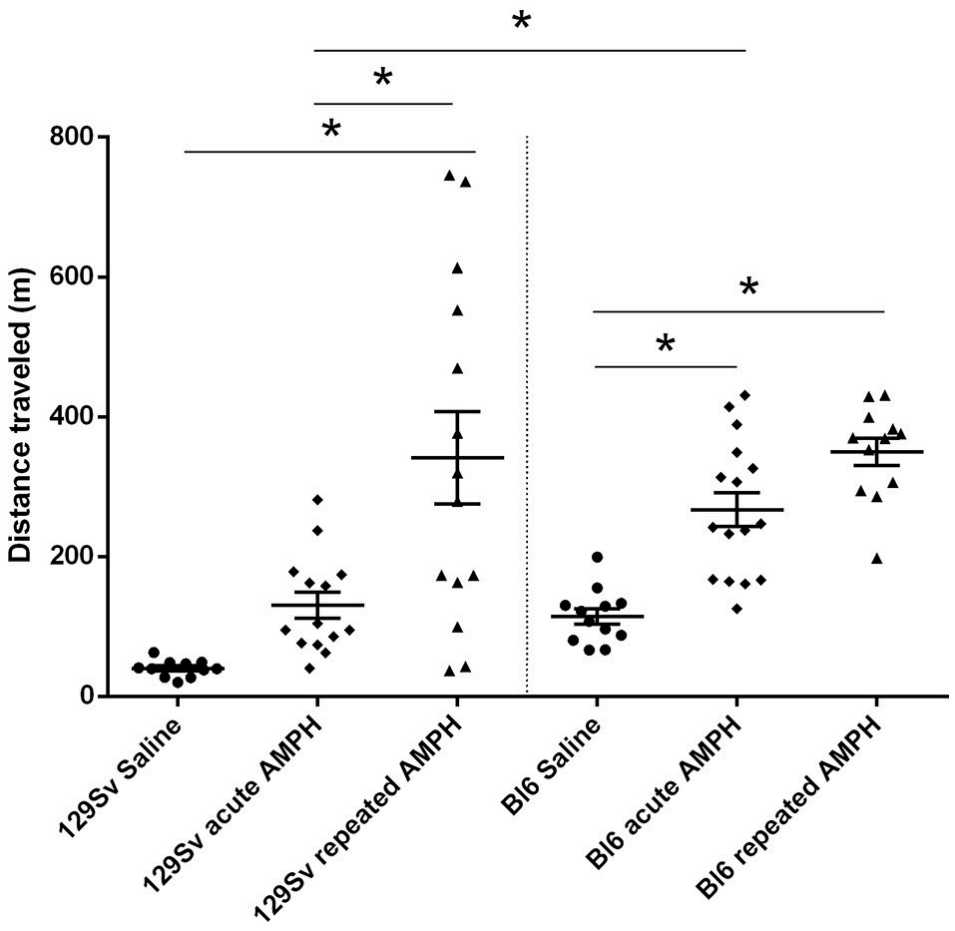
D-amphetamine induced motor sensitization in 129Sv and Bl6 (mean values ± SEM). Motor activity was analyzed using two-way ANOVA, followed by unequal Tukey HSD test. *p < 0.05 was considered statistically significant. More information about repeated testing can be found in the Supplementary Figure S1. Black circle, saline group; black diamond, acute AMPH group and black triangle, repeated AMPH group.

**Figure 3:**
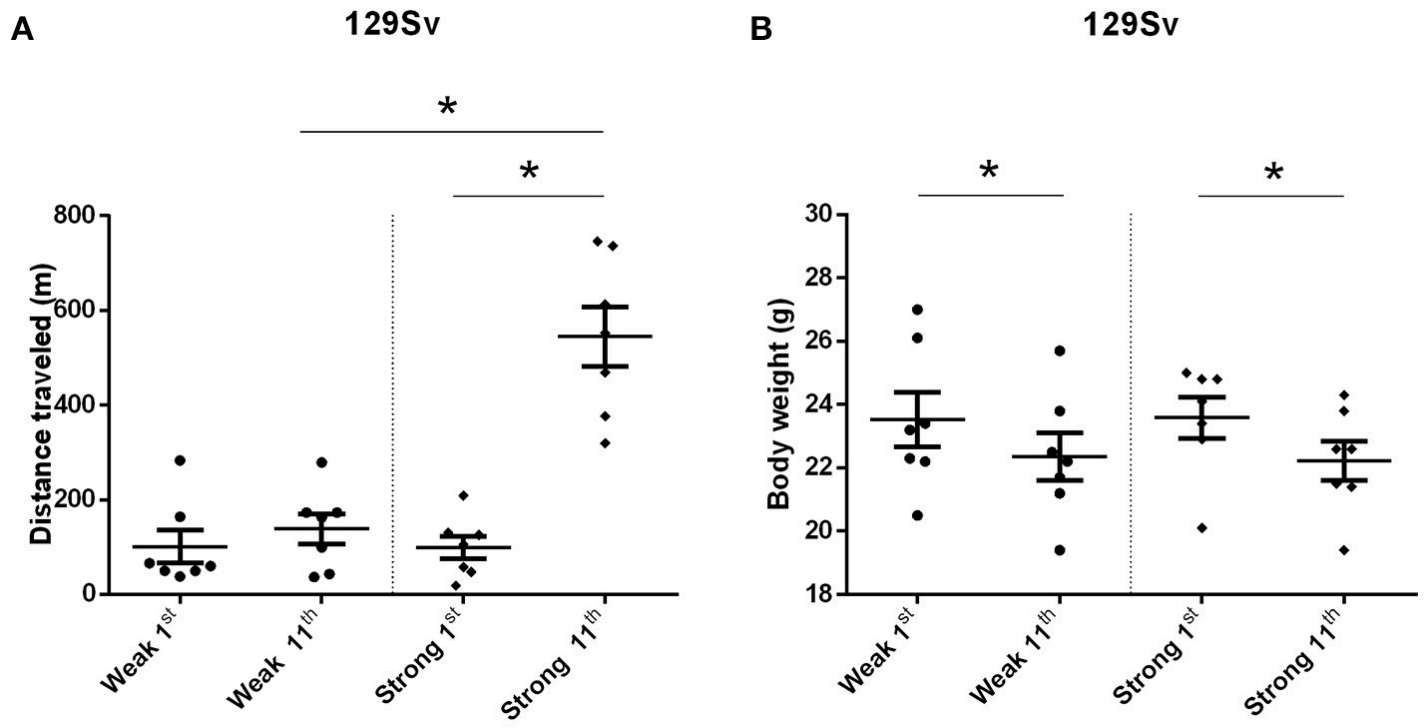
Motor sensitization **(A)** and body weight **(B)** in response to repeated AMPH administration in 129Sv weak and strong responders (mean values ± SEM). Strong responders displayed significantly greater sensitization to AMPH. Both weak and strong responders displayed loss of body weight. Motor activity and body weight outcomes were analyzed by repeated measures ANOVA, followed by Tukey HSD test. ^*^p < 0.05 was considered statistically significant. Black circle, weak responders; black diamond, strong responders.

The comparison of locomotor activity of age-matched 129Sv and Bl6 mice after acute and repeated AMPH demonstrated a significant difference between the two strains [two-way ANOVA: strain—*F*_(1, 73)_ = 7.12, *p* < 0.01; administration—*F*_(2, 73)_, *p* < 0.01; strain × administration—*F*_(2, 73)_, *p* = 0.015]. Acute AMPH (3 mg/kg) after repeated saline administration caused a statistically significant elevation of locomotor activity only in Bl6 (*p* = 0.03, unequal *N* Tukey HSD test), but not in 129Sv. However, after repeated AMPH both strains displayed a significant increase in distance traveled compared to saline (for 129Sv *p* < 0.01 and for Bl6 *p* < 0.01; Figure 2). The locomotor activity of 129Sv (342 ± 246 m) reached to the level of Bl6 (349 ± 65 m) after repeated AMPH. One has to take into account two peculiarities. First, the elevation of locomotor activity in 129Sv under the influence of repeated AMPH was more pronounced (2.6-fold increase compared to acute AMPH) compared to Bl6 mice (1.3-fold increase compared to acute AMPH). Second, we established greater locomotor activity dispersion around the mean value in 129Sv (*SD* = 246) compared to Bl6 (*SD* = 65). Therefore, two differently responding groups could be formed among 129Sv mice after repeated AMPH (Figure 3A), representing “weak responders” (*n* = 7) and “strong responders” (*n* = 7). In weak responders the distance traveled was 37–279 m and in strong responders 320–746 m (Figure 3A). Repeated measures ANOVA (subgroup × test day) demonstrated significant differences between weak and strong responders [subgroup: *F*_(1, 12)_ = 17.5, *p* < 0.01; test day: *F*_(1, 12)_ = 55.2, *p* < 0.01; subgroup × test day: *F*_(1, 12)_ = 39.7, *p* < 0.01]. *Post-hoc* analysis (Tukey HSD test) demonstrated significant differences between strong responders on the 1st and 11th day (*p* < 0.01), as well as between strong and weak responders on the 11th day (*p* < 0.01). No difference was established between weak activity subgroup on the 1st and 11th day (Figure 3A). The combination of distance traveled data from both strains (Bl6 + 129Sv) showed a clear AMPH effect on motor sensitization [*F*_(2, 76)_ = 26.8, *p* < 0.01; Figure 5A]. Acute AMPH caused a statistically significant elevation in distance traveled compared to saline (79 ± 10 vs. 203 ± 20 m, *p* < 0.01, unequal *N* Tukey HSD test). Repeated AMPH led to a further increase compared to acute AMPH in distance traveled (203 ± 20 vs. 345 ± 36 m, *p* < 0.01, unequal *N* Tukey HSD test).

**Figure 4:**
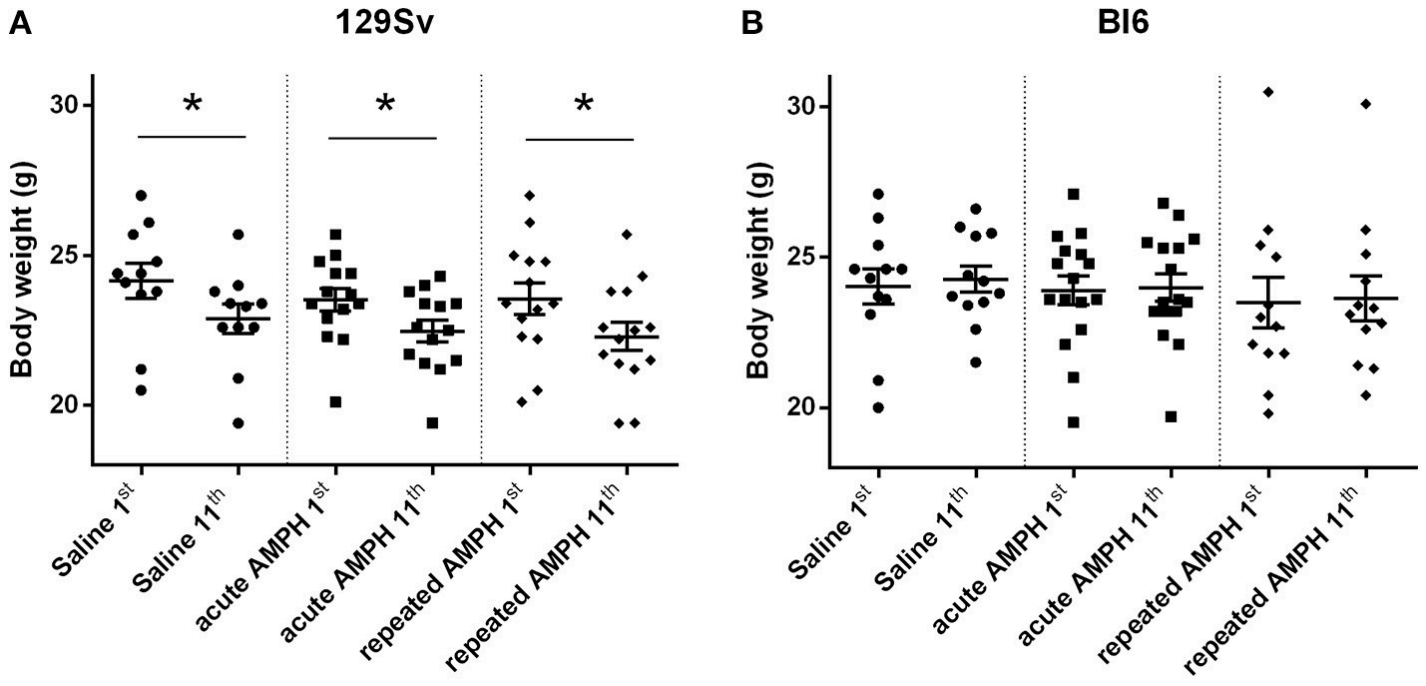
Body weight changes in 129Sv and Bl6 during the experiment (mean values ± SEM). Body weight outcomes were analyzed by two-way ANOVA, followed by unequal *N* Tukey HSD test. *p < 0.05 was considered statistically significant. In all groups of 129Sv strain a similar reduction of body weight was seen **(A)**, while repeated administrations did not cause any body weight changes in Bl6 **(B)**. Black circle - saline group; black square - acute AMPH group and black diamond - repeated AMPH group.

**Figure 5:**
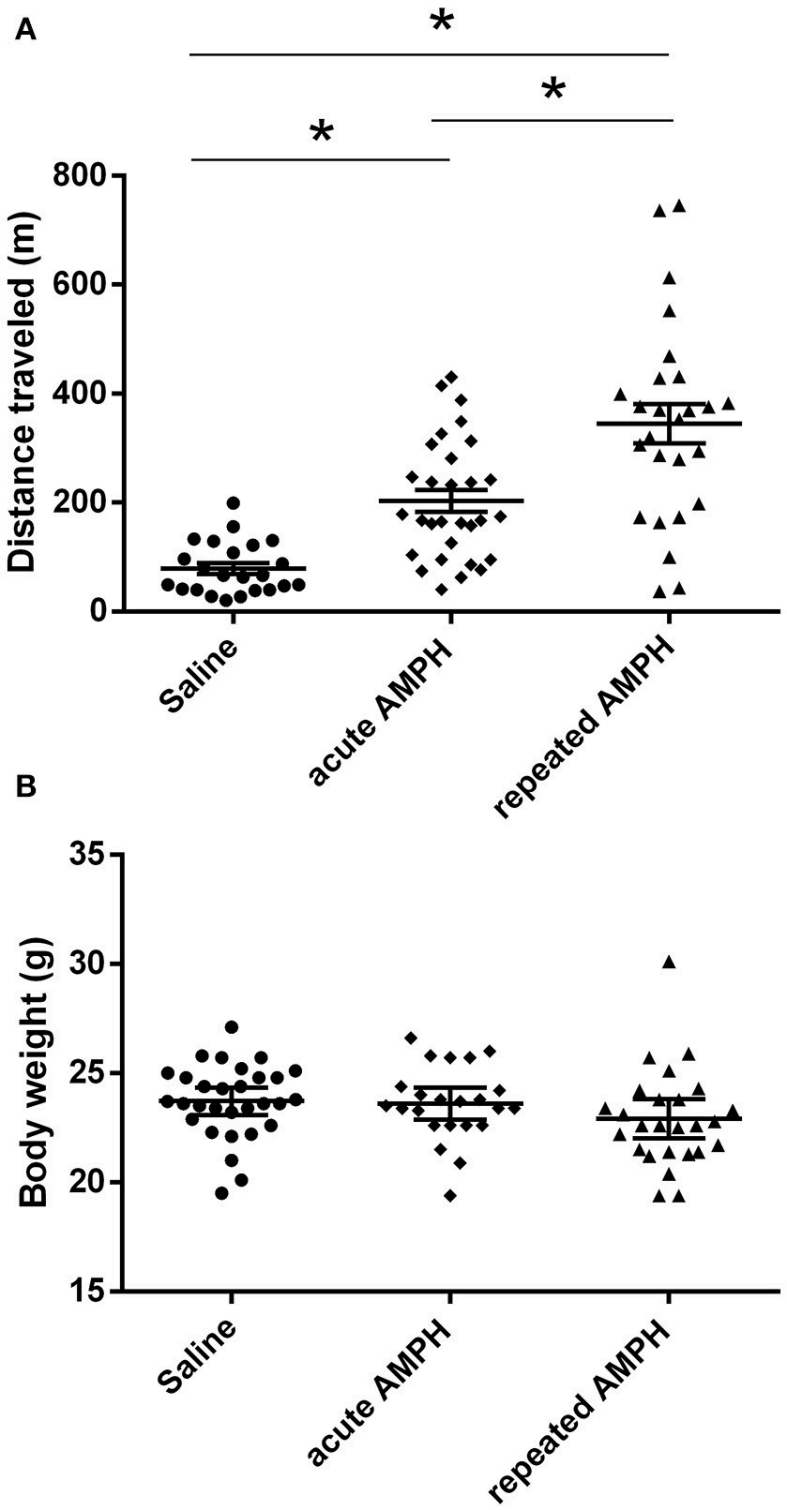
AMPH induced changes in distance traveled **(A)** and body weight **(B)** on the 11th day after pooling the data from both strains (Bl6+129Sv) (mean values ± SEM). The results were analyzed using one-way ANOVA, followed by unequal *N* Tukey HDS test. ^*^p < 0.05 was considered to be statistically significant. Black circle, saline administration; black diamond, acute AMPH administration and black triangle repeated AMPH administration.

Repeated measures ANOVA demonstrated significant differences in the body weight in 129Sv, but not in Bl6 mice if measured on the 1st and 11th day [repeated measures ANOVA: test day *F*_(1, 73)_ = 49.4, *p* < 0.01; test day x strain *F*_(1, 73)_ = 86.7, *p* < 0.01]. Repeated saline and AMPH did not cause any remarkable changes in the body weight of Bl6 (Figure 4B). However, in 129Sv repeated saline and AMPH caused a similar reduction of body weight in all groups showing that this decrease was not caused by AMPH, but by the experimental conditions (Figure 4A). Moreover, there was no difference in body weight between weak and strong responders to AMPH in the 129Sv group (Figure 3B). Combination of body weight data from both strains (Bl6 +129Sv) did not establish any significant differences [*F*_(2, 76)_ = 0.81, *p* = 0.45]: saline −23.6 ± 0.36, acute AMPH −23.3 ± 0.32, repeated AMPH −22.9 ± 0.44 (Figure 5B).

### Preliminary analysis of metabolomic markers

The established manipulations induced distinct behavioral responses in Bl6 and 129Sv. Thus, a preliminary analysis was performed to evaluate the impact of repeated saline administration on metabolic markers. The results indicated that altogether 52 metabolites were significantly different between 129Sv and Bl6 (Narvik et al., 2018). The values of 33 items were higher in Bl6 and 19 items were respectively elevated in 129Sv. Therefore, the distinct behavioral strategies of Bl6 and 129Sv are likely associated with significant differences in their metabolism. This justifies the use of two mice strains separately in the initial metabolomic analysis of repeated AMPH. Finally, the metabolic data from two lines were pooled and subjected to statistical analysis to establish the effect of repeated AMPH independent from the strain background.

### AMPH-induced changes in metabolites in strains

#### AMPH-induced changes in metabolites in Bl6 (Table 1)

Acute AMPH caused a significant elevation of isoleucine and leucine, two representatives of branched chain amino acids (BCAA). These amino acids were clearly higher in Bl6 receiving acute AMPH. Besides that, there was a significant shift toward favoring BCAA compared to aromatic amino acids (AAA). These effects were not further modified by repeated AMPH. Therefore, a similar elevation of isoleucine and leucine was established for both acute and repeated AMPH. Simultaneously, the levels of several biogenic amines (ADMA, alpha-AAA, kynurenine) and hexoses were significantly reduced after repeated AMPH (Table 1). The ratio between amino acids glycine and serine was also significantly decreased after acute AMPH (Table 1).

**Table 1:** D-amphetamine-induced statistically significant metabolite changes (μmoles, median and range) and their ratios in Bl6 strain (Kruskal–Wallis test, *p* < 0.05).

**Metabolites and their ratios**	**Saline (*N* = 12)**	**Acute AMPH (*N* = 16)**	**Repeated AMPH (*N* = 12)**	**Kruskal–Wallis test**	**Effect size (η^2^)**
Hexoses	8,569 7,452–11,103	7,407^a^ 4,459–9,751	7,343^c^ 3,810–9,625	$\chi_{\left(2,\;39\right)}^{2}$ = 6.34, *p* = 0.04	0.14
Isoleucine	83.7 60.6–108	105^a^ 75.9–214	104^c^ 62.5–208	$\chi_{\left(2,\;40\right)}^{2}$ = 8.98, *p* = 0.01	0.18
Leucine	123 92.8–159	158^a^ 112–372	160^c^ 104–361	$\chi_{\left(2,\;39\right)}^{2}$ = 10.87, *p* = 0.004	0.22
ADMA	0.36 0.21–0.75	0.40 0.24–0.80	0.23^b^^,^^c^ 0.000–0.60	$\chi_{\left(2,\;37\right)}^{2}$ = 8.75, *p* = 0.01	0.19
Alpha-aminoadipic acid	11.0 7.42–17.2	10.2 0.000–16.0	8.63^c^ 4.46–10.3	$\chi_{\left(2,\;36\right)}^{2}=$7.66, *p* = 0.02	0.18
Kynurenine	1.40 0.93–1.56	1.17 0.89–1.58	1.07^c^ 0.83–1.90	$\chi_{\left(2,\;38\right)}^{2}$ = 7.10, *p* = 0.03	0.16
BCAA	373 308–467	447^a^ 309–985	441 298–939	$\chi_{\left(2,\;40\right)}^{2}=$7.84, *p* = 0.02	0.16
BCAA/AAA	1.76 1.45–2.10	1.93^a^ 1.65–2.49	2.06^c^ 1.47–2.46	$\chi_{\left(2,\;40\right)}^{2}$ = 10.47, *p* = 0.005	0.21
Glycine/Serine	3.72 1.70–4.39	2.52^a^ 1.29–3.74	3.00 1.65–8.46	$\chi_{\left(2,\;40\right)}^{2}$ = 8.25, *p* = 0.02	0.17

*Effect size estimates for chi-square values are indicated by η^2^, where value ≥ 0.14 corresponds to a large effect*.

a *Statistically significant difference (p < 0.05) between saline and acute AMPH*.

b *Statistically significant difference (p < 0.05) between acute AMPH and repeated AMPH*.

c *Statistically significant difference (p < 0.05) between saline and repeated AMPH*.

#### AMPH-induced changes in metabolites in 129Sv (Table 2)

The pattern of altered metabolites in 129Sv was vastly different from that seen in Bl6. In 129Sv, acute AMPH induced a significant reduction in several metabolites compared to saline, including valine, lysoPCs (lyso PC aa 16:0, lyso PC aa 18:2, lyso PC aa 20:4), PC diacyls (PC aa 34:2, PC aa 36:2, PC aa 36:3, PC aa 36:4) and PC acyl-alkyls (PC ae 38:4 and PC ae 40:4). Moreover, several metabolites and their ratios were elevated if the effect of acute and repeated AMPH was compared in 129Sv. The list of metabolites includes long chain acylcarnitines (C14, C14:1-OH, C16, C16:1, C18:1), BCAAs (particularly isoleucine, valine), PC diacyls (PC aa C38:4, PC aa C38:6, PC aa C42:6), PC acyl-alkyls (PC ae C38:4, PC ae C40:4, PC ae C40:5, PC ae C40:6, PC ae C42:1, PC ae C42:3), and sphingolipids [SM(OH)C22:1, SM C24:0]. The list of elevated ratios includes acylcarnitines C5/carnitine, CPT-1 ratio, glycine/glutamine, and lysoPC a C20:4/lysoPC a C20:3. Comparably fewer markers were affected if repeated AMPH was compared to saline. A limited number of metabolites and their ratios were significantly reduced (hexoses, kynurenine, PC aa C36:3, PC aa C36:3/PC aa C36:4) or elevated (PC aa C32:0, C3/carnitine, lysoPC a C20:4/lysoPC a C20:3) in this comparison (Table 2). Therefore, one can conclude that acute AMPH caused a substantial reduction of several metabolites and ratios (altogether 10 items) compared to saline, whereas the effect of repeated AMPH was more frequently associated with the elevation of metabolites (altogether 22 items) compared to acute AMPH. When repeated AMPH group was compared to the saline group, the changes were less numerous (altogether 8 items).

**Table 2:** D-amphetamine-induced statistically significant metabolite (μmoles, median, and range) changes and their ratios in 129Sv strain (Kruskal–Wallis test, *p* < 0.05).

**Metabolites and their ratios**	**Saline (*N* = 11)**	**Acute AMPH (*N* = 14)**	**Repeated AMPH (*N* = 14)**	**Kruskal–Wallis test**	**Effect size (η^2^)**
C14	0.081 0.067–0.10	0.071 0.054–0.13	0.10^b^ 0.069–0.16	$\chi_{\left(2,\;39\right)}^{2}$ = 9.71, *p* = 0.008	0.20
C14:1-OH	0.013 0.000–0.019	0.000 0.000–0.020	0.016^b^ 0.000–0.025	$\chi_{\left(2,\;39\right)}^{2}$ = 11.28, *p* = 0.004	0.22
C16	0.27 0.21–0.34	0.24 0.17–0.38	0.31^b^ 0.23–0.41	$\chi_{\left(2,\;38\right)}^{2}$ = 11.25, *p* = 0.004	0.23
C16:1	0.078 0.059–0.098	0.068 0.039–0.12	0.096 ^b^ 0.067–0.14	$\chi_{\left(2,\;38\right)}^{2}$ = 7.86, *p* = 0.02	0.17
C18:1	0.15 0.14–0.20	0.14 0.11–0.26	0.20^b^ 0.13–0.23	$\chi_{\left(2,\;38\right)}^{2}$ = 9.94, *p* = 0.007	0.19
Hexoses	5,810 4,405–8,005	4,299 3,269–6,617	4,549^c^ 2,764–5,937	$\chi_{\left(2,\;38\right)}^{2}$ = 8.21, *p* = 0.02	0.18
Isoleucine	97.3 79.8–129	92.6 75.6–120	121^b^ 69.4–203	$\chi_{\left(2,\;38\right)}^{2}$ = 6.92, *p* = 0.03	0.15
Valine	192 146–297	149^a^ 117–232	198^b^ 115–275	$\chi_{\left(2,\;38\right)}^{2}$ = 7.88, *p* = 0.02	0.17
Kynurenine	1.43 1.13–1.90	1.24 0.94–1.64	1.27^c^ 1.05–1.45	$\chi_{\left(2,\;37\right)}^{2}$ = 6.10, *p* = 0.047	0.14
LysoPC a C16:0	292 179–444	211^a^ 143–366	268 121–333	$\chi_{\left(2,\;39\right)}^{2}$ = 6.81, *p* = 0.03	0.15
LysoPC a C18:2	133 88.0–181	90.1^a^ 61.1–169	107 56.3–122	$\chi_{\left(2,\;39\right)}^{2}$ = 9.09, *p* = 0.01	0.19
LysoPC a C20:4	29.3 20.2–44.0	20.6^a^ 14.8–34.9	28.7 16.4–34.7	$\chi_{\left(2,\;39\right)}^{2}$ = 7.57, *p* = 0.02	0.16
PC aa C32:0	10.8 8.34–15.9	12.8 9.65–16.4	15.9 ^c^ 8.05–29.0	$\chi_{\left(2,\;38\right)}^{2}$ = 7.12, *p* = 0.03	0.16
PC aa C34:2	263 189–356	202^a^ 126–343	235 123–294	$\chi_{\left(2,\;39\right)}^{2}$ = 6.95, *p* = 0.03	0.15
PC aa C36:2	175 111–214	114 ^a^ 77.8–211	143 74.6–185	$\chi_{\left(2,\;39\right)}^{2}$ = 8.57, *p* = 0.01	0.18
PC aa C36:3	58.6 39.1–78.1	44.3^a^ 28.5–76.5	46.6^c^ 27.4–58.2	$\chi_{\left(2,\;39\right)}^{2}$ = 8.36, *p* = 0.02	0.18
PC aa C36:4	88.9 65.4–116	65.6^a^ 43.1–125	85.2 52.9–106	$\chi_{\left(2,\;39\right)}^{2}$ = 7.98, *p* = 0.02	0.17
PC aa C38:4	44.6 35.9–74.0	37.1 28.9–73.2	55.5^b^ 34.9–76.5	$\chi_{\left(2,\;39\right)}^{2}$ = 9.57, *p* = 0.008	0.19
PC aa C38:6	68.2 49.2–99.2	51.9 39.4–91.8	71.8^b^ 40.0–87.5	$\chi_{\left(2,\;39\right)}^{2}$ = 6.65, *p* = 0.04	0.15
PC aa C42:6	0.78 0.61–1.07	0.64 0.48–0.96	0.81^b^ 0.49–1.17	$\chi_{\left(2,\;39\right)}^{2}$ = 8.13, *p* = 0.017	0.17
PC ae C38:4	2.55 1.84–3.24	1.93^a^ 1.35–2.78	2.69 ^b^ 1.80–3.71	$\chi_{\left(2,\;38\right)}^{2}$ = 13.05, *p* = 0.002	0.26
PC ae C40:4	1.52 1.15–1.88	1.17^a^ 0.84–1.67	1.59^b^ 1.07–2.13	$\chi_{\left(2,\;38\right)}^{2}$ = 10.20, *p* = 0.006	0.21
PC ae C40:5	0.78 0.63–1.17	0.71 0.50–0.84	0.88^b^ 0.63–1.09	$\chi_{\left(2,\;38\right)}^{2}$ = 9.37, *p* = 0.009	0.20
PC ae C40:6	1.61 1.14–2.11	1.20 0.95–1.80	1.78^b^ 1.08–2.53	$\chi_{\left(2,\;38\right)}^{2}$ = 10.47, *p* = 0.005	0.22
PC ae C42:1	0.46 0.41–0.68	0.40 0.32–0.58	0.54^b^ 0.38–0.71	$\chi_{\left(2,\;38\right)}^{2}$ = 10.00, *p* = 0.007	0.21
PC ae C42:3	0.55 0.46–0.90	0.48 0.33–0.77	0.64^b^ 0.42–0.80	$\chi_{\left(2,\;38\right)}^{2}$ = 9.72, *p* = 0.008	0.20
SM(OH) C22:1	1.44 0.91–1.91	1.15 0.733–1.64	1.53^b^ 0.81–2.34	$\chi_{\left(2,\;38\right)}^{2}$ = 9.48, *p* = 0.009	0.20
SM C24:0	4.42 2.96–6.02	3.70 3.10–4.95	4.46^b^ 2.59–7.02	$\chi_{\left(2,\;38\right)}^{2}$ = 10.41, *p* = 0.006	0.21
BCAA	428 349–611	375 305–564	494^b^ 291–853	$\chi_{\left(2,\;38\right)}^{2}$ = 6.48, *p* = 0.04	0.15
C3/C0	0.028 0.018–0.037	0.028 0.021–0.038	0.038^c^ 0.029–0.045	$\chi_{\left(2,\;38\right)}^{2}$ = 7.00, *p* = 0.03	0.15
C5/C0	0.015 0.010–0.024	0.014 0.011–0.020	0.019^b^ 0.009–0.036	$\chi_{\left(2,\;39\right)}^{2}$ = 7.53, *p* = 0.02	0.16
C4/C5	2.92 1.84–3.95	2.91 2.02–4.03	2.44^c^ 1.84–3.32	$\chi_{\left(2,\;38\right)}^{2}$ = 6.69, *p* = 0.04	0.15
^*^CPT1 ratio	0.011 0.007–0.020	0.009 0.005–0.020	0.014^b^ 0.008–0.027	$\chi_{\left(2,\;39\right)}^{2}$ = 11.13, *p* = 0.004	0.22
Glycine/Glutamine	0.42 0.30–0.51	0.38 0.27–0.60	0.47^b^ 0.37–0.61	$\chi_{\left(2,\;39\right)}^{2}$ = 7.04, *p* = 0.03	0.15
LysoPC a C20:4/LysoPC a C20:3	3.29 3.07–4.06	3.30 2.76–4.07	4.09^b^^,^^c^ 3.39–5.71	$\chi_{\left(2,\;39\right)}^{2}$ = 15.07, *p* = 0.0005	0.28
PC aa C36:3/PC aa C36:4	0.67 0.53–0.85	0.66 0.549–0.86	0.52^b^^,^^c^ 0.46–0.67	$\chi_{\left(2,\;39\right)}^{2}$ = 18.39, *p* = 0.0001	0.32

*Effect size estimates for chi-square values are indicated by η^2^, where value ≥ 0.14 corresponds to a large effect*.

* *CPT1 (carnitine palmitoyltransferase 1) ratio [(C16 + C18)/carnitine]*.

a *Statistically significant difference (p < 0.05) between saline and acute AMPH*.

b *Statistically significant difference (p < 0.05) between acute AMPH and repeated AMPH*.

c *Statistically significant difference (p < 0.05) between saline and repeated AMPH*.

#### Metabolites associated with different response to AMPH in 129Sv (Tables 3, 4, Supplementary Table S3)

Among strong responders to AMPH in 129Sv the levels of several long chain acylcarnitines were significantly elevated compared to weak responders: C12, C14, C14:1, C14:1-OH, and C16:1 (Table 3). Besides that the level of hexoses was significantly reduced in strong compared to weak responders, reflecting an apparent link between the intensity of locomotor activity and hexoses metabolism. A similar inhibition was established for PC aa C36:3, ratio acylcarnitines C4/C5, and ratio glycine/glutamine if the strong and weak responders were compared.

**Table 3:** Distance traveled (m), metabolites (μmoles), and their ratios (median and range) in 129Sv responding differently to d-amphetamine: weak and strong responders (Mann–Whitney *U-*test, *p* < 0.05).

	**Weak (*N* = 7)**	**Strong (*N* = 7)**	***Z*-value**	***p*-value**	**Effect size (η^2^)**
Distance traveled on day 11	163 37–279	552 320–746	−3.07	0.002	0.67
C12	0.10 0.000–0.13	0.12 0.096–0.16	−2.04	0.04	0.30
C14:1	0.058 0.040–0.069	0.069 0.059–0.089	−2.11	0.04	0.32
C14:1-OH	0.013 0.000–0.018	0.016 0.013–0.025	−1.98	0.05	0.28
C16:1	0.085 0.067–0.11	0.10 0.083–0.14	−2.04	0.04	0.30
PC aa C36:3	50.9 29.1–58.2	42.6 27.4–47.9	2.04	0.04	0.30
Hexoses	5,551 2,764–5,937	3,792 3,284–4,787	2.04	0.04	0.30
C4/C5	2.82 1.88–3.32	2.25 1.84–2.55	2.17	0.03	0.34
Glycine/Glutamine	0.55 0.45–0.61	0.45 0.37–0.49	2.43	0.02	0.42

*Effect size estimates are indicated by η^2^, where partial eta^2^ value ≥ 0.26 was defined as a large effect*.

**Table 4:** Main effect of repeated AMPH administration on distance traveled (m), metabolite levels (μmoles), and their ratios in 129Sv.

	**ß**	**ß (95 % CI)**	***t*-value**	***p*-value**
Distance traveled on day 11	−0.82	−1.18, −0.45	−4.91	0.0004
C14:1	−0.55	−1.08, −0.03	−2.31	0.04
C16	−0.58	−1.09, −0.06	−2.45	0.03
C16:1	−0.65	−1.13, −0.17	−2.96	0.01
C18:1	−0.60	−1.10, −0.10	−2.62	0.02
Glycine/Glutamine	0.73	0.30, 1.16	3.71	0.003

*Regression coefficients (ß) and significant values of log10-transformed variables. CI, confidence intervals*.

To confirm the existence of different responders (in terms of behavior and metabolic parameters) to repeated AMPH in 129Sv we used the GLM. Using a model fit criterion, candidate variables that did not contribute to the model were removed at a 5% significance level. The final parsimonious model (Table 4) retained the distance traveled on day 11, C14:1, C16, C16:1, C18:1 and the ratio glycine/glutamine as significant predictors to distinguish between subgroups.

Spearman rank correlation (Supplementary Table S3) established a positive correlation of AMPH-induced locomotor activity with C16, C16:1 and ratio between tyrosine and phenylalanine. The ratio glycine/histidine, as well as the ratio acylcarnitines C4/C5 were correlated negatively with AMPH-induced locomotor activity. Among others, the most prominent relationship (ρ = −0.96) was found between C18:1 and hexoses, i.e. the animals with the lowest levels of hexoses displayed the highest levels of C18:1.

### AMPH-induced changes in metabolites independent from the strain (Table 5)

Despite significant basal differences between 129Sv and Bl6, combining the data from these two strains revealed 14 metabolites and their ratios which remained significant when Kruskal–Wallis ANOVA test was applied (Table 5). The change of one metabolite (reduction of kynurenine) displayed a large effect size (η^2^ = 0.15), whereas the other effects were in the moderate range (η^2^ = 0.06–0.13). The levels of BCAA (leucine, isoleucine) and the ratio lysoPC a C20:4/lysoPC a C20:3 were markedly increased by repeated AMPH. By contrast, the levels of citrulline, ADMA, hexoses and lysoPC a C18:2 were significantly reduced in repeated AMPH. Long-chain acylcarnitines (CPT-1 ratio, C14) and PC alkyl-acyls (PC ae 40:6, PC ae 42:1) displayed an elevation if acute and repeated AMPH were compared.

**Table 5:** D-amphetamine-induced statistically significant changes of metabolites (μmoles, median, and range) if both strains were analyzed together (Kruskal–Wallis test, *p* < 0.05).

**Metabolites and their ratios**	**Saline (*N* = 23)**	**Acute AMPH (*N* = 30)**	**Repeated AMPH (*N* = 26)**	**Kruskal–Wallis test**	**Effect size *(*η^2^)**
C14	0.088 0.06–0.12	0.075 0.054–0.14	0.094^b^ 0.059–0.18	$\chi_{\left(2,\;79\right)}^{2}$ = 6.19, p = 0.045	0.07
Hexoses	7,609 4,405–11,103	6,053^a^ 3,269–9,751	5,778^c^ 2,764–9,625	$\chi_{\left(2,\;77\right)}^{2}$ = 7.31, *p* = 0.03	0.09
Citrulline	51.4 28.9–112	45.2^a^ 14.2–112	41.5^c^ 27.5–82.0	$\chi_{\left(2,\;78\right)}^{2}$ = 8.51, *p* = 0.01	0.10
Isoleucine	91.5 60.6–129	95.0 75.6–214	109^c^ 62.5–208	$\chi_{\left(2,\;79\right)}^{2}$ = 8.66, *p* = 0.013	0.10
Leucine	129 92.8–197	154^a^ 107–372	168^c^ 104–413	$\chi_{\left(2,\;79\right)}^{2}$ = 11.75, *p* = 0.003	0.13
ADMA	0.34 0.11–0.75	0.34 0.00–1.27	0.26^c^ 0.00–0.60	$\chi_{\left(2,\;76\right)}^{2}$ = 6.70, *p* = 0.04	0.08
Kynurenine	1.42 0.93–1.90	1.22^a^ 0.89–1.6	1.15^c^ 0.83–1.90	$\chi_{\left(2,\;75\right)}^{2}$ = 12.82, *p* = 0.002	0.15
lysoPC a C16:0	302 158–444	225^a^ 127–411	268 121–443	$\chi_{\left(2,\;79\right)}^{2}$ = 8.42, *p* = 0.02	0.10
lysoPC a C18:2	151 88.0–198	113^a^ 61.1–242	114^c^ 56.3–226	$\chi_{\left(2,\;78\right)}^{2}$ = 10.42, *p* = 0.006	0.12
PC ae C40:6	1.48 0.82–2.11	1.21 0.58–1.80	1.47^b^ 0.80 - 2.53	$\chi_{\left(2,\;78\right)}^{2}$ = 6.82, *p* = 0.03	0.08
PC ae C42:1	0.47 0.22–0.68	0.42 0.17–0.66	0.51^b^ 0.32–0.80	$\chi_{\left(2,\;78\right)}^{2}$ = 7.01, *p* = 0.03	0.08
BCAA	397 308–611	420 305–811	461^c^ 291–939	$\chi_{\left(2,\;78\right)}^{2}$ = 6.03, *p* = 0.049	0.07
*CPT1 ratio	0.009 0.005–0.020	0.009 0.005–0.020	0.011^b^ 0.004–0.030	$\chi_{\left(2,\;79\right)}^{2}$ = 8.92, *p* = 0.01	0.10
lysoPC a C20:4/lysoPC a C20:3	3.35 2.85–4.47	3.43 2.66–5.89	3.82^b^^,^^c^ 2.91–5.71	$\chi_{\left(2,\;79\right)}^{2}$ = 8.59, *p* = 0.01	0.10

Effect size estimates for chi-square values are indicated by η^2^, where value ≥ 0.14 corresponds to a large effect. *CPT1 (carnitine palmitoyltransferase 1) ratio [(C16 + C18)/carnitine].

a *Statistically significant difference (p < 0.05) between saline and acute AMPH*.

b *Statistically significant difference (p < 0.05) between acute AMPH and repeated AMPH*.

c *Statistically significant difference (p < 0.05) between saline and repeated AMPH*.

## Discussion

Repeated administration of AMPH has been used to model psychotic-like behavior in rodents. So far, few studies have been performed to examine mouse strain differences in behavioral sensitization to AMPH. After a thorough literature search we may conclude that we are the first to explore the metabolic profile of these two mouse lines. Furthermore, we have recently shown that after repeated saline administration the 129Sv and Bl6 strains display different metabolic profiles and behavioral coping strategies (Narvik et al., 2018). This encouraged us to investigate the metabolic outcomes of repeated AMPH in these two mouse strains. We found that the effect of genetic background exceeds that of pharmacological influence. Besides 129Sv displayed a significantly larger variation after repeated AMPH than Bl6. Based on our results we believe 129Sv to be a more promising strain for evaluating psychotic-like behaviors compared to Bl6. Starting from the genetic point of view 129Sv mice have mutated DISC1 protein, strongly affecting DA homeostasis (Clapcote and Roder, 2006; Dahoun et al., 2017). Recent clinical research relates DISC1 mutations to various neuropsychiatric disorders (Thomson et al., 2016). The mutation in the *Disc1* gene is the first aspect why 129Sv mice could be better models for studying psychotic-like behavior than the Bl6 strain. Second, 129Sv mice display aberrant adaptation in a stressful environment. Repeated testing of Bl6 mice in the motility cages robustly increased their exploratory activity, whereas in 129Sv no such changes occurred and their activity remained almost at the level of the first testing day (Narvik et al., 2018). However, as opposed to Bl6 mice, 129Sv mice started to lose body weight. One could suggest that these differences are related to the prevailing coping strategies in these two strains due to variations in the function of the DA system. One may speculate that the retarded behavior of 129Sv mice in stressful situations may to a certain extent, reflect the characteristics of the prodrome syndrome of first episode psychosis in humans. Third, vast differences between 129Sv and Bl6 mice could be seen after repeated AMPH administration. In Bl6 mice only moderate sensitization toward AMPH was observed, whereas 129Sv mice could be divided into two subgroups. In one subgroup repeated AMPH failed to magnify the drug effect compared to the acute AMPH, whereas in the other subgroup an almost 5-fold sensitization was established.

Altogether, 129Sv seems to be a better model than Bl6 for modeling certain neuropsychiatric disorders. 129Sv have mutated *Disc1* gene, display retarded adaptation in stressful environments and their response to repeated administration of AMPH is vastly deviated. One subgroup displays no sensitization to AMPH, resembling depression-like state, whereas the other subgroup responds with the robust sensitization, resembling psychotic-like state. Depressive and psychotic symptoms both can be seen in patients with the first episode psychosis. These large variations in behavioral outcome in 129Sv mice having the same genetic background is rather unexpected and definitely needs further analysis.

### AMPH-induced behavioral and body weight differences

The current study revealed that 129Sv and Bl6 demonstrate vastly different motor responses to AMPH administration (3 mg/kg i.p.). The increase of locomotor activity was significantly stronger in acutely treated Bl6 (Figure 2) compared to 129Sv. This is line with the existing evidence that Bl6 is more responsive to the stimulating effect of acute AMPH. This response can be attributed to the greater activity of the DA-ergic system in these animals compared to 129Sv (Chen et al., 2007). After repeated AMPH both strains displayed a significant increase in distance traveled (Figure 2, Supplementary Figure S1A). In fact, the locomotor activity of 129Sv reached to the level of Bl6 after repeated AMPH. Elevation of locomotor activity in response to repeated AMPH was more pronounced in 129Sv compared to Bl6 mice. Also, we established greater locomotor activity dispersion around the mean value in 129Sv compared to Bl6. Therefore, two differently responding groups can be formed among 129Sv receiving repeated AMPH: one which responded similarly to the acute AMPH group (weak responders), and the other one in which the response was 5-fold augmented (strong responders; Figure 3A). Moreover, we found that 129Sv responded to the daily manipulations with loss of body weight (Figure 4A). However, this effect was not due to AMPH, because it was similar in all administration groups. This demonstrates that the applied dose of AMPH does not suppress food intake in 129Sv. Opposite to 129Sv, no body weight decline occurred in Bl6 (Figure 4B). Overall, this indicates that behavioral manipulations were more stressful for 129Sv than for Bl6. This is in line with our previous studies showing that exposing mice to behavioral enrichment induces a reduction of body weight in 129Sv, but not in Bl6 in subsequent behavioral tests (Heinla et al., 2014).

### AMPH-induced metabolite differences

A profound difference between 129Sv and Bl6 was revealed not only at the behavioral and body weight levels, but the metabolites were also differently affected by AMPH in these two mouse strains (Supplementary Figures S2, S3). The number of affected metabolites in Bl6 was less pronounced compared to 129Sv. Nevertheless, several significant changes were established after acute AMPH. This involves an apparent elevation of BCAA levels in Bl6—isoleucine and leucine (Table 1). This alteration in the levels of isoleucine and leucine was accompanied by a shift in the ratio between BCAA and AAA favoring the former ones. Besides that, there was a trend for the reduction of hexoses (Table 1, Supplementary Table S1). After repeated AMPH no further increase was established in leucine and isoleucine levels. One could suggest that these metabolic changes may reflect behavioral changes due to AMPH administration. Acute AMPH caused in Bl6 a profound elevation of locomotor activity, causing an increased need for energy (Figure 2). Therefore, the trend for declined levels of hexoses (including glucose) possibly reflects this need (Table 1). To replenish the energetic need due to increased workload, isoleucine and leucine were used as additional energetic sources. Besides that, the levels of biogenic amines were reduced by repeated AMPH in Bl6, including ADMA, alpha-AAA and kynurenine. ADMA, an analog of L-arginine, is a naturally occurring product of metabolism found in circulation. Elevated levels of ADMA inhibit NO synthesis and, therefore, lead to impaired endothelial function (Sibal et al., 2010). Dimethylarginine dimethylaminohydrolase (DDAH) has been shown to hydrolyse ADMA to yield citrulline and dimethylamine (Leiper and Vallance, 2006). Therefore, the formation of NO from arginine is not the only source for the production of citrulline. Xuan and colleagues demonstrated the antagonistic function of citrulline against ADMA, showing protection of endothelium from impairment of ADMA in porcine coronary arteries (Xuan et al., 2015). The beneficial effect of citrulline against ADMA on endothelial function may be attributed to the preservation of NO production, activation of the NO/cGMP signaling pathway, and suppression of superoxide anion overproduction (Xuan et al., 2015).

Alpha-AAA is a component of lysine metabolism pathway and a marker of oxidative stress (Yuan et al., 2011; Zeitoun-Ghandour et al., 2011). A recent metabolomic study of diabetes patients plasma samples suggested that alpha-AAA may be a modulator of glucose homeostasis and diabetes risk (Wang et al., 2013). Studies in rodents have also shown that alpha-AAA modulates kynurenic acid levels in the brain. Kynurenic acid is a neuroactive metabolite that interacts with NMDA, α-amino-3-hydroxy-5-methyl-4-isoxazolepropionic acid (AMPA)/kainate and alpha 7 nicotinic receptors (Gramsbergen et al., 1997). In experiments with rat brain tissue slices, alpha-AAA exposure resulted in a substantial decrease in the levels of kynurenic acid (Wu et al., 1995). Similarly, *in vivo* studies in free-moving rats exposed to alpha-AAA through microdialysis in the hippocampus resulted in a robust decrease in kynurenic acid level (Chang et al., 1997). Alpha-AAA is a substrate of the enzyme alpha-AAA aminotransferase II, which has been shown to be the same enzyme as kynurenine aminotransferase II (KAT-II), and is responsible for the transamination of L-kynurenine to kynurenic acid (Buchli et al., 1995; Hallen et al., 2013). Alpha-AAA levels dictate the availability of KAT-II for the transamination of L-kynurenine to kynurenic acid (Schwarcz et al., 2012). Moreover, acute AMPH caused a shift in the ratio between glycine and serine in favor of the latter. Biosynthesis of glycine occurs through the conversion of L-serine to glycine by the enzyme serine hydroxyl-methyl-transferase (Appaji Rao et al., 2003). This shift shows that probably less glycine is formed from L-serine under the influence of acute AMPH. Glycine plays a role as an inhibitory (via glycine receptors) as well as excitatory (via NMDA receptors) neurotransmitter in the brain (Hernandes and Troncone, 2009). D-serine is formed from L-serine and it interacts with a D-serine/glycine modulatory site on the NR1subunit of NMDA receptor (Johnson and Ascher, 1987; Clements and Westbrook, 1991). The D-serine/glycine site on the NMDA receptor must be occupied for glutamate to activate the receptor (Clements and Westbrook, 1991).

As mentioned above, the acute response of 129Sv strain to AMPH-induced motor stimulation was apparently weaker and meanwhile these mice lost body weight. The reduced body weight was accompanied by lower levels of hexoses in the saline administration group of 129Sv (5,857 ± 1,146 μmoles) compared to the respective group of Bl6 (8,900 ± 1,195 μmoles). Therefore, one can conclude that 129Sv is in terms of hexoses (including glucose) in compromised status compared to Bl6 and, therefore, other sources of energy are needed. This is a likely reason why repeated AMPH caused in 129Sv a wider deviation of metabolite levels compared to Bl6 (Figure 2). Acute AMPH in 129Sv group, reduced the levels of various metabolites compared to saline, including valine, lysoPCs (lyso PC aa 16:0, lyso PC aa 18:2, lyso PC aa 20:4), PC diacyls (PC aa 34:2, PC aa 36:2, PC aa 36:3, PC aa 36:4) and PC acyl-alkyls (PC ae 38:4 and PC ae 40:4) (Table 2). Comparison of acute and repeated AMPH established that several metabolites were elevated due to repeated AMPH in 129Sv, including long chain acylcarnitines (C14, C14:1-OH, C16, C16:1, C18:1), BCAA (particularly isoleucine, valine), PC diacyls (PC aa C38:4, PC aa C38:6, PC aa C42:6), PC acyl-alkyls (PC ae C38:4, PC ae C40:4, PC ae C40:5, PC ae C40:6, PC ae C42:1, PC ae C42:3), and sphingolipids [SM(OH)C22:1, SM C24:0]. The elevation of the ratio between long chain acylcarnitines and carnitine (CPT-1 ratio) probably reflects the elevated participation of these metabolites due to need of additional energy for workload in 129Sv. Also, there were tendencies for a shift in the ratio between short chain acylcarnitines (C3, C5) and carnitine, favoring short chain acylcarnitines (Table 2, Supplementary Table S1). This probably reflects the metabolic value of elevated levels of BCAA (isoleucine, valine) in 129Sv repeated AMPH. These BCAAs are used for the synthesis of acylcarnitines C3 and C5.

### Distinct metabolomic response in subgroups of 129Sv

The distinct response of 129Sv to AMPH in the locomotor test was further analyzed at the metabolite level (Supplementary Table S2). Strong responders to AMPH displayed elevated levels of long chain acylcarnitines (C12, C14, C14:1-OH, C16:1) and reduced levels of hexoses compared to weak responders (Table 3). These metabolic changes can be taken as a compensatory response to the augmented workload in strong responders due to the stimulating effect of AMPH. Besides, the ratio between glycine and glutamine was reduced in strong responders, whereas the ratio between tyrosine and phenylalanine tended to be higher in strong responders. This may reflect the reduced availability of inhibitory transmitter glycine and increased availability of tyrosine as the precursor molecule of catecholamines in strong responders. Correlation analysis established a strong positive link between AMPH-induced motor stimulation and long-chain acylcarnitines C16 (ρ = 0.55) and C16:1 (ρ = 0.56), and with the ratio between tyrosine and phenylalanine (ρ = 0.64) (Supplementary Table S3). Negative correlation was established between locomotor activity and the ratio of glycine with histidine (ρ = −0.61). A strong negative correlation between C18:1 and hexoses demonstrated that animals with the lowest levels of hexoses displayed the highest levels of C18:1. This can be taken as a compensatory change to the limited amount of hexoses as the metabolic resource. On the other hand, C18:1 has been demonstrated as the pharmacologically active compound blocking the activity of glycine type 2 transporter (Carland et al., 2013). Inhibitory glycinergic neurotransmission is terminated by glycine transporters GlyT1 and GlyT2 which reuptake glycine from the synaptic cleft. GlyT2 is the principal supplier of glycine for vesicle refilling, a process that is necessary to preserve the amount of glycine in synaptic vesicles (Jiménez et al., 2015). In the multivariate regression modeling, the final parsimonious model retained distance traveled on day 11, C14:1, C16, C16:1, C18:1 and the ratio between glycine and glutamine as significant predictors of high motor response (Table 4). The whole model Wilk's lambda = 0.19, *F*_(6, 7)_ = 5.16, *p* = 0.03, multivariate partial η^2^ = 0.81, which indicates that ~81% of the multivariate variance of dependent variables is associated with the AMPH response division factor.

### Combining of metabolite data from 129Sv and Bl6

**Figure 6:**
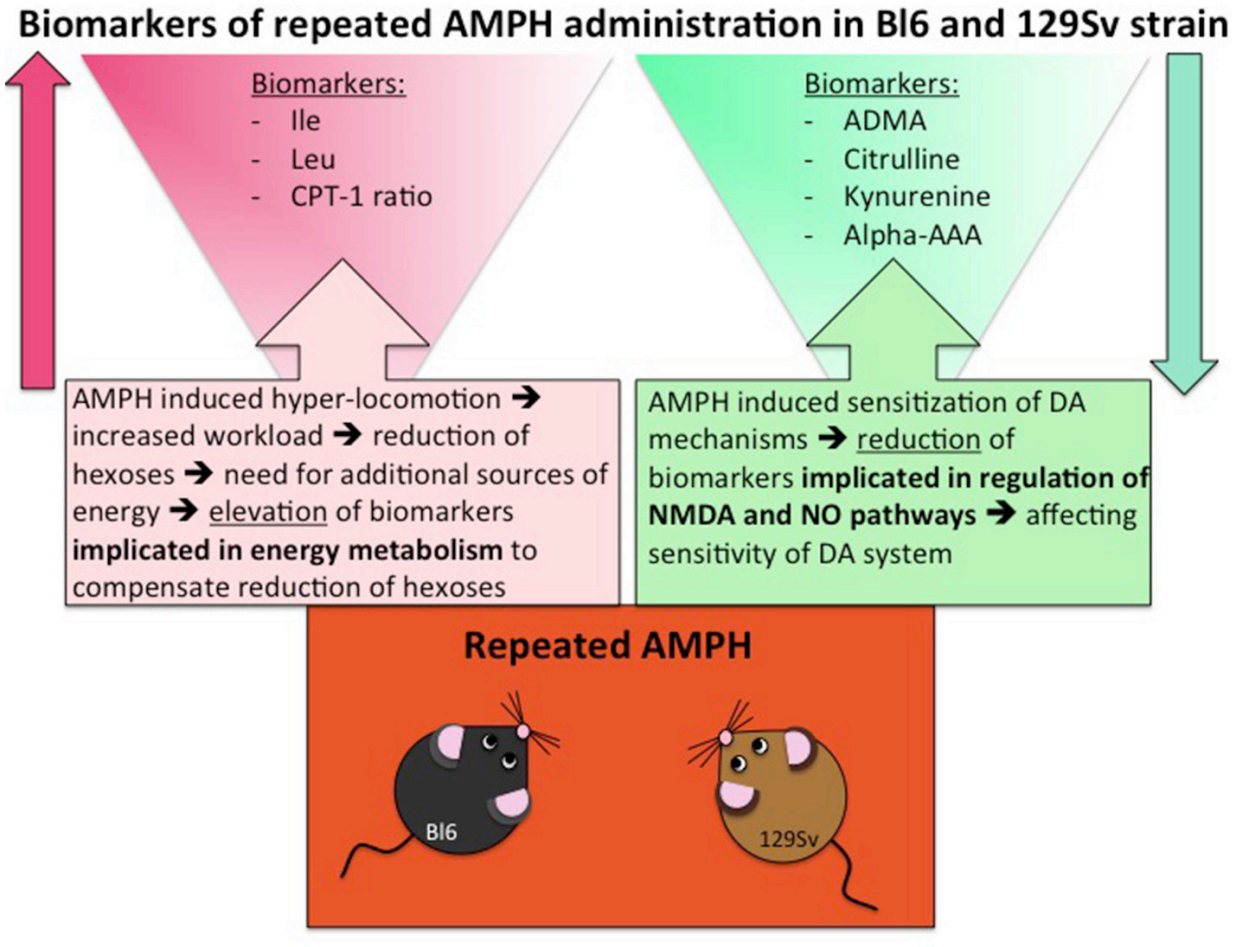
Biomarkers of repeated AMPH administration in Bl6 and 129Sv strain. Metabolites that are involved due to enhanced workload and metabolites that are implicated due to sensitization of DA system.

Despite significant basal differences between 129Sv and Bl6 strains, combining the data from these strains revealed that 14 metabolites or their ratios remained significant with repeated AMPH (Table 5, Supplementary Figure S4). The reduction of kynurenine displayed a large effect size (η^2^ = 0.15), whereas the other effects were moderate (η^2^ = 0.06–0.13). The levels of BCAA (leucine, isoleucine) and the ratio lysoPC a C20:4/lysoPC a C20:3 were markedly increased by repeated AMPH. By contrast, the levels of citrulline, biogenic amines (ADMA, kynurenine), hexoses and lysoPC a C18:2 were significantly reduced with repeated AMPH. Moreover, long chain acylcarnitines (CPT-1 ratio, C14) and PC alkyl-acyls (PC ae 40:6, PC ae 42:1) displayed an elevation if acute and repeated AMPH were compared. The elevation of BCAAs and lipid metabolites is probably associated with the reduction of hexoses, showing a need for the additional sources of energy for elevated workload (Figure 6). The reduction of citrulline, ADMA and kynurenine probably reflects alterations in NMDA and NO systems, inherent for the development of DA agonist-induced sensitization (Lang et al., 1995; Võikar et al., 1999; Chen et al., 2001; Liu et al., 2011).

## Conclusion

Amphetamine-induced behavioral sensitization is widely used behavioral paradigm in preclinical psychosis research, modeling increased DA signaling hypothesis. Metabolomic approach was taken to establish whether we are able to determine potential biomarkers of distinct AMPH-induced behavioral responses in Bl6 and 129Sv mice. These biomarkers may have potential value in terms of clinical research of first episode psychosis. Systematic comparison of human and animal data may provide useful hints for further clinical research. Indeed, the present study revealed substantial differences in the response of two commonly used mouse strains to acute and repeated AMPH. Acute AMPH caused significantly greater stimulation of motor activity in Bl6 mice. After repeated administration these differences between the strains vanished. However, the motor response of 129Sv mice to repeated AMPH displayed significantly larger variation than in Bl6 mice. Among 129Sv mice one can easily establish two groups: one responds similarly to the acute AMPH group, and the other displays robust sensitization toward the motor stimulating effect of AMPH. Aberrant response of 129Sv mice to experimental conditions, resulting in substantial body weight loss, may be associated with the mutation of *Disc1* gene in 129Sv. Studies in rats demonstrate that misassembly of full-length DISC1 protein alters DA homeostasis, leading to apparent behavioral deficits (Trossbach et al., 2016).

In the current study, the elevated levels of BCAAs, isoleucine and leucine, displayed the strongest relation to AMPH-induced motor sensitization. In Bl6 this elevation was apparent after acute AMPH, whereas in 129Sv a similar trend was observed after repeated AMPH. The pooled data from Bl6 and 129Sv mice demonstrated a clear overlap between sensitization toward AMPH-induced hyperlocomotion and elevated levels of leucine and isoleucine. However, we did not detect an elevation of byproducts of BCAA catabolism acylcarnitines C3 and C5, glutamine and alanine in response to repeated AMPH. Nevertheless, the ratio of acylcarnitines C3 and C5 with carnitine shifted in favor of short chain acylacarnitines in 129Sv, showing a possible role of BCAAs. It is apparent that isoleucine, leucine and valine are used for the synthesis of acylcarnitines C3 and C5. Moreover, isoleucine and leucine compete with tryptophan, phenylalanine, tyrosine and valine for transport into the mammalian cells by large neutral amino acid transporter (LAT1) (Fernstrom, 2005; Pochini et al., 2014). Nonetheless, the levels of these amino acids were not changed significantly. Human studies demonstrate that BCAAs and related metabolites are associated with insulin resistance and diabetes, predictive of diabetes development, and predictive of intervention outcomes (Newgard, 2012). Therefore, it is evident that isoleucine and leucine play a role in the metabolic homeostasis and their elevation can be taken as a compensatory response to the reduced levels of hexoses.

Largely deviated metabolite alterations established in 129Sv mice can also be linked to the reduced levels of hexoses. Apparently, the reduction of hexoses must be replaced by other sources of energy. Otherwise it would be impossible to keep such high motor activity response to AMPH as was established among strong responder group of 129Sv mice. Therefore, the increase of BCAA as well as the shift in the ratio between acylcarnitines (CPT-1, C3, C5) and carnitine in favor of former ones can be taken as a compensatory response in energy metabolism in 129Sv. Simultaneously with the elevation of BCAA, levels of kynurenine, citrulline and ADMA were reduced after repeated AMPH, showing probable alterations in NMDA and NO mediated mechanisms in mice (Figure 6). This is in line with the understanding that these mechanisms mediate DA agonist-induced motor sensitization (Lang et al., 1995; Võikar et al., 1999; Chen et al., 2001; Liu et al., 2011). After repeated AMPH the differences in motor response between the groups of Bl6 and 129Sv disappeared. Therefore, one has to underline that after repeated AMPH 129Sv displayed stronger motor sensitization compared to Bl6.

## Author contributions

All authors participated in the experiments of the study and review of the manuscript. EV, JI, TV, MP, MA-P, and JN designed the experiments. EV, MZ, LH, JN, and TV wrote the manuscript and analyzed the data. JN and TV made behavioral experiments. AO made FIA-MS/MS and LC-MS/MS measurements. All authors revised and approved the final manuscript.

### Conflict of interest statement

The authors declare that the research was conducted in the absence of any commercial or financial relationships that could be construed as a potential conflict of interest.
