# Supplementary material for: Repeated Administration of D-Amphetamine Induces Distinct Alterations in Behavior and Metabolite Levels in 129Sv and Bl6 Mouse Strains
Source: Front Neurosci. 2018 Jun 12;12:399. doi: 10.3389/fnins.2018.00399 (PMC6005828; doi:10.3389/fnins.2018.00399)
Supplement: Supplementary file 1 [file Data_Sheet_1.docx]

***Supplementary Material***

# Repeated Administration of D-amphetamine Induces Distinct Alterations in Behavior and Metabolite Levels of 129Sv and Bl6 Mouse Strains

Taavi Vanaveski^a,c^, Jane Narvik^a,c, *^, Jürgen Innos^a,c^, Mari-Anne Philips^a,c^, Aigar Ottas^b,c^, Mario Plaas^c,d^, Liina Haring^c,e^, Mihkel Zilmer^b,c^, Eero Vasar^a,c^

Correspondence:

Jane Narvik

jnarvik@ut.ee

Institute of Biomedicine and Translational Medicine, ^a^ Department of Physiology, ^b^ Department of Biochemistry, University of Tartu, Tartu, Estonia

^c^ Center of Excellence for Genomics and Translational Medicine, University of Tartu, Tartu, Estonia

^d^ Laboratory Animal Centre, Institute of Biomedicine and Translational Medicine, University of Tartu, Tartu, Estonia

^e^ Psychiatry Clinic, Tartu University Hospital, Tartu, Estonia

Supplementary material consists of three separate parts: (1) Figure S1 of behavioral and body weight data, Figures S2-S4 are conclusive schemas about metabolic outcomes, (2) raw metabolic data in Tables S1-S3 and (3) quality control data in Table S4.

# Supplementary Figure

## Supplementary Figure S1


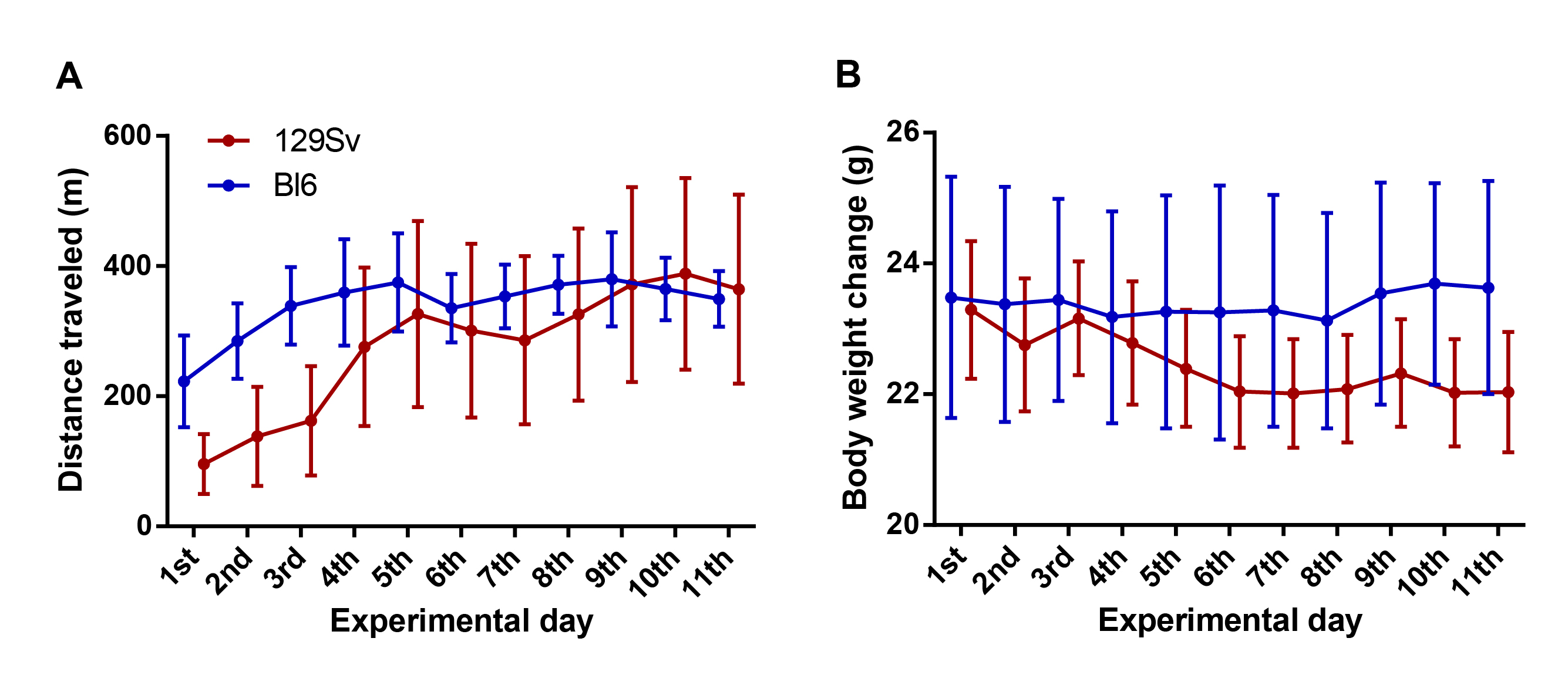


Time course of behavioral effects and body weight change in 129Sv and Bl6 during repeated AMPH administration (mean and 95% confidence interval). Locomotor activity **(A)**, body weight change **(B)**, during experimental period.

**Supplementary Figure S2**


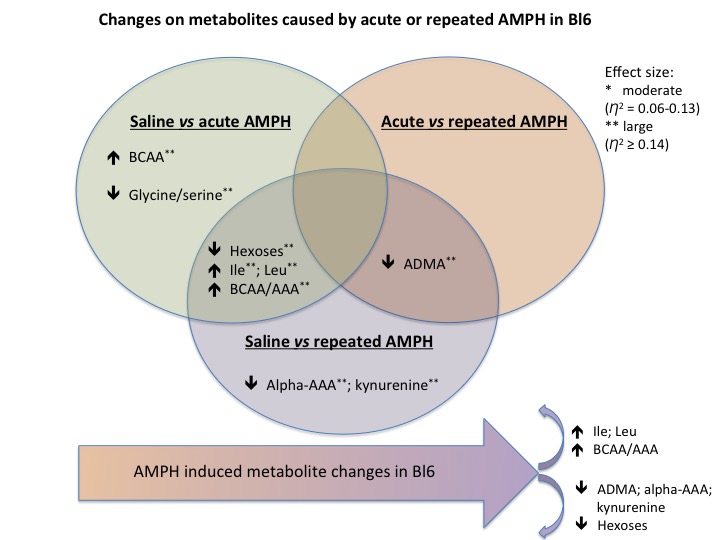


Changes on metabolites caused by acute or repeated AMPH in Bl6. Conclusive schema is based on the results from Table 1.

**Supplementary Figure S3**


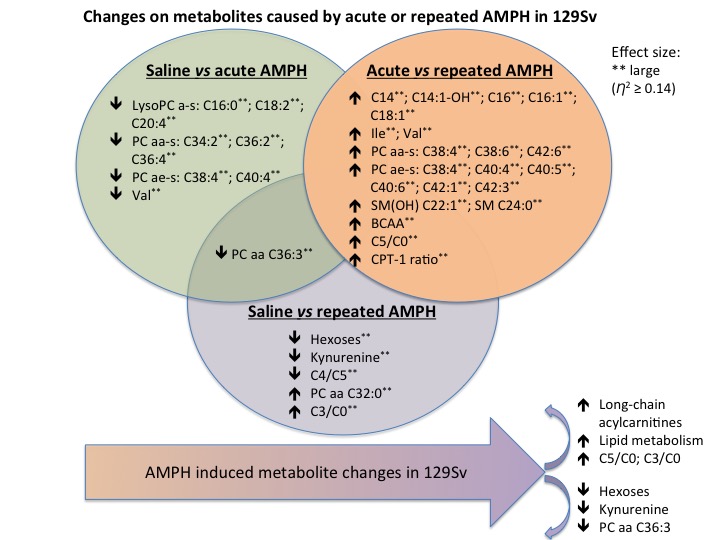


Changes on metabolites caused by acute or repeated AMPH in 129Sv. Conclusive schema is based on the results from Table 2. **Supplementary Figure S4**


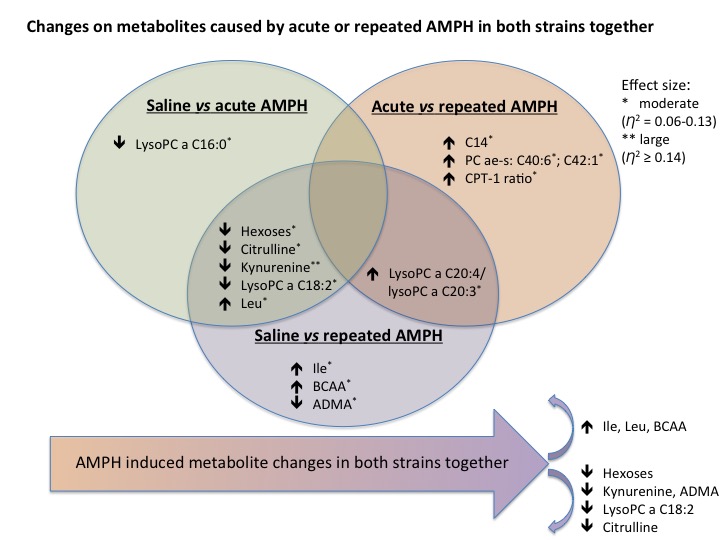


Changes on metabolites caused by acute or repeated AMPH in both strains togeather. Conclusive schema is based on the results from Table 5.

# 1. Metabolic Raw Data

## **Supplementary Table S1.**

D-amphetamine-induced changes in metabolites levels (µmoles, median and range) and their ratios in Bl6 and 129Sv. CPT1 (carnitine palmitoyltransferase 1) ratio [(C16 + C18) / carnitine]. * - saline administrations induced a statistically significant (p ≤0.05) difference in metabolite levels in Bl6 compared to 129Sv. Glycerophospholipids include: lysophosphatidylcholine acyls, phosphatidylcholine diacyls, and phosphatidylcholine acyl-alkyls (indicated in italic).

| **Biomarker** | **Bl6** | | | | | **129Sv** | | | | | | | | | | |
| --- | --- | --- | --- | --- | --- | --- | --- | --- | --- | --- | --- | --- | --- | --- | --- | --- |
|  | **Saline**  (N=12) | **Acute AMPH**  (N=16) | **Repeated AMPH**  (N=12) | Kruskal-  Wallis  test | | **Saline**  (N=11) | **Acute AMPH**  (N=14) | | | | **Repeated AMPH**  (N=14) | | | Kruskal-  Wallis  test | | |
| **Acylcarnitines** | | | | | | | | | | | | | | | | |
| C0 | 43.6  21.6 - 62.5 | 41.2  13.0 - 73.8 | 40.9  21.4 - 68.5 | | χ^2^_(2,40)_=0.63,  p=0.73 | 25.9  15.5 - 54 | | 40.7  16.8 - 132 | | | 27.8  14.1 - 50.0 | | | | χ^2^_(2,39)_=5.00,  p=0.08 | |
| C10 | 0.17  0.000 - 0.19 | 0.15  0.000 - 0.21 | 0.17  0.000 - 0.23 | | χ^2^_(2,40)_=1.38,  p=0.50 | 0.000  0.000 - 0.16 | | 0.000  0.000 - 0.21 | | | 0.000  0.000 - 0.20 | | | | χ^2^_(2,39)_=1.41,  p=0.50 | |
| C12 | 0.092  0.000 - 0.142 | 0.093  0.000 - 0.1219 | 0.085  0.000 - 0.18 | | χ^2^_(2,40)_=2.32,  p=0.31 | 0.094  0.000 - 0.12 | | 0.10  0.000 - 0.14 | | | 0.11  0.000 - 0.16 | | | | χ^2^_(2,39)_=2.23,  p=0.33 | |
| C14 | 0.091  0.060 - 0.13 | 0.075  0.054 - 0.14 | 0.080  0.059 -0.18 | | χ^2^_(2,40)_=1.12,  p=0.57 | 0.081  0.067 - 0.10 | | 0.071  0.054 - 0.13 | | | 0.103  0.069 - 0.16 | | | | χ^2^_(2,39)_=9.71,  **p=0.008** | |
| C14:1 | 0.063  0.039 - 0.082 | 0.055  0.034 - 0.097 | 0.060  0.039 - 0.11 | | χ^2^_(2,40)_=1.05,  p=0.59 | 0.057  0.036 - 0.073 | | 0.046  0.036 - 0.080 | | | 0.063  0.040 - 0.089 | | | | χ^2^_(2,39)_=6.22,  p=0.05 | |
| C14:1-OH | 0.016  0.000 - 0.020 | 0.014  0.000 - 0.022 | 0.015  0.000 - 0.024 | | χ^2^_(2,40)_=0.27,  p=0.87 | 0.013  0.000 - 0.019 | | 0.000  0.000 - 0.020 | | | 0.016  0.000 - 0.025 | | | | χ^2^_(2,39)_=11.2,  **p=0.004** | |
| C14:2 | 0.021  0.000 - 0.026 | 0.018  0.000 - 0.023 | 0.018  0.000 - 0.027 | | χ^2^_(2,40)_=1.81,  p=0.40 | 0.015  0.000 - 0.021 | | 0.016  0.000 - 0.030 | | | 0.018  0.014 - 0.024 | | | | χ^2^_(2,39)_=2.84,  p=0.24 | |
| C16 | 0.34  0.18 - 0.40 | 0.29  0.16 - 0.60 | 0.29  0.16 - 0.38 | | χ^2^_(2,40)_=1.55,  p=0.46 | 0.27  0.21 - 0.34 | | 0.24  0.17 - 0.38 | | | 0.31  0.23 - 0.41 | | | | χ^2^_(2,38)_= 11.25,  **p=0.004** | |
| C16-0H | 0.026  0.000 - 0.037 | 0.025  0.000 - 0.043 | 0.025  0.000 - 0.034 | | χ^2^_(2,40)_=0.66,  p=0.72 | 0.022  0.000 - 0.026 | | 0.021  0.000 - 0.048 | | | 0.026  0.020 - 0.041 | | | | χ^2^_(2,39)_=5.36,  p=0.07 | |
| C16:1 | 0.12  0.063 - 0.14 | 0.090  0.067 - 0.21 | 0.10  0.060 - 0.17 | | χ^2^_(2,40)_=0.79,  p=0.67 | 0.078  0.059 - 0.098 | | 0.068  0.039 - 0.12 | | | 0.096  0.067 - 0.14 | | | | χ^2^_(2,38)_=7.86,  **p=0.02** | |
| C16:1-OH | 0.019  0.000 - 0.027 | 0.016  0.000 - 0.027 | 0.016  0.000 - 0.024 | | χ^2^_(2,40)_=1.69,  p=0.43 | 0.015  0.000 - 0.019 | | 0.015  0.000 - 0.032 | | | 0.019  0.000 - 0.035 | | | | χ^2^_(2,39)_=5.08,  p=0.08 | |
| C18 | 0.061  0.034 - 0.072 | 0.054  0.027 - 0.11 | 0.054  0.037 - 0.071 | | χ^2^_(2,40)_=1.05,  p=0.59 | 0.053  0.049 - 0.077 | | 0.054  0.037 - 0.16 | | | 0.068  0.048 - 0.10 | | | | χ^2^_(2,39)_=2.88,  p=0.24 | |
| C18:1 | 0.26  0.15 - 0.36 | 0.25  0.11 - 0.62 | 0.25  0.12 - 0.43 | | χ^2^_(2,40)_=0.21,  p=0.90 | 0.15*  0.14 - 0.20 | | 0.14  0.11 - 0.26 | | | 0.20  0.13 - 0.23 | | | | χ^2^_(2,38)_=9.94,  **p=0.007** | |
| C18:2 | 0.088  0.051 - 0.12 | 0.073  0.040 - 0.20 | 0.071  0.037 - 0.10 | | χ^2^_(2,40)_=1.87,  p=0.39 | 0.063  0.055 - 0.091 | | 0.065  0.043 - 0.19 | | | 0.078  0.048 - 0.12 | | | | χ^2^_(2,39)_=3.25,  p=0.20 | |
| C2 | 34.2  28.3 - 45.6 | 33.5  21.9 - 48.7 | 33.5  22.5 - 60.7 | | χ^2^_(2,40)_=0.15,  p=0.93 | 32.1  28.5 - 38.8 | | 35.9  27.7 - 54.6 | | | 37.4  29.2 - 59.3 | | | | χ^2^_(2,39)_=4.26,  p=0.12 | |
| C3 | 1.07  0.58 - 1.45 | 0.94  0.39 - 1.77 | 0.83  0.46 - 1.80 | | χ^2^_(2,40)_=1.36,  p=0.51 | 0.71  0.35 - 1.58 | | 1.12  0.40 - 8.36 | | | 0.82  0.54 - 1.84 | | | | χ^2^_(2,39)_=3.74,  p=0.15 | |
| C4 | 0.89  0.61 - 1.22 | 0.85  0.41 - 1.28 | 0.76  0.59 - 2.46 | | χ^2^_(2,40)_=1.54,  p=0.46 | 1.29  0.67- 2.19 | | 1.42  0.72 - 2.38 | | | 1.11  0.81 - 2.25 | | | | χ^2^_(2,39)_=1.02,  p=0.60 | |
| C3-DC (C4- OH) | 0.35  0.21 - 0.57 | 0.36  0.17 - 0.62 | 0.33  0.20 - 1.13 | | χ^2^_(2,40)_=0.28,  p=0.87 | 0.30  0.17 - 0.40 | | 0.45  0.16 - 1.18 | | | 0.37  0.19 - 0.85 | | | | χ^2^_(2,39)_=5.76,  p=0.06 | |
| C5 | 0.22  0.18 - 0.28 | 0.19  0.15 - 0.33 | 0.21  0.13 - 0.35 | | χ^2^_(2,40)_=1.61,  p=0.44 | 0.40*  0.23 - 0.63 | | 0.51  0.23 - 1.68 | | | 0.48  0.33 - 1.03 | | | | χ^2^_(2,39)_=1.27,  p=0.53 | |
| C5-OH (C3-DC-M) | 0.15  0.000 - 0.18 | 0.15  0.000 - 0.23 | 0.13  0.000 - 0.28 | χ^2^_(2,40)_=2.85,  p=0.24 | | 0.14  0.000 - 0.22 | | 0.22  0.000 - 0.40 | | | 0.15  0.12 - 0.27 | | | | χ^2^_(2,39)_=6.86,  **p=0.03** | |
| C6 (C4:1-DC) | 0.064  0.044 - 0.099 | 0.059  0.032 - 0.088 | 0.057  0.041 - 0.13 | χ^2^_(2,40)_=1.34,  p=0.51 | | 0.058  0.037 - 0.085 | | 0.047  0.000 - 0.076 | | | 0.063  0.041 - 0.076 | | | | χ^2^_(2,39)_=3.15,  p=0.21 | |
| C5-DC (C6-OH) | 0.036  0.000 - 0.044 | 0.035  0.000 - 0.053 | 0.029  0.000 - 0.053 | χ^2^_(2,40)_=2.44,  p=0.30 | | 0.034  0.000 - 0.042 | | 0.041  0.000 - 0.094 | | | 0.038  0.000 - 0.073 | | | | χ^2^_(2,39)_=3.26,  p=0.20 | |
| C7-DC | 0.014  0.000 - 0.074 | 0.028  0.000 - 0.050 | 0.000  0.000 - 0.042 | χ^2^_(2,40)_=0.72,  p=0.70 | | 0.000  0.000 - 0.047 | | 0.000  0.000 - 0.066 | | | 0.000  0.000 - 0.072 | | | | χ^2^_(2,39)_=0.36,  p=0.84 | |
| C8 | 0.12  0.000 - 0.20 | 0.11  0.000 - 0.20 | 0.10  0.000 - 0.14 | χ^2^_(2,40)_=1.92,  p=0.38 | | 0.000  0.000 - 0.14 | | 0.000  0.000 - 0.16 | | | 0.000  0.000 - 0.13 | | | | χ^2^_(2,39)_=1.00,  p=0.61 | |
| **Monosaccharides** | | | | | | | | | | | | | | | | |
| Hexoses | 8569  7452 - 11103 | 7407  4459 - 9751 | 7343  3810 - 9625 | χ^2^_(2,39)_=6.34,  **p=0.04** | | 5810*  4405 - 8005 | | | 4299  3269 - 6617 | | 4549  2764 - 5937 | | | | χ^2^_(2,38)_=8.21,  **p=0.02** | |
| **Amino acids** | | | | | | | | | | | | | | | | |
| Alanine | 527  344 - 991 | 572  221 - 874 | 472  267 - 1030 | χ^2^_(2,40)_=1.25,  p=0.54 | | 410  296 - 677 | | 463  217 - 1330 | | | 399  221 - 643 | | | | χ^2^_(2,39)_=1.69,  p=0.43 | |
| Arginine | 70.6  3.70 - 146 | 84.4  5.40 - 359 | 68.9  3.40 - 260 | χ^2^_(2,40)_=0.90,  p=0.64 | | 31.7  2.79 - 102 | | 25.9  2.83 - 208 | | | 58.8  3.47 - 414 | | | | χ^2^_(2,39)_=1.58,  p=0.45 | |
| Asparagine | 20.5  16.9 - 41.5 | 24.0  8.20 - 115 | 19.0  9.30 - 61.9 | χ^2^_(2,40)_=0.70,  p=0.71 | | 25.9  19.6 - 38.8 | | 21.1  12.1 - 35.3 | | | 21.4  13.3 - 62.7 | | | | χ^2^_(2,39)_=1.55,  p=0.46 | |
| Aspartate | 83.2  19.3 - 266 | 86.0  16.5 - 191 | 76.0  0.000 - 186 | χ^2^_(2,40)_=0.85,  p=0.65 | | 86.9  15.2 - 182 | | 66.1  15.1 - 295 | | | 48.5  22.8 - 218 | | | | χ^2^_(2,39)_=2.52,  p=0.28 | |
| Citrulline | 52.1  40.0 - 112 | 47.0  19.9 - 60.1 | 43.3  29.0 – 55.0 | χ^2^_(2,39)_=4.24,  p=0.12 | | 49.5  28.9 - 111 | | 37.3  24.0 – 52.1 | | | 33.3  27.5 - 73.2 | | | | χ^2^_(2,38)_=5.85,  p=0.05 | |
| Glutamine | 853  509 - 1120 | 755  467 - 1190 | 862  501 - 1130 | χ^2^_(2,40)_=1.88,  p=0.39 | | 727  605 - 1090 | | 700  531 - 1630 | | | 677  471 - 979 | | | | χ^2^_(2,39)_=2.25,  p=0.33 | |
| Glutamate | 1340  283 - 3760 | 1095  56 - 6910 | 997  128 - 5510 | χ^2^_(2,40)_=1.10,  p=0.58 | | 519  77.9 - 1900 | | 850  75.1 - 6350 | | | 576  95.8 - 4080 | | | | χ^2^_(2,39)_=3.00,  p=0.22 | |
| Glycine | 544  184 - 772 | 455  144 - 728 | 463  197 - 1870 | χ^2^_(2,40)_=2.72,  p=0.26 | | 322*  180 - 467 | | 245  149 - 972 | | | 342  212 - 581 | | | | χ^2^_(2,39)_=3.43,  p=0.18 | |
| Histidine | 70.6  44.4 - 81.9 | 83.9  36.3 - 117.0 | 67.9  47.8 - 107.0 | χ^2^_(2,40)_=2.31,  p=0.32 | | 70.1  55.7 - 120 | | 58.9  42.0 - 147 | | | 68.4  59.2 - 130 | | | | χ^2^_(2,39)_=4.60,  p=0.10 | |
| Isoleucine | 83.7  60.6 - 108 | 105  75.9 - 214 | 104  62.5 - 208 | χ^2^_(2,40)_=8.98,  **p=0.01** | | 97.3  79.8 - 129 | | 92.6  75.6 - 120 | | | 121  69.4 - 203 | | | | χ^2^_(2,38)_=6.92,  **p=0.03** | |
| Leucine | 123  92.8 - 159 | 158  112 - 372 | 160  104 - 361 | χ^2^_(2,39)_=10.87,  **p=0.004** | | 137  111 - 197 | | 147  107 - 287 | | | 169  107 - 413 | | | | χ^2^_(2,39)_=4.82,  p=0.09 | |
| Lysine | 267  159 - 397 | 314  103 - 766 | 261  133 - 500 | χ^2^_(2,40)_=1.50,  p=0.47 | | 240  140 - 399 | | 170  95.7 - 617 | | | 198  105 - 721 | | | | χ^2^_(2,39)_=0.80,  p=0.67 | |
| Methionine | 38.6  30.0 - 56.7 | 45.4  23.4 - 102 | 34.1  15.2 - 120 | χ^2^_(2,40)_=5.39,  p=0.07 | | 41.9  28.9 - 73.6 | | 38.3  27.7 - 93.1 | | | 36.9  25.8 - 161 | | | | χ^2^_(2,39)_=1.58,  p=0.45 | |
| Ornithine | 77.3  24.1 - 146 | 82.5  13.2 - 269 | 59.3  2.52 - 157 | χ^2^_(2,40)_=0.49,  p=0.78 | | 77.5  20.2 - 188 | | 53.2  12.6 - 351 | | | 34.8  11.3 - 152 | | | | χ^2^_(2,39)_=3.27,  p=0.20 | |
| Phenylalanine | 82.2  54.5 - 95.5 | 95.8  56.9 - 210 | 83.9  53.8 - 196 | χ^2^_(2,40)_=2.19,  p=0.34 | | 74.5  52.1 - 94.5 | | 71.7  50.7 - 128 | | | 75.0  56.8 - 206 | | | | χ^2^_(2,39)_=1.55,  p=0.46 | |
| Proline | 85.6  54.3 - 138 | 96.3  30.4 - 227 | 75.6  43.2 - 197 | χ^2^_(2,40)_=1.93,  p=0.38 | | 71.8  45.7 - 116 | | 63.4  39.3 - 167 | | | 66.1  47.3 - 210 | | | | χ^2^_(2,39)_=0.02,  p=0.99 | |
| Serine | 149  89.2 - 248 | 198  59.9 - 337 | 135  21.4 - 309 | χ^2^_(2,40)_=4.05,  p=0.13 | | 145  90.2 - 238 | | 146  78.9 - 371 | | | 168  92.0 - 262 | | | | χ^2^_(2,39)_=1.07,  p=0.59 | |
| Threonine | 135  89.8 - 169 | 124  63.0 - 190 | 106  10.4 - 219 | χ^2^_(2,40)_=1.43,  p=0.49 | | 134  101 - 233 | | 122  70.9 - 193 | | | 119  74.7 - 189 | | | | χ^2^_(2,39)_=2.74,  p=0.26 | |
| Tryptophan | 73.4  56.3 - 114 | 70.6  55.9 - 105 | 68.9  56.4 - 128 | χ^2^_(2,40)_=0.35,  p=0.84 | | 68.7  52.2 - 95.3 | | 59.7  36.8 - 114 | | | 64.1  51.5 - 98.8 | | | | χ^2^_(2,39)_=0.57,  p=0.75 | |
| Tyrosine | 56.9  41.0 - 74.2 | 64.3  41.3 - 152 | 57.8  39.3 - 152 | χ^2^_(2,40)_=0.74,  p=0.69 | | 46.6  27.4 - 56.5 | | 39.4  30.4 - 82.2 | | | 49.4  33.4 - 135 | | | | χ^2^_(2,39)_=4.54,  p=0.10 | |
| Valine | 160  137 - 208 | 180  118 - 299 | 179  131 - 370 | χ^2^_(2,40)_=2.39,  p=0.30 | | 192  146 - 297 | | 149  117 - 232 | | | 198  115 - 275 | | | χ^2^_(2,38)_=7.88,  **p=0.02** | | |
| **Biogenic amines** | | | | | | | | | | | | | | | | |
| Ac-Orn | 15.9  10.6 - 19.1 | 13.9  7.22 - 24.5 | 12.7  10.2 - 17.8 | χ^2^_(2,40)_=4.70,  p=0.10 | | 7.25*  5.40 - 12.1 | | 8.51  5.84 - 14.5 | | | 7.67  5.00 - 13.7 | | | χ^2^_(2,39)_=1.20,  p=0.55 | | |
| ADMA | 0.36  0.21 - 0.75 | 0.40  0.24 - 0.80 | 0.23  0.000 - 0.60 | χ^2^_(2,37)_=8.75,  **p=0.01** | | 0.28  0.11 - 0.53 | | 0.25  0.000 - 1.27 | | | 0.26  0.000 - 0.52 | | | χ^2^_(2,39)_=1.42,  p=0.49 | | |
| alpha-AAA | 11.0  7.42 - 17.2 | 10.2  0.000 - 16.0 | 8.63  4.46 - 10.3 | χ^2^_(2,36)_=7.66,  **p=0.02** | | 0.000*  0.000 - 9.49 | | 0.000  0.000 - 18.2 | | | 0.000  0.000 - 6.43 | | | χ^2^_(2,39)_=2.14,  p=0.34 | | |
| Carnosine | 15.6  3.20 - 21.2 | 13.5  2.01 - 26.8 | 9.12  2.66 - 38.0 | χ^2^_(2,40)_=1.99,  p=0.37 | | 2.79*  1.17 - 7.34 | | 2.58  1.16 - 11.6 | | | 2.96  1.29 - 7.52 | | | χ^2^_(2,39)_=0.39,  p=0.82 | | |
| Creatinine | 16.6  7.80 - 25.6 | 17.4  5.09 - 35.6 | 12.3  7.17 - 32.1 | χ^2^_(2,40)_=2.69,  p=0.26 | | 10.2*  5.11 - 19.5 | | 13.3  7.07 - 40.8 | | | 11.7  6.11 - 18.2 | | | χ^2^_(2,39)_=0.69,  p=0.71 | | |
| Histamine | 2.42  0.31 - 4.03 | 2.98  1.36 - 9.95 | 2.74  1.29 - 4.04 | χ^2^_(2,40)_=2.25,  p=0.33 | | 2.60  1.12 - 7.01 | | 3.58  0.87 - 5.96 | | | 3.73  1.69 - 6.66 | | | χ^2^_(2,39)_=1.74,  p=0.42 | | |
| Kynurenine | 1.40  0.93 - 1.56 | 1.17  0.89 - 1.58 | 1.07  0.83 - 1.90 | χ^2^_(2,38)_=7.10,  **p=0.03** | | 1.43  1.13 - 1.90 | | 1.24  0.941 - 1.64 | | | 1.27  1.05 - 1.45 | | | χ^2^_(2,37)_=6.10,  **p=0.04** | | |
| Met-SO | 0.000  0.000 - 1.61 | 0.000  0.000 - 1.14 | 0.000  0.000 - 2.72 | χ^2^_(2,40)_=0.34,  p=0.84 | | 0.000  0.000 - 2.05 | | 0.000  0.000 - 1.19 | | | 0.000  0.000 - 0.000 | | | χ^2^_(2,39)_=4.95,  p=0.08 | | |
| PEA | 0.072  0.000 - 0.10 | 0.072  0.000 - 0.092 | 0.075  0.070 - 1.47 | χ^2^_(2,40)_=4.14,  p=0.13 | | 0.072  0.069 - 0.087 | | 0.075  0.000 - 0.13 | | | 0.073  0.069 - 0.11 | | | χ^2^_(2,39)_=0.37,  p=0.83 | | |
| Putrescine | 1.15  0.000 - 1.84 | 1.42  0.49 - 4.47 | 1.35  0.68 - 3.27 | χ^2^_(2,40)_=1.99,  p=0.37 | | 0.59  0.31 - 1.15 | | 0.66  0.34 - 2.67 | | | 0.69  0.37 - 1.45 | | | χ^2^_(2,39)_=0.54,  p=0.76 | | |
| Taurine | 885  723 - 1040 | 881  484 - 1040 | 843  608 - 1070 | χ^2^_(2,40)_=0.76,  p=0.69 | | 819  451 - 1000 | | 924  549 - 1070 | | | 856  585 - 1020 | | | χ^2^_(2,39)_=2.05,  p=0.36 | | |
| 5-HT | 11.5  6.28 - 25.9 | 11.2  3.90 - 19.6 | 14.0  7.59 - 36.3 | χ^2^_(2,40)_=2.71,  p=0.26 | | 5.91  2.50 - 18.8 | | 5.87  4.51 - 15.2 | | | 7.36  2.55 - 14.9 | | | χ^2^_(2,39)_=0.19,  p=0.91 | | |
| Spermidine | 15.1  4.62 - 40.6 | 17.1  5.49 - 25.2 | 13.3  5.63 - 82.9 | χ^2^_(2,40)_=0.80,  p=0.67 | | 9.15  4.61 - 18.1 | | 12.1  2.40 - 49.8 | | | 12.2  4.23 - 32.6 | | | χ^2^_(2,39)_=2.21,  p=0.33 | | |
| Spermine | 5.54  2.35 - 14.8 | 7.84  2.16 - 11.5 | 5.40  1.21 - 14.8 | χ^2^_(2,40)_=2.00,  p=0.37 | | 4.73  1.38 - 10.4 | | 7.98  0.91 - 26.0 | | | 6.04  2.37 - 17.0 | | | χ^2^_(2,39)_=3.97,  p=0.14 | | |
| t4-OH-Pro | 23.6  7.54 - 38.5 | 22.5  4.39 - 41.1 | 19.7  6.30 - 23.9 | χ^2^_(2,40)_=3.20,  p=0.20 | | 13.3  5.80 - 21.3 | | 10.3  7.49 - 17.0 | | | 11.2  5.24 - 24.5 | | | χ^2^_(2,39)_=2.38,  p=0.30 | | |
| **Glycerophospholipids** | | | | | | | | | | | | | | | | |
| ***Lysophosphatidylcholine acyls*** | | | | | | | | | | | | | | | | |
| lysoPC a  C16:0 | 312  158 - 376 | 260  127 - 411 | 270  127 - 443 | χ^2^_(2,40)_=1.02,  p=0.60 | | 292  179 - 444 | | 211  143 - 366 | | | 268  121 - 333 | | | χ^2^_(2,39)_=6.81,  **p=0.03** | | |
| lysoPC a  C16:1 | 12.8  6.31 - 17.8 | 11.0  6.06 - 19.1 | 14.1  6.86 - 20.3 | χ^2^_(2,40)_=1.58,  p=0.45 | | 5.87*  3.50 - 8.26 | | 4.47  3.01 - 8.90 | | | 4.67  2.83 - 6.91 | | | χ^2^_(2,39)_=4.25,  p=0.12 | | |
| lysoPC a  C17:0 | 3.52  1.89 - 4.64 | 3.14  1.64 - 4.26 | 3.31  1.55 - 5.34 | χ^2^_(2,40)_=2.11,  p=0.35 | | 4.58  3.24 - 6.56 | | 3.48  2.62 - 5.69 | | | 4.52  2.07 - 5.91 | | | χ^2^_(2,39)_=4.16,  p=0.13 | | |
| lysoPC a  C18:0 | 98.4  47.9 - 114 | 82.5  33.3 - 134 | 89.6  40.7 - 160 | χ^2^_(2,40)_=0.97,  p=0.62 | | 105  63 - 173 | | 78.5  62.1 - 130 | | | 101  42.5 - 144 | | | χ^2^_(2,39)_=4.84,  p=0.09 | | |
| lysoPC a  C18:1 | 81.1  39.5 - 110 | 71.5  39.3 - 122 | 91.5  46.1 - 149 | χ^2^_(2,40)_=1.66,  p=0.44 | | 59.8  34.8 - 86.1 | | 45.7  28.7 - 78.9 | | | 47.0  31.3 - 66.0 | | | χ^2^_(2,39)_=4.66,  p=0.10 | | |
| lysoPC a  C18:2 | 160  98.7 - 198 | 142  85.1 - 242 | 140  92.0 - 265 | χ^2^_(2,40)_=1.39,  p=0.50 | | 133  88.0 - 181 | | 90.1  61.1 - 169 | | | 107  56.3 - 122 | | | χ^2^_(2,39)_=9.09,  **p=0.01** | | |
| lysoPC a  C20:3 | 12.5  6.40 - 17.7 | 11.9  5.54 - 19.7 | 13.6  7.30 - 23.5 | χ^2^_(2,40)_=0.63,  p=0.73 | | 8.94  4.98 - 13.8 | | 6.43  4.04 - 10.5 | | | 6.91  3.97 - 8.57 | | | χ^2^_(2,39)_=5.16,  p=0.08 | | |
| lysoPC a  C20:4 | 49.4  20.0 - 59.2 | 40.3  14.7 - 73.6 | 44.4  21.2 - 83.2 | χ^2^_(2,40)_=1.26,  p=0.53 | | 29.3  20.2 - 44.0 | | 20.6  14.8 - 34.9 | | | 28.7  16.4 - 34.7 | | | χ^2^_(2,39)_=7.57,  **p=0.02** | | |
| lysoPC a  C24:0 | 0.92  0.44 - 1.10 | 0.97  0.36 - 1.26 | 0.87  0.56 - 1.85 | χ^2^_(2,40)_=0.36,  p=0.84 | | 0.95  0.56 - 1.47 | | 0.96  0.50 - 1.48 | | | 1.06  0.71 - 1.37 | | | χ^2^_(2,39)_=1.74,  p=0.42 | | |
| lysoPC a  C26:0 | 1.11  0.43 - 1.46 | 1.06  0.36 - 1.76 | 1.01  0.63 - 2.84 | χ^2^_(2,40)_=0.43,  p=0.81 | | 0.92  0.36 - 1.68 | | 0.88  0.41 - 2.26 | | | 0.91  0.55 - 1.34 | | | χ^2^_(2,39)_=0.16,  p=0.92 | | |
| lysoPC a  C26:1 | 0.36  0.16 - 0.57 | 0.40  0.061 - 0.62 | 0.44  0.26 - 1.01 | χ^2^_(2,40)_=1.31,  p=0.52 | | 0.30  0.15 - 0.69 | | 0.30  0.11 - 0.55 | | | 0.37  0.25 - 0.60 | | | χ^2^_(2,39)_=3.39,  p=0.18 | | |
| lysoPC a  C28:0 | 0.54  0.27 - 0.73 | 0.61  0.18 - 0.79 | 0.66  0.36 - 1.53 | χ^2^_(2,40)_=2.80,  p=0.25 | | 0.49  0.18 - 0.91 | | 0.50  0.27 - 1.27 | | | 0.53  0.40 - 0.94 | | | χ^2^_(2,39)_=1.31,  p=0.52 | | |
| lysoPC a  C28:1 | 0.47  0.19 - 0.59 | 0.44  0.16 - 0.74 | 0.40  0.25 - 1.22 | χ^2^_(2,40)_=0.11,  p=0.95 | | 0.29  0.11 - 0.73 | | 0.36  0.15 - 0.71 | | | 0.35  0.23 - 0.75 | | | χ^2^_(2,39)_=1.28,  p=0.53 | | |
| ***Phosphatidylcholine diacyls*** | | | | | | | | | | | | | | | | |
| PC aa C24:0 | 0.42  0.16 - 0.70 | 0.40  0.11 - 0.76 | 0.42  0.24 - 1.01 | χ^2^_(2,40)_=1.38,  p=0.50 | | 0.29  0.18 - 0.52 | | 0.28  0.14 - 0.58 | | | 0.37  0.21 - 0.58 | | | χ^2^_(2,39)_=2.74,  p=0.26 | | |
| PC aa C26:0 | 1.77  0.000 - 2.68 | 1.59  0.000 - 2.48 | 1.54  0.00 - 4.49 | χ^2^_(2,40)_=0.28,  p=0.87 | | 1.46  0.000 - 2.41 | | 1.45  0.000 - 2.71 | | | 1.56  0.000 - 2.02 | | | χ^2^_(2,39)_=0.31,  p=0.86 | | |
| PC aa C28:1 | 0.48  0.19 - 0.69 | 0.42  0.18 - 0.73 | 0.42  0.26 - 1.44 | χ^2^_(2,40)_=0.26,  p=0.88 | | 0.41  0.20 - 0.66 | | 0.36  0.16 - 0.64 | | | 0.41  0.25 - 0.57 | | | χ^2^_(2,39)_=0.43,  p=0.81 | | |
| PC aa C30:0 | 1.00  0.53 - 2.04 | 0.91  0.51 - 2.11 | 0.99  0.58 - 1.13 | χ^2^_(2,40)_=1.06,  p=0.59 | | 0.86  0.53 - 1.41 | | 0.91  0.63 - 3.39 | | | 0.99  0.71 - 2.74 | | | χ^2^_(2,39)_=5.25,  p=0.07 | | |
| PC aa C30:2 | 0.078  0.028 - 0.17 | 0.089  0.000 - 0.29 | 0.069  0.000 - 0.38 | χ^2^_(2,40)_=0.73,  p=0.69 | | 0.070  0.019 - 0.16 | | 0.055  0.000 - 0.18 | | | 0.053  0.000 - 0.15 | | | χ^2^_(2,39)_=2.69,  p=0.26 | | |
| PC aa C32:0 | 12.0  6.95 - 22.1 | 11.0  6.39 - 23.2 | 10.1  6.82 - 14.0 | χ^2^_(2,40)_=2.55,  p=0.28 | | 10.8  8.34 - 15.9 | | 12.8  9.65 - 16.4 | | | 15.9  8.05 - 29.0 | | | χ^2^_(2,38)_=7.12,  **p=0.03** | | |
| PC aa C32:1 | 8.05  4.14 - 11.4 | 7.03  4.49 - 18.0 | 7.70  5.84 - 10.3 | χ^2^_(2,40)_=0.05,  p=0.97 | | 4.58*  2.87 - 6.29 | | 4.50  2.80 - 14.1 | | | 5.23  2.84 - 14.4 | | | χ^2^_(2,39)_=1.53,  p=0.47 | | |
| PC aa C32:2 | 1.20  0.67 - 1.37 | 1.12  0.66 - 1.96 | 1.17  0.65 - 1.56 | χ^2^_(2,40)_=0.17,  p=0.92 | | 0.74*  0.44 - 0.82 | | 0.58  0.38 - 1.12 | | | 0.77  0.44 - 1.01 | | | χ^2^_(2,39)_=5.18,  p=0.08 | | |
| PC aa C32:3 | 0.12  0.070 - 0.19 | 0.11  0.059 - 0.19 | 0.13  0.093 - 0.21 | χ^2^_(2,40)_=1.73,  p=0.42 | | 0.090  0.064 - 0.18 | | 0.088  0.048 - 0.17 | | | 0.12  0.065 - 0.16 | | | χ^2^_(2,39)_=4.89,  p=0.09 | | |
| PC aa C34:1 | 98.6  67.9 - 136 | 100  55.6 - 174 | 112  68.3 - 138 | χ^2^_(2,40)_=0.35, p=0.84 | | 105  70.1 - 157 | | 87.0  58.1 - 141 | | | 90.7  49.3 - 130 | | | χ^2^_(2,39)_=4.68,  p=0.10 | | |
| PC aa C34:2 | 229  172 - 299 | 245  132 - 373 | 217  143 - 269 | χ^2^_(2,40)_=1.58, p=0.46 | | 263  189 - 356 | | 202  126 - 343 | | | 235  123 - 294 | | | χ^2^_(2,39)_=6.95,  **p=0.03** | | |
| PC aa C34:3 | 10.9  6.75 - 14.5 | 11.2  5.76 - 18.0 | 10.8  5.93 - 13.8 | χ^2^_(2,40)_=0.09,  p=0.96 | | 5.80*  4.46 - 8.13 | | 4.88  3.08 - 8.90 | | | 6.21  3.74 - 8.15 | | | χ^2^_(2,39)_=5.42,  p=0.07 | | |
| PC aa C34:4 | 0.48  0.24 - 0.53 | 0.44  0.20 - 0.70 | 0.45  0.27 - 0.62 | χ^2^_(2,40)_=0.39,  p=0.82 | | 0.23*  0.17 - 0.31 | | 0.20  0.098 - 0.40 | | | 0.25  0.17 - 0.35 | | | χ^2^_(2,39)_=5.99,  p=0.05 | | |
| PC aa C36:0 | 1.86  1.25 - 2.61 | 1.76  0.80 - 3.10 | 1.74  0.99 - 2.93 | χ^2^_(2,40)_=0.25,  p=0.88 | | 1.78  1.41 - 2.49 | | 1.64  1.08 - 4.22 | | | 1.77  1.15 - 2.58 | | | χ^2^_(2,39)_=1.77,  p=0.41 | | |
| PC aa C36:1 | 16.6  8.34 - 21.5 | 15.9  8.98 - 28.0 | 19.2  10.8 - 23.1 | χ^2^_(2,40)_=1.46,  p=0.48 | | 15.5  10.8 - 21.9 | | 12.6  10.4 - 23.2 | | | 13.8  7.94 - 18.3 | | | χ^2^_(2,39)_=5.30,  p=0.07 | | |
| PC aa C36:2 | 125  86.9 - 147 | 122  66.5 - 185 | 126  75.6 - 162 | χ^2^_(2,40)_=0.04,  p=0.98 | | 175*  111 - 214 | | 114  77.8 - 211 | | | 143  74.6 - 185 | | | χ^2^_(2,39)_=8.57,  **p=0.01** | | |
| PC aa C36:3 | 58.6  35.1 - 73.6 | 52.0  33.4 - 94.9 | 61.6  35.8 - 76.5 | χ^2^_(2,40)_=0.07,  p=0.97 | | 58.6  39.1 - 78.1 | | 44.3  28.5 - 76.5 | | | 46.6  27.4 - 58.2 | | | χ^2^_(2,39)_=8.36,  **p=0.02** | | |
| PC aa C36:4 | 102  50.1 - 124 | 100  38.7 - 148 | 88.2  48.4 - 113 | χ^2^_(2,40)_=0.49,  p=0.79 | | 88.9  65.4 - 116 | | 65.6  43.1 - 125 | | | 85.2  52.9 - 106 | | | χ^2^_(2,39)_=7.98,  **p=0.02** | | |
| PC aa C36:5 | 5.29  3.61 - 6.97 | 4.80  3.27 - 8.87 | 5.46  3.41 - 6.53 | χ^2^_(2,40)_=0.03,  p=0.98 | | 3.64  2.31 - 5.08 | | 3.06  2.25 - 4.80 | | | 3.14  2.08 - 4.17 | | | χ^2^_(2,39)_=4.93,  p=0.09 | | |
| PC aa C36:6 | 0.31  0.18 - 0.41 | 0.29  0.15 - 0.48 | 0.33  0.23 - 0.38 | χ^2^_(2,40)_=0.15,  p=0.93 | | 0.20  0.13 - 0.32 | | 0.17  0.11 - 0.32 | | | 0.22  0.15 - 0.27 | | | χ^2^_(2,39)_=5.37,  p=0.07 | | |
| PC aa C38:0 | 1.05  0.53 - 1.53 | 1.20  0.43 - 2.03 | 1.17  0.57 - 1.86 | χ^2^_(2,40)_=0.20,  p=0.90 | | 0.89  0.68 - 1.34 | | 0.79  0.56 - 2.01 | | | 1.02  0.62 - 1.58 | | | χ^2^_(2,39)_=5.05,  p=0.08 | | |
| PC aa C38:1 | 0.94  0.42 - 1.19 | 0.70  0.34 - 2.00 | 0.77  0.33 - 2.11 | χ^2^_(2,40)_=1.02,  p=0.60 | | 0.65  0.54 - 1.11 | | 0.59  0.33 - 1.63 | | | 0.56  0.42 - 0.97 | | | χ^2^_(2,39)_=2.49,  p=0.29 | | |
| PC aa C38:3 | 15.9  9.06 - 20.2 | 15.1  8.70 - 25.1 | 18.5  10.2 - 23.6 | χ^2^_(2,40)_=1.22,  p=0.54 | | 17.5  11.0 - 26.7 | | 13.5  10.3 - 23.3 | | | 16.0  10.1 - 22.4 | | | χ^2^_(2,39)_=5.25,  p=0.07 | | |
| PC aa C38:4 | 53.0  24.2 - 62.4 | 52.5  17.9 - 75.3 | 53.7  28.0 - 68.5 | χ^2^_(2,40)_=0.44,  p=0.80 | | 44.6  35.9 - 74.0 | | 37.1  28.9 - 73.2 | | | 55.5  34.9 - 76.5 | | | χ^2^_(2,39)_=9.57,  **p=0.008** | | |
| PC aa C38:5 | 25.2  11.8 - 33.3 | 21.9  9.60 - 37.5 | 26.2  13.0 - 32.5 | χ^2^_(2,40)_=1.13,  p=0.57 | | 20.1  15.7 - 30.3 | | 16.9  13.3 - 27.4 | | | 21.2  14.1 - 26.7 | | | χ^2^_(2,39)_=4.00,  p=0.14 | | |
| PC aa C38:6 | 68.0  36.1 - 80.6 | 71.0  27.7 - 101 | 63.1  36.0 - 79.8 | χ^2^_(2,40)_=0.90,  p=0.64 | | 68.2  49.2 - 99.2 | | 51.9  39.4 - 91.8 | | | 71.8  40.0 - 87.5 | | | χ^2^_(2,39)_=6.65,  **p=0.04** | | |
| PC aa C40:2 | 0.44  0.19 - 0.56 | 0.41  0.19 - 0.65 | 0.41  0.20 - 0.61 | χ^2^_(2,40)_=1.10, p=0.58 | | 0.44  0.31 - 0.60 | | 0.33  0.24 - 0.73 | | | 0.39  0.28 - 0.66 | | | χ^2^_(2,39)_=4.45,  p=0.11 | | |
| PC aa C40:3 | 0.59  0.34 - 0.82 | 0.58  0.27 - 0.81 | 0.59  0.34 - 0.70 | χ^2^_(2,40)_=0.33,  p=0.85 | | 0.64  0.40 - 0.83 | | 0.51  0.23 - 0.84 | | | 0.54  0.37 - 0.74 | | | χ^2^_(2,39)_=2.73,  p=0.26 | | |
| PC aa C40:4 | 1.98  1.05 - 2.50 | 2.02  0.86 - 2.73 | 1.88  1.10 - 2.20 | χ^2^_(2,40)_=0.35,  p=0.84 | | 1.97  1.51 - 2.82 | | 1.63  1.34 - 3.20 | | | 2.20  1.21 - 2.63 | | | χ^2^_(2,39)_=6.13,  p=0.05 | | |
| PC aa C40:5 | 3.42  2.04 - 4.09 | 3.25  1.52 - 4.83 | 3.59  2.28 - 4.07 | χ^2^_(2,40)_=0.64,  p=0.73 | | 4.23  3.18 - 6.22 | | 3.71  2.80 - 5.53 | | | 4.41  3.01 - 5.79 | | | χ^2^_(2,39)_=4.02,  p=0.13 | | |
| PC aa C40:6 | 18.7  11.4 - 23.1 | 19.3  8.74 - 27.3 | 19.2  12.5 - 25.4 | χ^2^_(2,40)_=0.66,  p=0.73 | | 24.1  15.6 - 37.2 | | 19.4  14.7 - 30.5 | | | 26.0  14.1 - 34.0 | | | χ^2^_(2,39)_=6.23,  **p=0.04** | | |
| PC aa C42:0 | 0.16  0.065 - 0.22 | 0.14  0.080 - 0.21 | 0.14  0.10 - 0.22 | χ^2^_(2,40)_=0.50,  p=0.78 | | 0.17  0.12 - 0.20 | | 0.15  0.088 - 0.27 | | | 0.18  0.12 - 0.24 | | | χ^2^_(2,39)_=4.94,  p=0.09 | | |
| PC aa C42:1 | 0.15  0.084 - 0.19 | 0.13  0.049 - 0.24 | 0.15  0.077 - 0.24 | χ^2^_(2,40)_=0.30,  p=0.86 | | 0.15  0.13 - 0.19 | | 0.15  0.089 - 0.27 | | | 0.16  0.092 - 0.22 | | | χ^2^_(2,39)_=0.99,  p=0.61 | | |
| PC aa C42:2 | 0.24  0.14 - 0.30 | 0.24  0.13 - 0.35 | 0.22  0.15 - 0.28 | χ^2^_(2,40)_=1.18,  p=0.55 | | 0.23  0.18 - 0.35 | | 0.23  0.15 - 0.43 | | | 0.24  0.15 - 0.35 | | | χ^2^_(2,39)_=1.12,  p=0.57 | | |
| PC aa C42:4 | 0.20  0.095 - 0.28 | 0.12  0.078 - 0.24 | 0.18  0.13 - 0.21 | χ^2^_(2,40)_=1.31,  p=0.52 | | 0.22  0.16 - 0.27 | | 0.19  0.12 - 0.35 | | | 0.22  0.13 - 0.32 | | | χ^2^_(2,39)_=1.82,  p=0.40 | | |
| PC aa C42:5 | 0.30  0.13 - 0.39 | 0.27  0.12 - 0.41 | 0.27  0.16 - 0.29 | χ^2^_(2,40)_=0.80,  p=0.67 | | 0.29  0.18 - 0.38 | | 0.25  0.15 - 0.52 | | | 0.30  0.17 - 0.40 | | | χ^2^_(2,39)_=3.97,  p=0.14 | | |
| PC aa C42:6 | 0.73  0.45 - 0.96 | 0.76  0.43 - 0.97 | 0.74  0.55 - 0.79 | χ^2^_(2,40)_=0.70,  p=0.71 | | 0.78  0.60 - 1.07 | | 0.64  0.48 - 0.96 | | | 0.81  0.49 - 1.17 | | | χ^2^_(2,39)_=8.13,  **p=0.02** | | |
| ***Phosphatidylcholine acyl-alkyls*** | | | | | | | | | | | | | | | | |
| PC ae C30:0 | 0.21  0.000 - 0.28 | 0.20  0.000 - 0.27 | 0.20  0.000 - 0.39 | χ^2^_(2,40)_=0.80,  p=0.67 | | 0.18  0.000 - 0.28 | 0.17  0.000 - 0.36 | | | | | 0.20  0.000 - 0.25 | | | | χ^2^_(2,39)_=1.39,  p=0.50 |
| PC ae C30:1 | 0.27  0.11 - 0.49 | 0.24  0.075- 0.47 | 0.21  0.16 - 0.83 | χ^2^_(2,40)_=0.33,  p=0.85 | | 0.23  0.071 - 0.44 | 0.23  0.096 - 0.40 | | | | | 0.25  0.091 - 0.36 | | | | χ^2^_(2,39)_=0.13,  p=0.94 |
| PC ae C30:2 | 0.10  0.042 - 0.13 | 0.094  0.034 - 0.14 | 0.094  0.062 - 0.22 | χ^2^_(2,40)_=0.45,  p=0.80 | | 0.074  0.051 - 0.14 | 0.080  0.037 - 0.14 | | | | | 0.090  0.044 - 0.14 | | | | χ^2^_(2,39)_=0.80,  p=0.67 |
| PC ae C32:1 | 0.57  0.29 - 0.66 | 0.52  0.25 - 0.83 | 0.53  0.39 - 0.74 | χ^2^_(2,40)_=0.001,  p=1.00 | | 0.49  0.37 - 0.73 | 0.54  0.39 - 1.20 | | | | | 0.66  0.35 - 0.78 | | | | χ^2^_(2,39)_=4.53,  p=0.10 |
| PC ae C32:2 | 0.23  0.11 - 0.30 | 0.22  0.10 - 0.36 | 0.22  0.13 - 0.40 | χ^2^_(2,40)_=0.16,  p=0.92 | | 0.23  0.14 - 0.29 | 0.19  0.11 - 0.35 | | | | | 0.23  0.13 - 0.33 | | | | χ^2^_(2,39)_=2.81,  p=0.25 |
| PC ae C34:0 | 0.37  0.23 - 0.48 | 0.35  0.22 - 0.62 | 0.35  0.22 - 0.47 | χ^2^_(2,40)_=0.30,  p=0.86 | | 0.40  0.31 - 0.49 | 0.42  0.23 - 1.09 | | | | | 0.47  0.28 - 0.60 | | | | χ^2^_(2,39)_=3.92,  p=0.14 |
| PC ae C34:1 | 2.43  1.53 - 3.34 | 2.40  1.42 - 4.23 | 2.41  1.37 - 2.82 | χ^2^_(2,40)_=0.07,  p=0.97 | | 2.16  1.61 - 2.96 | 1.85  1.37 - 3.49 | | | | | 2.07  1.41 - 3.02 | | | | χ^2^_(2,39)_=1.96,  p=0.38 |
| PC ae C34:2 | 1.99  1.28 - 2.56 | 1.90  1.23 - 2.88 | 1.83  1.11 - 2.18 | χ^2^_(2,40)_=0.53, p=0.78 | | 2.24  1.53 - 3.10 | 1.80  1.09 - 3.51 | | | | | 2.03  1.32 - 3.18 | | | | χ^2^_(2,39)_=3.50,  p=0.17 |
| PC ae C34:3 | 0.53  0.39 - 0.73 | 0.57  0.40 - 0.87 | 0.59  0.37 - 0.70 | χ^2^_(2,40)_=0.12,  p=0.94 | | 0.71  0.48 - 0.10 | 0.55  0.37 - 1.12 | | | | | 0.73  0.38 - 1.02 | | | | χ^2^_(2,39)_=5.22,  p=0.07 |
| PC ae C36:0 | 0.51  0.22 - 0.87 | 0.41  0.17 - 1.22 | 0.55  0.24 - 1.57 | χ^2^_(2,40)_=2.19,  p=0.34 | | 0.41  0.27 - 0.51 | 0.34  0.24 - 0.85 | | | | | 0.41  0.26 - 0.54 | | | | χ^2^_(2,39)_=3.69,  p=0.16 |
| PC ae C36:1 | 2.47  1.29 - 3.73 | 2.21  1.21 - 5.31 | 2.62  1.29 - 5.44 | χ^2^_(2,40)_=0.54,  p=0.76 | | 2.45  1.76 - 3.62 | 2.10  1.42 - 4.37 | | | | | 2.23  1.47 - 2.97 | | | | χ^2^_(2,39)_=2.66,  p=0.27 |
| PC ae C36:2 | 5.32  3.36 - 6.99 | 4.87  3.00 - 7.59 | 5.02  3.01 - 6.20 | χ^2^_(2,40)_=0.67,  p=0.71 | | 7.96*  5.33 - 10.1 | 5.70  3.42 - 11.6 | | | | | 6.58  3.97 - 8.62 | | | | χ^2^_(2,39)_=5.25,  p=0.07 |
| PC ae C36:3 | 1.19  0.66 - 1.77 | 1.17  0.71 - 1.95 | 1.24  0.75 - 1.50 | χ^2^_(2,40)_=0.004,  p=1.00 | | 1.08  0.85 - 1.43 | 0.89  0.57 - 1.65 | | | | | 1.05  0.66 - 1.31 | | | | χ^2^_(2,39)_=5.11,  p=0.08 |
| PC ae C36:4 | 2.31  1.07 - 2.80 | 2.03  0.89 - 3.08 | 1.95  1.11 - 2.68 | χ^2^_(2,40)_=0.80,  p=0.67 | | 1.72  1.25 - 2.17 | 1.29  1.11 - 2.90 | | | | | 1.75  1.07 - 2.34 | | | | χ^2^_(2,39)_=5.49,  p=0.06 |
| PC ae C36:5 | 1.11  0.68 - 1.43 | 1.10  0.55 - 1.59 | 1.00  0.75 - 1.34 | χ^2^_(2,40)_=1.08,  p=0.58 | | 0.99  0.78 - 1.60 | 0.89  0.59 - 2.81 | | | | | 1.08  0.61 - 1.48 | | | | χ^2^_(2,39)_=4.39,  p=0.11 |
| PC ae C38:0 | 2.18  1.05 - 3.11 | 2.43  1.12 - 3.75 | 2.24  1.12 - 2.92 | χ^2^_(2,40)_=1.26,  p=0.53 | | 1.43  0.98 - 1.99 | 1.21  1.00 - 2.26 | | | | | 1.49  0.99 - 3.35 | | | | χ^2^_(2,39)_=4.19,  p=0.12 |
| PC ae C38:1 | 0.90  0.40 - 1.16 | 0.72  0.34 - 1.96 | 0.95  0.40 - 2.33 | χ^2^_(2,40)_=0.61,  p=0.74 | | 0.80  0.52 - 1.04 | 0.66  0.44 - 1.42 | | | | | 0.71  0.42 - 1.11 | | | | χ^2^_(2,39)_=2.44,  p=0.30 |
| PC ae C38:2 | 4.09  2.42 - 5.35 | 3.37  2.03 - 5.36 | 3.44  2.41 - 4.65 | χ^2^_(2,40)_=2.39,  p=0.30 | | 7.27*  4.46 - 8.52 | 4.77  3.11 - 10.1 | | | | | 5.17  3.29 - 7.56 | | | | χ^2^_(2,39)_=6.12,  p=0.05 |
| PC ae C38:3 | 1.33  0.74 - 1.79 | 1.25  0.63 - 2.03 | 1.36  0.83 - 1.78 | χ^2^_(2,40)_=0.08,  p=0.96 | | 1.62  1.17 - 2.10 | 1.17  0.86 - 2.51 | | | | | 1.36  0.95 - 1.99 | | | | χ^2^_(2,39)_=5.15,  p=0.08 |
| PC ae C38:4 | 2.49  1.18 - 2.94 | 2.33  0.90 - 3.27 | 2.30  1.20 - 2.82 | χ^2^_(2,40)_=0.27,  p=0.88 | | 2.55  1.84 - 3.24 | 1.93  1.350 - 2.78 | | | | | 2.69  1.80 - 3.71 | | | | χ^2^_(2,38)_=13.05,  **p=0.002** |
| PC ae C38:5 | 2.11  1.09 - 2.87 | 1.92  0.86 - 3.10 | 1.89  1.20 - 2.55 | χ^2^_(2,40)_=0.29,  p=0.86 | | 1.66  1.19 - 2.11 | 1.27  1.04 - 2.64 | | | | | 1.66  1.05 - 2.33 | | | | χ^2^_(2,39)_=5.11,  p=0.08 |
| PC ae C38:6 | 1.21  0.72 - 1.46 | 1.13  0.53 - 1.60 | 1.13  0.76 - 1.30 | χ^2^_(2,40)_=0.41,  p=0.82 | | 1.18  0.82 - 1.42 | 0.99  0.85 - 2.00 | | | | | 1.26  0.68 - 1.83 | | | | χ^2^_(2,39)_=5.58,  p=0.06 |
| PC ae C40:1 | 2.07  1.28 - 2.63 | 1.99  0.88 - 2.62 | 1.92  1.29 - 2.48 | χ^2^_(2,40)_=0.33,  p=0.85 | | 1.49  1.30 - 1.96 | 1.46  1.13 - 2.91 | | | | | 1.61  1.08 - 2.06 | | | | χ^2^_(2,39)_=4.37,  p=0.11 |
| PC ae C40:2 | 0.55  0.23 - 0.74 | 0.50  0.23 - 0.78 | 0.53  0.28 - 0.81 | χ^2^_(2,40)_=0.04,  p=0.98 | | 0.60  0.36 - 0.75 | 0.42  0.33 - 0.93 | | | | | 0.53  0.36 - 0.68 | | | | χ^2^_(2,39)_=5.01,  p=0.08 |
| PC ae C40:3 | 0.56  0.28 - 0.68 | 0.51  0.26 - 0.73 | 0.53  0.37 - 0.75 | χ^2^_(2,40)_=0.16,  p=0.92 | | 0.61  0.41 - 0.82 | 0.48  0.39 - 1.04 | | | | | 0.58  0.41 - 0.86 | | | | χ^2^_(2,39)_=4.92,  p=0.09 |
| PC ae C40:4 | 1.39  0.61 - 1.71 | 1.23  0.46 - 1.81 | 1.24  0.76 - 1.41 | χ^2^_(2,40)_=1.12,  p=0.57 | | 1.52  1.15 - 1.88 | 1.17  0.84 - 1.67 | | | | | 1.59  1.07 - 2.13 | | | | χ^2^_(2,38)_=10.2  **p=0.006** |
| PC ae C40:5 | 0.87  0.44 - 1.14 | 0.82  0.35 - 1.15 | 0.81  0.41 - 0.95 | χ^2^_(2,40)_=0.05,  p=0.97 | | 0.78  0.63 - 1.17 | 0.71  0.50 - 0.84 | | | | | 0.88  0.63 - 1.09 | | | | χ^2^_(2,38)_=9.37,  **p=0.009** |
| PC ae C40:6 | 1.32  0.68 - 1.64 | 1.21  0.58 - 1.66 | 1.18  0.80 - 1.66 | χ^2^_(2,40)_=0.33,  p=0.85 | | 1.61  1.14 - 2.11 | 1.20  0.95 - 1.80 | | | | | 1.78  1.08 - 2.53 | | | | χ^2^_(2,38)_=10.47,  **p=0.005** |
| PC ae C42:1 | 0.49  0.22 - 0.61 | 0.52  0.17 - 0.66 | 0.50  0.32 - 0.80 | χ^2^_(2,40)_=0.51,  p=0.77 | | 0.46  0.41 - 0.68 | 0.40  0.32 - 0.58 | | | | | 0.54  0.38 - 0.71 | | | | χ^2^_(2,38)_=10.0,  **p=0.007** |
| PC ae C42:2 | 0.40  0.18 - 0.50 | 0.42  0.16 - 0.61 | 0.37  0.29 - 0.56 | χ^2^_(2,40)_=0.57,  p=0.75 | | 0.32  0.24 - 0.48 | 0.30  0.23 - 0.52 | | | | | 0.36  0.24 - 0.46 | | | | χ^2^_(2,39)_=2.34,  p=0.31 |
| PC ae C42:3 | 0.83  0.38 - 1.11 | 0.77  0.27 - 0.98 | 0.73  0.35 - 1.02 | χ^2^_(2,40)_=1.49,  p=0.48 | | 0.55  0.46 - 0.89 | 0.48  0.33 - 0.77 | | | | | 0.64  0.42 - 0.80 | | | | χ^2^_(2,38)_=9.72,  **p=0.008** |
| PC ae C42:4 | 0.20  0.081 - 0.30 | 0.18  0.070 - 0.30 | 0.18  0.12 - 0.24 | χ^2^_(2,40)_=0.67,  p=0.72 | | 0.21  0.11 - 0.26 | 0.14  0.067 - 0.32 | | | | | 0.20  0.14 - 0.26 | | | | χ^2^_(2,39)_=3.64,  p=0.16 |
| PC ae C44:3 | 0.11  0.045 - 0.13 | 0.10  0.057 - 0.15 | 0.11  0.067 - 0.17 | χ^2^_(2,40)_=0.76,  p=0.68 | | 0.11  0.065 - 0.15 | 0.09  0.061 - 0.17 | | | | | 0.10  0.072 - 0.15 | | | | χ^2^_(2,39)_=2.79,  p=0.25 |
| PC ae C44:4 | 0.12  0.092 - 0.15 | 0.12  0.000 - 0.18 | 0.14  0.098 - 0.16 | χ^2^_(2,40)_=1.85,  p=0.40 | | 0.12  0.090 - 0.16 | 0.12  0.000 - 0.20 | | | | | 0.14  0.000 - 0.16 | | | | χ^2^_(2,39)_=1.37,  p=0.50 |
| PC ae C44:5 | 0.16  0.000 - 0.21 | 0.16  0.000 - 0.19 | 0.12  0.11 - 0.20 | χ^2^_(2,40)_=0.19,  p=0.91 | | 0.12  0.000 - 0.19 | 0.11  0.000 - 0.18 | | | | | 0.13  0.000 - 0.16 | | | | χ^2^_(2,39)_=1.79,  p=0.41 |
| PC ae C44:6 | 0.13  0.065 - 0.17 | 0.12  0.000 - 0.18 | 0.13  0.096 - 0.16 | χ^2^_(2,40)_=0.76,  p=0.68 | | 0.11  0.070 - 0.16 | 0.11  0.069 - 0.19 | | | | | 0.13  0.092 - 0.16 | | | | χ^2^_(2,39)_=3.35,  p=0.19 |
| **Sphingolipids** | | | | | | | | | | | | | | | | |
| SM(OH)C14:1 | 0.49  0.22 - 0.61 | 0.52  0.17 - 0.66 | 0.50  0.32 - 0.80 | χ^2^_(2,40)_=0.10,  p=0.95 | | 0.74*  0.44 - 1.14 | 0.61  0.38 - 0.84 | | | 0.66  0.31 - 1.11 | | | χ^2^_(2,39)_=3.53,  p=0.17 | | | |
| SM(OH)C22:1 | 0.40  0.18 - 0.50 | 0.42  0.16 - 0.61 | 0.37  0.29 - 0.56 | χ^2^_(2,40)_=0.26,  p=0.88 | | 1.44*  0.91 - 1.91 | 1.15  0.73 - 1.64 | | | 1.53  0.81 - 2.34 | | | χ^2^_(2,38)_=9.48,  **p=0.009** | | | |
| SM(OH)C22:2 | 0.83  0.38 - 1.11 | 0.77  0.27 - 0.98 | 0.73  0.35 - 1.02 | χ^2^_(2,40)_=0.72,  p=0.70 | | 1.00*  0.66 - 1.27 | 0.81  0.56 - 1.14 | | | 0.92  0.56 - 1.53 | | | χ^2^_(2,39)_=3.55,  p=0.17 | | | |
| SM(OH)C24:1 | 0.14  0.080 - 0.21 | 0.14  0.066 - 0.33 | 0.16  0.073 - 0.22 | χ^2^_(2,40)_=0.55,  p=0.76 | | 0.19  0.13 - 0.24 | 0.16  0.11 - 0.31 | | | 0.18  0.10 - 0.44 | | | χ^2^_(2,39)_=1.18,  p=0.55 | | | |
| SM C16:0 | 8.25  6.52 - 10.1 | 8.32  5.56 - 14.3 | 8.30  5.63 - 10.7 | χ^2^_(2,40)_=0.16,  p=0.92 | | 9.67  6.67 - 14.0 | 9.04  6.71 - 14.3 | | | 10.2  5.66 - 15.5 | | | χ^2^_(2,39)_=2.32,  p=0.31 | | | |
| SM C16:1 | 1.64  1.08 - 2.08 | 1.59  0.952 - 2.28 | 1.58  1.01 - 2.19 | χ^2^_(2,40)_=0.17,  p=0.92 | | 1.79  1.32 - 2.74 | 1.62  0.97 - 2.15 | | | 1.91  1.11 - 2.53 | | | χ^2^_(2,39)_=3.74,  p=0.15 | | | |
| SM C18:0 | 0.96  0.64 - 1.28 | 0.99  0.49 - 1.86 | 0.90  0.55 - 1.26 | χ^2^_(2,40)_=0.49,  p=0.78 | | 0.92  0.69 - 1.38 | 1.05  0.76 - 2.50 | | | 1.13  0.54 - 1.44 | | | χ^2^_(2,39)_=5.36,  p=0.07 | | | |
| SM C18:1 | 0.27  0.18 - 0.34 | 0.29  0.19 - 0.47 | 0.31  0.18 - 0.40 | χ^2^_(2,40)_=0.71,  p=0.70 | | 0.28  0.22 - 0.42 | 0.28  0.22 - 0.44 | | | 0.33  0.17 - 0.45 | | | χ^2^_(2,39)_=1.76,  p=0.42 | | | |
| SM C20:2 | 0.000  0.000 - 0.024 | 0.000  0.000 - 0.011 | 0.010  0.000 - 0.029 | χ^2^_(2,40)_=9.31,  **p=0.01** | | 0.013  0.000 - 0.034 | 0.006  0.000 - 0.020 | | | 0.014  0.000 - 0.035 | | | χ^2^_(2,39)_=2.62,pp=0.27 | | | |
| SM C24:0 | 3.44  2.53 - 4.77 | 3.61  2.04 - 5.35 | 3.49  2.48 - 4.05 | χ^2^_(2,40)_=0.23,  p=0.89 | | 4.42  2.96 - 6.02 | 3.70  3.10 - 4.95 | | | 4.46  2.59 - 7.02 | | | χ^2^_(2,38)_=10.41,  **p=0.006** | | | |
| SM C24:1 | 6.31  3.76 - 8.35 | 6.54  3.17 - 9.58 | 7.09  3.76 - 8.30 | χ^2^_(2,40)_=0.23,  p=0.89 | | 9.24*  6.66 - 13.8 | 7.59  5.78 - 12.4 | | | 10.3  5.56 - 15.5 | | | χ^2^_(2,39)_=4.69,  p=0.10 | | | |
| SM C26:0 | 0.023  0.000 - 0.049 | 0.028  0.000 - 0.060 | 0.022  0.000 - 0.067 | χ^2^_(2,40)_=0.16,  p=0.92 | | 0.021  0.000 - 0.055 | 0.027  0.000 - 0.10 | | | 0.033  0.000 - 0.085 | | | χ^2^_(2,39)_=3.24,  p=0.20 | | | |
| SM C26:1 | 0.016  0.000 - 0.080 | 0.025  0.000 - 0.066 | 0.040  0.000 - 0.063 | χ^2^_(2,40)_=3.62,  p=0.16 | | 0.036  0.000 - 0.069 | 0.030  0.000 - 0.14 | | | 0.033  0.000 - 0.066 | | | χ^2^_(2,39)_=0.04,  p=0.98 | | | |
| **Ratios of biomarkers** | | | | | | | | | | | | | | | | |
| (C2+C3)/C0 | 0.79  0.61 - 1.35 | 0.84  0.53 - 1.95 | 0.83  0.41 - 1.67 | χ^2^_(2,40)_=0.41,  p=0.82 | | 1.23  0.73 - 1.96 | 1.01  0.33 - 1.67 | | | 1.60  0.80 - 2.14 | | | χ^2^_(2,39)_=5.82,  p=0.06 | | | |
| (Leucine+  Glutamate)/  Glutamine | 1.43  0.60 - 4.49 | 1.66  0.35 - 6.01 | 1.46  0.29 - 5.07 | χ^2^_(2,40)_=1.07,  p=0.59 | | 0.79  0.30 - 2.50 | 1.54  0.43 - 4.58 | | | 1.10  0.50 - 4.77 | | | χ^2^_(2,39)_=3.92,  p=0.14 | | | |
| AAA | 218  153 - 259 | 227  170 - 447 | 228  158 - 433 | χ^2^_(2,40)_=0.63,  p=0.73 | | 184  152 - 246 | 166  144 - 308 | | | 193  142 - 440 | | | χ^2^_(2,39)_=3.47,  p=0.18 | | | |
| ADMA /  Arginine | 0.005  0.000 - 0.098 | 0.004  0.000 - 0.079 | 0.004  0.000 - 0.059 | χ^2^_(2,40)_=1.12,  p=0.57 | | 0.012  0.004 - 0.12 | 0.005  0.000 - 0.13 | | | 0.005  0.000 - 0.074 | | | χ^2^_(2,39)_=4.02,  p=0.13 | | | |
| Arginine /  (Arginine+  Ornithine) | 0.48  0.025 - 0.74 | 0.58  0.042 - 0.88 | 0.58  0.024 - 0.94 | χ^2^_(2,40)_=1.33,  p=0.51 | | 0.15  0.035 - 0.75 | 0.41  0.027 - 0.85 | | | 0.69  0.027 - 0.92 | | | χ^2^_(2,39)_=3.75,  p=0.15 | | | |
| BCAA | 373  308 - 467 | 447  309 - 985 | 441  298 - 939 | χ^2^_(2,40)_=7.84,  **p=0.02** | | 428  349 - 611 | 375  305 - 564 | | | 494  291 - 853 | | | χ^2^_(2,38)_=6.48,  **p=0.04** | | | |
| BCAA / AAA | 1.76  1.45 - 2.10 | 1.93  1.65 - 2.49 | 2.06  1.47 - 2.46 | χ^2^_(2,40)_=10.47,  **p=0.005** | | 2.35  2.11 - 2.65 | 2.22  1.83 - 2.47 | | | 2.36  1.94 - 3.08 | | | χ^2^_(2,39)_=4.07,  p=0.13 | | | |
| C14 / C16:1 | 0.80  0.60 - 0.97 | 0.79  0.61 - 1.00 | 0.81  0.64 - 1.06 | χ^2^_(2,40)_=0.12,  p=0.94 | | 1.12  0.92 - 1.37 | 1.02  0.61 - 1.41 | | | 1.10  0.84 - 1.51 | | | χ^2^_(2,39)_=3.20,  p=0.20 | | | |
| C16 / C16:1 | 2.87  2.19 - 3.14 | 2.79  1.86 - 3.41 | 2.59  1.97 - 3.42 | χ^2^_(2,40)_=0.15,  p=0.93 | | 3.44*  2.99 - 4.49 | 3.45  2.60 - 5.49 | | | 3.46  2.91 - 4.17 | | | χ^2^_(2,39)_=0.37,  p=0.83 | | | |
| C18 / C18:1 | 0.23  0.17 - 0.34 | 0.23  0.13 - 0.38 | 0.22  0.16 - 0.31 | χ^2^_(2,40)_=0.25,  p=0.88 | | 0.36*  0.33 - 0.39 | 0.37  0.23 - 0.57 | | | 0.33  0.29 - 0.53 | | | χ^2^_(2,39)_=1.09,  p=0.58 | | | |
| C2 / C0 | 0.77  0.59 - 1.31 | 0.83  0.51 - 1.92 | 0.81  0.39 - 1.63 | χ^2^_(2,40)_=1.21,  p=0.55 | | 1.20  0.70 - 1.93 | 0.99  0.27 - 1.65 | | | 1.57  0.76 - 2.10 | | | χ^2^_(2,39)_=5.77,  p=0.06 | | | |
| C3 / C0 | 0.023  0.017 – 0.040 | 0.023  0.015 – 0.042 | 0.022  0.016 – 0.040 | χ^2^_(2,40)_=0.13,  p=0.94 | | 0.028  0.018 – 0.037 | 0.028  0.021 – 0.038 | | | 0.038  0.029 – 0.045 | | | χ^2^_(2,38)_=7.00,  **p=0.03** | | | |
| C3 / C4 | 1.21  0.95 - 1.67 | 1.08  0.72 - 2.06 | 1.08  0.55 - 2.00 | χ^2^_(2,40)_=1.05,  p=0.59 | | 0.60*  0.35 – 1.07 | 0.75  0.52 - 5.57 | | | 0.71  0.42 – 1.11 | | | χ^2^_(2,39)_=3.96,  p=0.14 | | | |
| C4 / C0 | 0.023  0.017 - 0.040 | 0.023  0.015 - 0.042 | 0.022  0.016 - 0.037 | χ^2^_(2,40)_=0.10,  p=0.95 | | 0.049*  0.022 - 0.075 | 0.040  0.011 - 0.057 | | | 0.047  0.022 - 0.091 | | | χ^2^_(2,39)_=2.90,  p=0.24 | | | |
| C5 / C0 | 0.019  0.013 - 0.041 | 0.019  0.010 - 0.047 | 0.021  0.013 - 0.066 | χ^2^_(2,40)_=0.54,  p=0.76 | | 0.015*  0.010 - 0.024 | 0.014  0.011 - 0.020 | | | 0.019  0.009 - 0.036 | | | χ^2^_(2,39)_=7.53,  **p=0.02** | | | |
| C4 / C5 | 3.95  2.81 - 5.81 | 3.74  2.28 - 6.93 | 4.11  2.53 - 11.4 | χ^2^_(2,40)_=0.06,  p=0.97 | | 2.92  1.84 - 3.95 | 2.91  2.02 - 4.03 | | | 2.44  1.84 - 3.32 | | | χ^2^_(2,38)_=6.69,  **p=0.04** | | | |
| Citrulline /  Arginine | 0.77  0.34 - 11.6 | 0.41  0.14 - 10.7 | 0.54  0.31 - 15.2 | χ^2^_(2,40)_=3.76,  p=0.15 | | 2.27  0.62 - 17.7 | 1.22  0.15 - 17.3 | | | 0.71  0.090 - 9.28 | | | χ^2^_(2,39)_=3.08,  p=0.21 | | | |
| Citrulline /  Ornithine | 0.77  0.30 - 1.85 | 0.58  0.20 - 1.44 | 0.65  0.32 - 4.45 | χ^2^_(2,40)_=0.82,  p=0.66 | | 0.73  0.28 - 2.41 | 0.71  0.32 - 4.13 | | | 1.14  0.22 - 2.74 | | | χ^2^_(2,39)_=1.57,  p=0.46 | | | |
| CPT1 ratio | 0.009  0.005 - 0.014 | 0.009  0.005 - 0.018 | 0.009  0.004 - 0.014 | χ^2^_(2,40)_=1.23,  p=0.54 | | 0.011  0.007 - 0.020 | 0.009  0.005 - 0.020 | | | 0.014  0.008 - 0.027 | | | χ^2^_(2,39)_=11.13, **p=0.004** | | | |
| Essential AA | 1025  714 - 1178 | 1111  637 - 2130 | 1016  677 - 2059 | χ^2^_(2,40)_=1.49,  p=0.47 | | 960  815 - 1427 | 826  641 - 1850 | | | 1005  712 - 2229 | | | χ^2^_(2,39)_=2.41,  p=0.30 | | | |
| Glutamate /  Glutamine | 1.77  1.45 - 2.10 | 1.93  1.64 - 2.49 | 2.06  1.47 - 2.46 | χ^2^_(2,40)_=1.01,  p=0.60 | | 0.62  0.12 - 2.34 | 1.27  0.14 - 4.38 | | | 0.72  0.20 - 4.40 | | | χ^2^_(2,39)_=4.33,  p=0.12 | | | |
| Glycogenic  AA | 1210  620 - 1815 | 1307  456 - 1850 | 1088  580 - 2884 | χ^2^_(2,40)_=0.93,  p=0.63 | | 853  570 - 1372 | 888  488 - 2673 | | | 873  563 - 1395 | | | χ^2^_(2,39)_=0.02,  p=0.99 | | | |
| Glutamino-  lysis | 2.07  1.00 - 5.64 | 2.21  0.61 - 6.62 | 1.90  0.48 - 5.60 | χ^2^_(2,40)_=1.57,  p=0.46 | | 1.32  0.59 - 3.19 | 2.04  0.58 - 5.28 | | | 1.40  0.72 - 5.23 | | | χ^2^_(2,39)_=4.60,  p=0.10 | | | |
| Glycine /  Arginine | 5.76  3.19 - 136 | 4.29  1.22 - 119 | 9.37  2.29 - 134 | χ^2^_(2,40)_=2.49,  p=0.29 | | 14.4  3.16 - 89.2 | 8.98  1.40 - 121 | | | 5.20  1.40 - 81.6 | | | χ^2^_(2,39)_=1.10,  p=0.58 | | | |
| Glycine /  Glutamine | 0.59  0.35 - 0.81 | 0.59  0.31 - 0.86 | 0.55  0.30 - 1.65 | χ^2^_(2,40)_=1.49,  p=0.48 | | 0.42*  0.30 - 0.51 | 0.38  0.27 - 0.60 | | | 0.47  0.37 - 0.61 | | | χ^2^_(2,39)_=7.04,  **p=0.03** | | | |
| Glycine /  Histidine | 7.62  3.66 - 9.59 | 6.04  3.12 - 8.39 | 6.21  4.07 - 24.6 | χ^2^_(2,40)_=6.99,  **p=0.03** | | 4.25*  2.77 - 5.22 | 4.19  3.29 - 6.61 | | | 4.52  3.50 - 5.60 | | | χ^2^_(2,39)_=0.70,  p=0.71 | | | |
| Glycine /  PC ae C38:2 | 121  62.1 - 215 | 140  52.8 - 214 | 143  56.5 - 447 | χ^2^_(2,40)_=0.26,  p=0.88 | | 48.5  30.9 - 74.0 | 51.1  27.5 - 96.1 | | | 61.6  39.8 - 120 | | | χ^2^_(2,39)_=4.79,  p=0.09 | | | |
| Glycine /  Serine | 3.72  1.70 - 4.39 | 2.52  1.29 - 3.74 | 3.00  1.65 - 8.46 | χ^2^_(2,40)_=8.25,  **p=0.02** | | 2.06*  1.66 - 3.22 | 1.92  1.45 - 2.62 | | | 2.09  1.49 - 3.07 | | | χ^2^_(2,39)_=2.55,  p=0.28 | | | |
| Kynurenine /  Tryptophan | 0.018  0.011 - 0.025 | 0.018  0.010 - 0.026 | 0.016  0.010 - 0.037 | χ^2^_(2,40)_=1.11,p=0.57 | | 0.020  0.016 - 0.032 | 0.020  0.014 - 0.030 | | | 0.020  0.011 - 0.027 | | | χ^2^_(2,39)_=0.78,  p=0.68 | | | |
| lysoPC a C16:0/  lysoPC a C16:1 | 23.5  17.6 - 32.4 | 23.0  16.3 - 31.3 | 20.9  18.1 - 26.4 | χ^2^_(2,40)_=4.44,  p=0.11 | | 51.9  42.9 - 58.5 | 47.8  39.6 - 58.3 | | | 49.8  37.6 - 58.5 | | | χ^2^_(2,39)_=2.99,  p=0.22 | | | |
| lysoPC a C20:4/ lysoPC a C20:3 | 3.74  2.85 - 4.47 | 3.49  2.66 - 5.29 | 3.52  2.81 - 4.86 | χ^2^_(2,40)_=0.86,  p=0.65 | | 3.29  3.07 - 4.06 | 3.30  2.76 - 4.07 | | | 4.09  3.39 - 5.71 | | | χ^2^_(2,39)_=15.07, **p=0.0005** | | | |
| Non essential AA | 3974  2159 - 6864 | 3910  1207 - 10481 | 3559  1712 - 10018 | χ^2^_(2,40)_=0.51,  p=0.78 | | 2636  1682 - 5204 | 2968  1400 - 11818 | | | 2606  1442 - 6864 | | | χ^2^_(2,39)_=0. 60,  p=0.74 | | | |
| Ornithine/  Arginine | 1.10  0.35 - 39.0 | 0.74  0.14 - 23.0 | 0.45  0.069 - 40.3 | χ^2^_(2,40)_=1.33,  p=0.51 | | 5.87  0.33 - 27.8 | 1.47  0.18 - 35.5 | | | 0.45  0.080 - 34.9 | | | χ^2^_(2,39)_=3.78,  p=0.15 | | | |
| Ornithine/  Serine | 0.51  0.27 - 1.10 | 0.46  0.15 - 1.04 | 0.42  0.050 - 1.21 | χ^2^_(2,40)_=0.73,  p=0.69 | | 0.58  0.17 - 1.30 | 0.35  0.080 - 0.95 | | | 0.22  0.10 - 0.79 | | | χ^2^_(2,39)_=6.75,  **p=0.03** | | | |
| PC aa C28:1/  PC aa C38:1 | 0.49  0.27 - 0.67 | 0.52  0.23 - 1.06 | 0.59  0.24 - 1.66 | χ^2^_(2,40)_=0.54,  p=0.76 | | 0.55  0.29 - 1.01 | 0.68  0.32 - 1.19 | | | 0.63  0.34 - 1.11 | | | χ^2^_(2,39)_=0.92,  p=0.63 | | | |
| PC aa C28:1/  PC aa C40:2 | 1.16  0.60 - 1.58 | 1.05  0.82 - 1.59 | 1.11  0.81 - 3.25 | χ^2^_(2,40)_=0.25,  p=0.89 | | 0.86  0.62 - 1.48 | 1.03  0.59 - 2.48 | | | 0.99  0.58 - 1.59 | | | χ^2^_(2,39)_=0.30,  p=0.86 | | | |
| PC aa C36:3/  PC aa C36:4 | 0.64  0.53 - 0.79 | 0.63  0.44 - 0.86 | 0.71  0.48 - 0.81 | χ^2^_(2,40)_=1.54,  p=0.46 | | 0.67  0.53 - 0.85 | 0.66  0.55 - 0.86 | | | 0.52  0.46 - 0.67 | | | χ^2^_(2,39)_=18.39, **p=0.0001** | | | |
| PC aa C40:3/  PC aa C42:5 | 2.13  1.70 - 2.53 | 2.11  1.89 - 2.47 | 2.19  1.75 - 2.68 | χ^2^_(2,40)_=0.11,  p=0.95 | | 2.14  1.66 - 2.71 | 1.95  1.37 - 2.89 | | | 1.87  1.54 - 2.32 | | | χ^2^_(2,39)_=3.64,  p=0.16 | | | |
| PC aa C40:5/  PC aa C42:5 | 11.7  9.86 - 15.2 | 12.0  10.3 - 13.7 | 13.5  10.1 - 18.6 | χ^2^_(2,40)_=5.05,  p=0.08 | | 15.6*  10.6 - 23.9 | 15.5  10.4 - 22.0 | | | 14.3  11.4 - 21.1 | | | χ^2^_(2,39)_=0.39,  p=0.83 | | | |
| Tyrosine/  Phenylalanine | 0.72  0.57 - 0.89 | 0.66  0.50 - 0.91 | 0.67  0.48 - 0.91 | χ^2^_(2,40)_=2.08,  p=0.35 | | 0.60  0.50 - 0.77 | 0.55  0.49 - 0.81 | | | 0.63  0.48 - 0.93 | | | χ^2^_(2,39)_=2.51,  p=0.29 | | | |
| 5-HT/  Tryptophan | 0.14  0.060 - 0.24 | 0.15  0.060 - 0.27 | 0.17  0.090 - 0.37 | χ^2^_(2,40)_=2.50,  p=0.29 | | 0.08  0.040 - 0.29 | 0.09  0.050 - 0.17 | | | 0.10  0.040 - 0.26 | | | χ^2^_(2,39)_=0.23,  p=0.89 | | | |
| Spermidine/  Putrescine | 10.1  0.000 - 21.7 | 11.2  5.35 - 18.1 | 10.5  6.70 - 25.6 | χ^2^_(2,40)_=0.21,  p=0.90 | | 15.6  9.83 - 22.1 | 18.5  7.14 - 32.9 | | | 17.6  8.73 - 27.2 | | | χ^2^_(2,39)_=1.84,  p=0.40 | | | |
| Spermine/  Spermidine | 0.38  0.30 - 0.65 | 0.44  0.32 - 1.11 | 0.41  0.18 - 0.70 | χ^2^_(2,40)_=2.02,  p=0.37 | | 0.52  0.30 - 0.73 | 0.63  0.38 - 0.79 | | | 0.54  0.39 - 0.71 | | | χ^2^_(2,39)_=4.83,  p=0.09 | | | |

## **Supplementary Table S2**

Metabolite levels (µmoles, median and range) and their ratios in 129Sv repeated AMPH subgroups. CPT1 (carnitine palmitoyltransferase 1) ratio [(C16 + C18) / carnitine]. Significant Z-values (p < 0.05 ) are marked in bold.

| **129Sv** | | | | | | | | | | | | | | |
| --- | --- | --- | --- | --- | --- | --- | --- | --- | --- | --- | --- | --- | --- | --- |
| Biomarkers | | | | Weak  (N=7) | Strong  (N=7) | | Mann-Whitney *U-*test | | | | | | | |
|  |  |  |  |  |  |  | Z-value | | | | | | p-value | |
| **Acylcarnitines** | | | | | | | | | | | | | | |
| C0 | | | | 32.8  17.8 - 41.0 | 21.6  14.1 - 50.0 | | 0.77 | | | | | | 0.44 | |
| C10 | | | | 0.000  0.000 - 0.17 | 0.000  0.000 - 0.20 | | -0.77 | | | | | | 0.44 | |
| C12 | | | | 0.10  0.000 - 0.13 | 0.12  0.096 - 0.16 | | -2.04 | | | | | | **0.04** | |
| C14 | | | | 0.10  0.069 - 0.12 | 0.11  0.091 - 0.16 | | -1.60 | | | | | | 0.11 | |
| C14:1 | | | | 0.058  0.040 - 0.069 | 0.069  0.059 - 0.089 | | -2.11 | | | | | | **0.04** | |
| C14:1-OH | | | | 0.013  0.000 - 0.018 | 0.016  0.013 - 0.025 | | -1.98 | | | | | | **0.05** | |
| C14:2 | | | | 0.017  0.015 - 0.020 | 0.019  0.014 - 0.024 | | -0.32 | | | | | | 0.75 | |
| C16 | | | | 0.30  0.23 - 0.34 | 0.36  0.29 - 0.41 | | -1.92 | | | | | | 0.06 | |
| C16-0H | | | | 0.023  0.020 - 0.031 | 0.028  0.022 - 0.041 | | -1.72 | | | | | | 0.08 | |
| C16:1 | | | | 0.085  0.067 - 0.11 | 0.10  0.083 - 0.14 | | -2.04 | | | | | | **0.04** | |
| C16:1-OH | | | | 0.016  0.000 - 0.022 | 0.022  0.000 - 0.035 | | -1.92 | | | | | | 0.06 | |
| C18 | | | | 0.067  0.048 - 0.075 | 0.069  0.052 - 0.10 | | -0.26 | | | | | | 0.80 | |
| C18:1 | | | | 0.17  0.13 - 0.23 | 0.22  0.18 - 0.23 | | -1.92 | | | | | | 0.06 | |
| C18:2 | | | | 0.065  0.048 - 0.10 | 0.094  0.070 - 0.12 | | -1.66 | | | | | | 0.10 | |
| C2 | | | | 36.9  29.5 - 53.3 | 38.0  29.2 - 59.3 | | -0.13 | | | | | | 0.90 | |
| C3 | | | | 0.80  0.68 - 1.16 | 0.88  0.54 - 1.84 | | -0.13 | | | | | | 0.90 | |
| C4 | | | | 1.06  0.90 - 2.25 | 1.34  0.81 - 1.90 | | 0.00 | | | | | | 1.00 | |
| C3-DC (C4- OH) | | | | 0.37  0.25 - 0.67 | 0.38  0.19 - 0.85 | | -0.64 | | | | | | 0.52 | |
| C5 | | | | 0.48  0.34 - 0.68 | 0.53  0.33 - 1.03 | | -1.02 | | | | | | 0.31 | |
| C5-OH (C3-DC-M) | | | | 0.13  0.13 - 0.27 | 0.17  0.12 - 0.27 | | -0.45 | | | | | | 0.65 | |
| C6 (C4:1-DC) | | | | 0.063  0.041 - 0.072 | 0.062  0.046 - 0.076 | | -0.38 | | | | | | 0.70 | |
| C5-DC (C6-OH) | | | | 0.033  0.000 - 0.050 | 0.039  0.000 - 0.073 | | -0.64 | | | | | | 0.52 | |
| C7-DC | | | | 0.000  0.000 - 0.042 | 0.000  0.000 - 0.072 | | 0.00 | | | | | | 1.00 | |
| C8 | | | | 0.000  0.000 - 0.13 | 0.000  0.000 - 0.13 | | -0.38 | | | | | | 0.70 | |
| **Monosaccharides** | | | | | | | | | | | | | | |
| Hexoses | | | | 5551  2764 - 5937 | 3792  3284 - 4787 | | 2.04 | | | | | | **0.04** | |
| **Amino acids** | | | | | | | | | | | | | | |
| Alanine | | | | 447  265 - 552 | 354  221 - 643 | | | 0.64 | | | | | | 0.52 |
| Arginine | | | | 69.5  4.68 - 414 | 48.3  3.50 - 128 | | | 0.57 | | | | | | 0.57 |
| Asparagine | | | | 32.9  15.8 - 62.7 | 18.6  13.3 - 46.6 | | | 1.28 | | | | | | 0.20 |
| Aspartate | | | | 51.1  29.9 - 72.9 | 45.8  22.8 - 218 | | | 0.38 | | | | | | 0.70 |
| Citrulline | | | | 35.0  32.5 - 73.2 | 32.7  27.5 - 52.8 | | | 1.34 | | | | | | 0.18 |
| Glutamine | | | | 676  584 - 979 | 720  471 - 928 | | | 0.13 | | | | | | 0.90 |
| Glutamate | | | | 664  235 - 796 | 487  95.8 - 4080 | | | 0.26 | | | | | | 0.80 |
| Glycine | | | | 359  279 - 581 | 283  212 - 435 | | | 1.41 | | | | | | 0.16 |
| Histidine | | | | 68.5  65.4 - 130 | 68.2  59.2 - 95.3 | | | 0.64 | | | | | | 0.52 |
| Isoleucine | | | | 128  69.4 - 203 | 114  77.4 - 176 | | | 0.26 | | | | | | 0.80 |
| Leucine | | | | 169  107 - 413 | 179  129 - 350 | | | 0.13 | | | | | | 0.90 |
| Lysine | | | | 213  148 - 721 | 194  105 - 305 | | | 0.89 | | | | | | 0.37 |
| Methionine | | | | 36.2  29.4 - 161 | 37.4  25.8 - 54.7 | | | 0.00 | | | | | | 1.00 |
| Ornithine | | | | 35.2  26.4 - 57.2 | 22.5  11.3 - 152 | | | 0.64 | | | | | | 0.52 |
| Phenylalanine | | | | 78.6  56.8 - 206 | 71.3  56.8 - 141 | | | 0.32 | | | | | | 0.75 |
| Proline | | | | 66.5  51.1 - 210 | 65.7  47.3 - 106 | | | 0.00 | | | | | | 1.00 |
| Serine | | | | 169  117 - 262 | 147  92.0 - 211 | | | 0.70 | | | | | | 0.48 |
| Threonine | | | | 122  105 - 189 | 104  74.7 - 160 | | | 1.28 | | | | | | 0.20 |
| Tryptophan | | | | 67.1  51.5 - 98.8 | 58.6  51.6 - 77.3 | | | 0.89 | | | | | | 0.37 |
| Tyrosine | | | | 43.1  33.4 - 135 | 54.0  43.6 - 89.4 | | | 1.02 | | | | | | 0.31 |
| Valine | | | | 214  115 - 265 | 181  131 - 275 | | | 0.32 | | | | | | 0.75 |
| **Biogenic amines** | | | | | | | | | | | | | | |
| Ac-Orn | | | | 7.33  5.00 - 13.7 | 9.32  6.51 - 13.3 | | | | 1.28 | | | | | 0.20 |
| ADMA | | | | 0.27  0.20 - 0.37 | 0.22  0.000 - 0.52 | | | | 0.77 | | | | | 0.44 |
| alpha-AAA | | | | 0.000  0.000 - 6.43 | 0.000  0.000 - 0.000 | | | | 1.28 | | | | | 0.20 |
| Carnosine | | | | 2.99  1.29 - 7.52 | 2.93  1.46 - 5.83 | | | | 0.38 | | | | | 0.70 |
| Creatinine | | | | 11.4  7.29 - 18.2 | 12.0  6.11 - 17.3 | | | | 0.64 | | | | | 0.52 |
| Histamine | | | | 3.96  1.75 - 6.66 | 3.49  1.69 - 5.69 | | | | 0.13 | | | | | 0.90 |
| Kynurenine | | | | 1.19  1.05 - 1.45 | 1.27  1.10 - 1.40 | | | | 0.26 | | | | | 0.80 |
| Met-SO | | | | 0.000  0.000 - 0.000 | 0.000  0.000 - 0.000 | | | | 0.06 | | | | | 0.95 |
| PEA | | | | 0.072  0.069 - 0.079 | 0.073  0.069 - 0.11 | | | | 0.26 | | | | | 0.80 |
| Putrescine | | | | 0.69  0.46 - 1.11 | 0.72  0.37 - 1.45 | | | | 0.00 | | | | | 1.00 |
| Taurine | | | | 845  734 - 942 | 882  585 - 1020 | | | | 0.38 | | | | | 0.70 |
| 5-HT | | | | 7.65  2.55 - 14.9 | 7.06  2.96 - 14.6 | | | | 0.13 | | | | | 0.90 |
| Spermidine | | | | 12.0  7.57 - 22.2 | 13.3  4.23 - 32.6 | | | | 0.00 | | | | | 1.00 |
| Spermine | | | | 5.91  4.25 - 11.9 | 7.58  2.37 - 17.0 | | | | 0.00 | | | | | 1.00 |
| t4-OH-Pro | | | | 11.2  7.6 - 14.5 | 11.1  5.24 - 24.5 | | | | 0.00 | | | | | 1.00 |
| **Glycerophospholipids** | | | | | | | | | | | | | | |
| ***Lysophosphatidylcholine acyls*** | | | | | | | | | | | | | | |
| lysoPC a C16:0 | | 223  121 - 325 | | | 271  130 - 333 | | | | 0.70 | | 0.48 | | | |
| lysoPC a C16:1 | | 4.60  2.83 - 6.84 | | | 4.74  3.45 - 6.91 | | | | 0.00 | | 1.00 | | | |
| lysoPC a C17:0 | | 4.37  2.07 - 5.91 | | | 4.84  2.69 - 5.59 | | | | 0.51 | | 0.61 | | | |
| lysoPC a C18:0 | | 93.2  42.5 - 118 | | | 109  51 - 144 | | | | 0.83 | | 0.41 | | | |
| lysoPC a C18:1 | | 49.2  35.3 - 66.0 | | | 46.4  31.3 - 58.8 | | | | 0.51 | | 0.61 | | | |
| lysoPC a C18:2 | | 106  79.3 - 118 | | | 108  56.3 - 122 | | | | 0.51 | | 0.61 | | | |
| lysoPC a C20:3 | | 6.92  4.95 - 8.09 | | | 6.48  3.97 - 8.57 | | | | 0.26 | | 0.80 | | | |
| lysoPC a C20:4 | | 27.4  20.5 - 30.9 | | | 30.3  16.4 - 34.7 | | | | 0.70 | | 0.48 | | | |
| lysoPC a C24:0 | | 1.02  0.901 - 1.21 | | | 1.09  0.711 - 1.37 | | | | 0.00 | | 1.00 | | | |
| lysoPC a C26:0 | | 0.90  0.55 - 1.13 | | | 0.91  0.75 - 1.34 | | | | 0.26 | | 0.80 | | | |
| lysoPC a C26:1 | | 0.34  0.25 - 0.55 | | | 0.40  0.27 - 0.60 | | | | 0.00 | | 1.00 | | | |
| lysoPC a C28:0 | | 0.51  0.40 - 0.66 | | | 0.54  0.40 - 0.94 | | | | 0.38 | | 0.70 | | | |
| lysoPC a C28:1 | | 0.33  0.26 - 0.58 | | | 0.37  0.23 - 0.75 | | | | 0.00 | | 1.00 | | | |
| ***Phosphatidylcholine diacyls*** | | | | | | | | | | | | | | |
| PC aa C24:0 | | | 0.36  0.21 - 0.53 | | 0.38  0.24 - 0.58 | | | | 0.77 | | | 0.44 | | |
| PC aa C26:0 | | | 1.43  0.000 - 2.01 | | 1.69  0.000 - 2.02 | | | | 0.32 | | | 0.75 | | |
| PC aa C28:1 | | | 0.35  0.26 - 0.57 | | 0.50  0.25 - 0.57 | | | | 0.32 | | | 0.75 | | |
| PC aa C30:0 | | | 0.95  0.71 - 1.80 | | 1.47  0.79 - 2.74 | | | | 1.28 | | | 0.20 | | |
| PC aa C30:2 | | | 0.040  0.014 - 0.13 | | 0.060  0.000 - 0.15 | | | | 0.00 | | | 1.00 | | |
| PC aa C32:0 | | | 14.1  8.05 - 22.5 | | 18.7  9.35 - 29.0 | | | | 1.02 | | | 0.31 | | |
| PC aa C32:1 | | | 4.98  3.20 - 9.14 | | 5.48  2.84 - 14.4 | | | | 0.13 | | | 0.90 | | |
| PC aa C32:2 | | | 0.80  0.53 - 0.97 | | 0.61  0.44 - 1.01 | | | | 0.51 | | | 0.61 | | |
| PC aa C32:3 | | | 0.13  0.094 - 0.15 | | 0.11  0.065 - 0.16 | | | | 0.38 | | | 0.70 | | |
| PC aa C34:1 | | | 92.4  49.3 - 130 | | 77.6  54.4 - 115 | | | | 1.15 | | | 0.25 | | |
| PC aa C34:2 | | | 247  148 - 294 | | 213  123 - 281 | | | | 0.83 | | | 0.41 | | |
| PC aa C34:3 | | | 6.64  4.20 - 7.99 | | 5.19  3.74 - 8.15 | | | | 0.70 | | | 0.48 | | |
| PC aa C34:4 | | | 0.25  0.22 - 0.30 | | 0.24  0.17 - 0.35 | | | | 0.64 | | | 0.52 | | |
| PC aa C36:0 | | | 1.91  1.15 - 2.09 | | 1.71  1.25 - 2.58 | | | | 0.57 | | | 0.57 | | |
| PC aa C36:1 | | | 16.1  7.94 - 18.3 | | 12.5  9.67 - 16.8 | | | | 1.47 | | | 0.14 | | |
| PC aa C36:2 | | | 145  85.9 - 164 | | 128  74.6 - 185 | | | | 0.26 | | | 0.80 | | |
| PC aa C36:3 | | | 50.9  29.1 - 58.2 | | 42.6  27.4 - 47.9 | | | | 2.04 | | | **0.04** | | |
| PC aa C36:4 | | | 87.9  59.4 - 106 | | 83.1  52.9 - 99.5 | | | | 0.77 | | | 0.44 | | |
| PC aa C36:5 | | | 3.31  2.54 - 4.07 | | 2.79  2.08 - 4.17 | | | | 1.41 | | | 0.16 | | |
| PC aa C36:6 | | | 0.23  0.17 - 0.27 | | 0.21  0.15 - 0.24 | | | | 0.77 | | | 0.44 | | |
| PC aa C38:0 | | | 1.26  0.62 - 1.58 | | 0.90  0.66 - 1.33 | | | | 1.28 | | | 0.20 | | |
| PC aa C38:1 | | | 0.64  0.42 - 0.97 | | 0.55  0.52 - 0.75 | | | | 0.89 | | | 0.37 | | |
| PC aa C38:3 | | | 16.2  11.3 - 22.4 | | 16.0  10.1 - 18.6 | | | | 0.77 | | | 0.44 | | |
| PC aa C38:4 | | | 57.0  39.3 - 72.2 | | 54.0  34.9 - 76.5 | | | | 0.13 | | | 0.90 | | |
| PC aa C38:5 | | | 23.6  14.1 - 26.7 | | 20.2  15.3 - 21.9 | | | | 1.41 | | | 0.16 | | |
| PC aa C38:6 | | | 72.5  45.0 - 86.2 | | 69.2  40.0 - 87.5 | | | | 0.64 | | | 0.52 | | |
| PC aa C40:2 | | | 0.40  0.29 - 0.59 | | 0.38  0.28 - 0.66 | | | | 0.38 | | | 0.70 | | |
| PC aa C40:3 | | | 0.60  0.42 - 0.74 | | 0.51  0.37 - 0.67 | | | | 0.77 | | | 0.44 | | |
| PC aa C40:4 | | | 2.16  1.36 - 2.55 | | 2.23  1.21 - 2.63 | | | | 0.13 | | | 0.90 | | |
| PC aa C40:5 | | | 4.44  3.01 - 5.79 | | 4.37  3.01 - 4.80 | | | | 0.70 | | | 0.48 | | |
| PC aa C40:6 | | | 24.8  16.4 - 34.0 | | 28.8  14.1 - 33.7 | | | | 0.38 | | | 0.70 | | |
| PC aa C42:0 | | | 0.18  0.13 - 0.22 | | 0.18  0.12 - 0.24 | | | | 0.13 | | | 0.90 | | |
| PC aa C42:1 | | | 0.16  0.09 - 0.22 | | 0.16  0.11 - 0.18 | | | | 0.06 | | | 0.95 | | |
| PC aa C42:2 | | | 0.27  0.16 - 0.35 | | 0.22  0.15 - 0.29 | | | | 1.15 | | | 0.25 | | |
| PC aa C42:4 | | | 0.22  0.16 - 0.31 | | 0.20  0.13 - 0.32 | | | | 0.45 | | | 0.65 | | |
| PC aa C42:5 | | | 0.35  0.18 - 0.40 | | 0.30  0.17 - 0.39 | | | | 0.38 | | | 0.70 | | |
| PC aa C42:6 | | | 0.83  0.59 - 1.15 | | 0.73  0.49 - 1.17 | | | | 1.02 | | | 0.31 | | |
| ***Phosphatidylcholine acyl-alkyls*** | | | | | | | | | | | | | | |
| PC ae C30:0 | 0.20  0.16 - 0.21 | | | | 0.20  0.000 - 0.25 | | | | | 0.57 | | 0.57 | | |
| PC ae C30:1 | 0.23  0.17 - 0.36 | | | | 0.29  0.09 - 0.33 | | | | | 0.38 | | 0.70 | | |
| PC ae C30:2 | 0.085  0.044 - 0.14 | | | | 0.095  0.057 - 0.11 | | | | | 0.00 | | 1.00 | | |
| PC ae C32:1 | 0.66  0.45 - 0.78 | | | | 0.66  0.35 - 0.72 | | | | | 0.38 | | 0.70 | | |
| PC ae C32:2 | 0.23  0.17 - 0.33 | | | | 0.23  0.13 - 0.29 | | | | | 0.70 | | 0.48 | | |
| PC ae C34:0 | 0.43  0.28 - 0.60 | | | | 0.50  0.32 - 0.54 | | | | | 0.64 | | 0.52 | | |
| PC ae C34:1 | 2.25  1.41 - 3.02 | | | | 1.85  1.66 - 2.37 | | | | | 1.28 | | 0.20 | | |
| PC ae C34:2 | 2.08  1.52 - 3.18 | | | | 1.77  1.32 - 2.78 | | | | | 0.77 | | 0.44 | | |
| PC ae C34:3 | 0.76  0.43 - 0.91 | | | | 0.65  0.38 - 1.02 | | | | | 1.15 | | 0.25 | | |
| PC ae C36:0 | 0.43  0.26 - 0.54 | | | | 0.38  0.27 - 0.46 | | | | | 1.53 | | 0.13 | | |
| PC ae C36:1 | 2.48  1.47 - 2.97 | | | | 2.12  1.91 - 2.73 | | | | | 1.47 | | 0.14 | | |
| PC ae C36:2 | 7.10  4.03 - 8.62 | | | | 6.40  3.97 - 7.22 | | | | | 1.15 | | 0.25 | | |
| PC ae C36:3 | 1.11  0.66 - 1.31 | | | | 0.97  0.67 - 1.21 | | | | | 0.89 | | 0.37 | | |
| PC ae C36:4 | 1.68  1.17 - 2.34 | | | | 1.82  1.07 - 2.34 | | | | | 0.19 | | 0.85 | | |
| PC ae C36:5 | 1.14  0.61 - 1.48 | | | | 0.92  0.82 - 1.48 | | | | | 1.28 | | 0.20 | | |
| PC ae C38:0 | 1.58  0.99 - 3.35 | | | | 1.44  0.99 - 1.66 | | | | | 1.28 | | 0.20 | | |
| PC ae C38:1 | 0.76  0.51 - 1.11 | | | | 0.65  0.42 - 0.82 | | | | | 0.26 | | 0.80 | | |
| PC ae C38:2 | 5.24  3.36 - 7.56 | | | | 5.17  3.29 - 5.75 | | | | | 1.02 | | 0.31 | | |
| PC ae C38:3 | 1.49  0.95 - 1.99 | | | | 1.34  1.04 - 1.52 | | | | | 1.79 | | 0.07 | | |
| PC ae C38:4 | 2.72  1.80 - 3.71 | | | | 2.42  2.06 - 3.31 | | | | | 0.51 | | 0.61 | | |
| PC ae C38:5 | 1.60  1.05 - 2.33 | | | | 1.68  1.25 - 2.00 | | | | | 0.13 | | 0.90 | | |
| PC ae C38:6 | 1.28  0.68 - 1.83 | | | | 1.21  0.98 - 1.77 | | | | | 0.77 | | 0.44 | | |
| PC ae C40:1 | 1.76  1.08 - 2.06 | | | | 1.58  1.33 - 2.00 | | | | | 0.51 | | 0.61 | | |
| PC ae C40:2 | 0.56  0.36 - 0.68 | | | | 0.50  0.37 - 0.67 | | | | | 0.77 | | 0.44 | | |
| PC ae C40:3 | 0.59  0.41 - 0.86 | | | | 0.56  0.41 - 0.71 | | | | | 0.89 | | 0.37 | | |
| PC ae C40:4 | 1.71  1.07 - 2.13 | | | | 1.45  1.18 - 1.81 | | | | | 0.83 | | 0.41 | | |
| PC ae C40:5 | 0.91  0.63 - 1.09 | | | | 0.87  0.67 - 1.06 | | | | | 1.02 | | 0.31 | | |
| PC ae C40:6 | 1.80  1.08 - 2.53 | | | | 1.75  1.28 - 2.26 | | | | | 0.00 | | 1.00 | | |
| PC ae C42:1 | 0.54  0.41 - 0.71 | | | | 0.53  0.38 - 0.58 | | | | | 0.64 | | 0.52 | | |
| PC ae C42:2 | 0.36  0.24 - 0.46 | | | | 0.34  0.24 - 0.43 | | | | | 0.38 | | 0.70 | | |
| PC ae C42:3 | 0.67  0.46 - 0.80 | | | | 0.63  0.42 - 0.68 | | | | | 0.83 | | 0.41 | | |
| PC ae C42:4 | 0.21  0.14 - 0.26 | | | | 0.19  0.14 - 0.23 | | | | | 0.32 | | 0.75 | | |
| PC ae C44:3 | 0.10  0.072 - 0.15 | | | | 0.091  0.074 - 0.15 | | | | | 0.19 | | 0.85 | | |
| PC ae C44:4 | 0.14  0.11 - 0.16 | | | | 0.13  0.000 - 0.15 | | | | | 0.70 | | 0.48 | | |
| PC ae C44:5 | 0.14  0.11 - 0.16 | | | | 0.12  0.000 - 0.14 | | | | | 0.57 | | 0.57 | | |
| PC ae C44:6 | 0.13  0.092 - 0.16 | | | | 0.14  0.093 - 0.15 | | | | | 0.00 | | 1.00 | | |
| **Sphingolipids** | | | | | | | | | | | | | | |
| SM(OH)C14:1 | 0.66  0.31 - 1.11 | | | | 0.58  0.44 - 0.83 | | | | | 0.89 | | 0.37 | | |
| SM(OH)C22:1 | 1.45  0.81 - 2.34 | | | | 1.73  1.29 - 1.87 | | | | | 0.64 | | 0.52 | | |
| SM(OH)C22:2 | 0.86  0.56 - 1.53 | | | | 0.92  0.61 - 1.27 | | | | | 0.26 | | 0.80 | | |
| SM(OH)C24:1 | 0.18  0.10 - 0.44 | | | | 0.20  0.15 - 0.26 | | | | | 0.64 | | 0.52 | | |
| SM C16:0 | 11.4  5.66 - 15.5 | | | | 9.57  7.57 - 11.3 | | | | | 1.28 | | 0.20 | | |
| SM C16:1 | 2.02  1.11 - 2.53 | | | | 1.70  1.13 - 2.44 | | | | | 0.83 | | 0.41 | | |
| SM C18:0 | 1.24  0.54 - 1.43 | | | | 1.03  0.91 - 1.44 | | | | | 0.26 | | 0.80 | | |
| SM C18:1 | 0.32  0.17 - 0.45 | | | | 0.33  0.23 - 0.38 | | | | | 0.51 | | 0.61 | | |
| SM C20:2 | 0.014  0.000 - 0.035 | | | | 0.013  0.000 - 0.022 | | | | | 0.96 | | 0.34 | | |
| SM C24:0 | 4.51  2.59 - 5.81 | | | | 4.40  4.03 - 7.02 | | | | | 0.00 | | 1.00 | | |
| SM C24:1 | 9.37  5.56 - 15.5 | | | | 10.3  6.06 - 12.0 | | | | | 0.32 | | 0.75 | | |
| SM C26:0 | 0.029  0.015 - 0.085 | | | | 0.046  0.000 - 0.082 | | | | | 0.13 | | 0.90 | | |
| SM C26:1 | 0.029  0.000 - 0.066 | | | | 0.037  0.000 - 0.050 | | | | | 0.45 | | 0.65 | | |
| **Ratios of biomarkers** | | | | | | | | | | | | | | |
| (C2+C3)/C0 | | | 1.47  0.80 - 2.03 | | | 2.05  0.80 - 2.14 | | | 1.28 | | 0.20 | | | |
| (Leucine+Glutamate)/  Glutamine | | | 1.19  0.60 - 1.46 | | | 1.01  0.50 - 4.77 | | | 0.70 | | 0.48 | | | |
| AAA | | | 194  142 - 440 | | | 192  164 - 282 | | | 0.00 | | 1.00 | | | |
| ADMA/Arginine | | | 0.005  0.001 - 0.055 | | | 0.004  0.000 - 0.074 | | | 0.00 | | 1.00 | | | |
| Arginine/  (Arginine+Ornithine) | | | 0.64  0.076 - 0.92 | | | 0.76  0.028 - 0.80 | | | 0.38 | | 0.70 | | | |
| BCAA | | | 513  291 - 853 | | | 474  337 - 744 | | | 0.38 | | 0.70 | | | |
| BCAA/AAA | | | 2.34  1.94 - 2.66 | | | 2.47  2.05 - 3.08 | | | 0.77 | | 0.44 | | | |
| C14/C16:1 | | | 1.09  0.95 - 1.40 | | | 1.16  0.84 - 1.51 | | | 0.00 | | 1.00 | | | |
| C16/C16:1 | | | 3.52  2.91 - 4.17 | | | 3.02  2.91 - 3.78 | | | 1.09 | | 0.28 | | | |
| C18/C18:1 | | | 0.39  0.29 - 0.44 | | | 0.31  0.29 - 0.53 | | | 1.53 | | 0.13 | | | |
| C2/C0 | | | 1.43  0.78 - 2.00 | | | 2.02  0.76 - 2.10 | | | 1.28 | | 0.20 | | | |
| C3/C4 | | | 0.73  0.42 - 1.11 | | | 0.66  0.53 - 1.03 | | | 0.00 | | 1.00 | | | |
| C4/C0 | | | 0.038  0.022 - 0.091 | | | 0.058  0.029 - 0.085 | | | 0.83 | | 0.41 | | | |
| C4/C5 | | | 2.82  1.88 - 3.32 | | | 2.25  1.84 - 2.55 | | | 2.17 | | **0.03** | | | |
| Citrulline/Arginine | | | 0.78  0.085 - 6.99 | | | 0.69  0.40 - 9.28 | | | 0.00 | | 1.00 | | | |
| Citrulline/Ornithine | | | 1.02  0.57 - 2.02 | | | 1.22  0.22 - 2.74 | | | 0.51 | | 0.61 | | | |
| CPT-I ratio | | | 0.011  0.010 - 0.021 | | | 0.019  0.008 - 0.027 | | | 1.53 | | 0.13 | | | |
| Essential AA | | | 1006  712 - 2229 | | | 928  766 - 1407 | | | 0.38 | | 0.70 | | | |
| Fisher ratio | | | 2.34  1.94 - 2.66 | | | 2.48  2.06 - 3.08 | | | 0.70 | | 0.48 | | | |
| Glutamate/Glutamine | | | 0.72  0.35 - 1.22 | | | 0.68  0.20 - 4.40 | | | 0.64 | | 0.52 | | | |
| Glycogenic AA | | | 921  741 - 1395 | | | 827  563 - 1289 | | | 1.28 | | 0.20 | | | |
| Glutaminolysis | | | 1.57  0.84 - 2.06 | | | 1.26  0.72 - 5.23 | | | 0.77 | | 0.44 | | | |
| Glycine/Arginine | | | 5.39  1.40 - 76.7 | | | 4.68  3.29 - 81.6 | | | 0.51 | | 0.61 | | | |
| Glycine/Glutamine | | | 0.55  0.45 - 0.61 | | | 0.45  0.37 - 0.49 | | | 2.43 | | **0.02** | | | |
| Glycine/Histidine | | | 4.84  4.17 - 5.60 | | | 3.93  3.50 - 5.03 | | | 1.66 | | 0.10 | | | |
| Glycine/PC ae C38:2 | | | 72.4  47.5 - 111 | | | 60.8  39.8 - 120 | | | 0.64 | | 0.52 | | | |
| Glycine/Serine | | | 2.18  1.67 - 3.07 | | | 2.06  1.49 - 2.30 | | | 0.89 | | 0.37 | | | |
| Kynurenine/  Tryptophan | | | 0.019  0.011 - 0.025 | | | 0.022  0.016 - 0.027 | | | 1.28 | | 0.20 | | | |
| lysoPC a C16:0/  lysoPC a C16:1 | | | 48.3  42.9 - 54.7 | | | 51.8  37.6 - 58.5 | | | 1.02 | | 0.31 | | | |
| lysoPC a C20:4/  lysoPC a C20:3 | | | 4.05  3.39 - 4.47 | | | 4.13  3.44 - 5.71 | | | 0.51 | | 0.61 | | | |
| Non essential AA | | | 2754  2008 - 4083 | | | 2457  1442 - 6864 | | | 0.38 | | 0.70 | | | |
| Ornithine/Arginine | | | 0.57  0.08 - 12.2 | | | 0.32  0.25 - 34.9 | | | 0.38 | | 0.70 | | | |
| Ornithine/Serine | | | 0.24  0.13 - 0.49 | | | 0.19  0.10 - 0.79 | | | 0.00 | | 1.00 | | | |
| PC aa C28:1/  PC aa C38:1 | | | 0.60  0.34 - 1.11 | | | 0.76  0.47 - 0.93 | | | 0.89 | | 0.37 | | | |
| PC aa C28:1/  PC aa C40:2 | | | 0.90  0.58 - 1.59 | | | 1.05  0.68 - 1.30 | | | 0.38 | | 0.70 | | | |
| PC aa C36:3/  PC aa C36:4 | | | 0.53  0.49 - 0.67 | | | 0.52  0.46 - 0.61 | | | 1.15 | | 0.25 | | | |
| PC aa C40:3/  PC aa C42:5 | | | 1.87  1.72 - 2.32 | | | 1.74  1.54 - 2.20 | | | 0.96 | | 0.34 | | | |
| PC aa C40:5/  PC aa C42:5 | | | 16.1  11.4 - 21.1 | | | 13.4  12.1 - 18.0 | | | 0.32 | | 0.75 | | | |
| Tyrosine/  Phenylalanine | | | 0.60  0.48 - 0.74 | | | 0.75  0.58 - 0.93 | | | 1.66 | | 0.10 | | | |
| 5-HT/Tryptophan | | | 0.11  0.044 - 0.20 | | | 0.091  0.057 - 0.26 | | | 0.38 | | 0.70 | | | |
| Spermidine/Putrescine | | | 17.5  12.3 - 27.2 | | | 17.6  8.73 - 22.5 | | | 0.06 | | 0.95 | | | |
| Spermine/Spermidine | | | 0.50  0.39 - 0.71 | | | 0.55  0.52 - 0.58 | | | 1.15 | | 0.25 | | | |

## **Supplementary Table S3**

Spearman’s rank correlation coefficients between distance travelled (m), metabolites (µmoles) and their ratios in 129Sv repeated AMPH. Significant correlations (p < 0.05 ) are marked in bold.

|  | Distance traveled on day 11 | C12 | C14:1 | C14:1-OH | C16 | C16:1 | C18:1 | Hexoses | C4 / C5 | Glycine/ Glutamine | Glycine/ Histidine | Tyrosine/ Phenylalanine |
| --- | --- | --- | --- | --- | --- | --- | --- | --- | --- | --- | --- | --- |
| Distance traveled  on day 11 | 1.00 | 0.48 | 0.45 | 0.42 | **0.55** | **0.56** | 0.42 | -0.49 | **-0.62** | -0.50 | **-0.61** | **0.64** |
| C12 | 0.48 | 1.00 | **0.66** | **0.80** | **0.69** | **0.71** | **0.66** | **-0.60** | -0.43 | -0.34 | -0.38 | 0.49 |
| C14:1 | 0.45 | **0.66** | 1.00 | **0.58** | **0.86** | **0.89** | 0.40 | -0.28 | -0.41 | -0.27 | -0.39 | 0.07 |
| C14:1-OH | 0.42 | **0.80** | **0.58** | 1.00 | **0.65** | 0.52 | **0.68** | **-0.63** | -0.41 | -0.41 | -0.27 | 0.31 |
| C16 | **0.55** | **0.69** | **0.86** | **0.65** | 1.00 | **0.78** | 0.37 | -0.25 | **-0.60** | -0.17 | -0.33 | 0.18 |
| C16:1 | **0.56** | **0.71** | **0.89** | 0.52 | **0.78** | 1.00 | 0.53 | -0.47 | -0.24 | -0.26 | **-0.59** | 0.33 |
| C18:1 | 0.42 | **0.66** | 0.40 | **0.68** | 0.37 | 0.53 | 1.00 | **-0.96** | -0.04 | **-0.54** | -0.42 | 0.32 |
| Hexoses | -0.49 | **-0.60** | -0.28 | **-0.63** | -0.25 | -0.47 | **-0.96** | 1.00 | -0.03 | **0.61** | 0.50 | -0.42 |
| C4/C5 | **-0.62** | -0.43 | -0.41 | -0.41 | **-0.60** | -0.24 | -0.04 | -0.03 | 1.00 | 0.28 | 0.07 | -0.25 |
| Glycine/  Glutamine | -0.50 | -0.34 | -0.27 | -0.41 | -0.17 | -0.26 | **-0.54** | **0.61** | 0.28 | 1.00 | **0.57** | -0.26 |
| Glycine/  Histidine | **-0.61** | -0.38 | -0.39 | -0.27 | -0.33 | **-0.59** | -0.42 | 0.50 | 0.07 | **0.57** | 1.00 | **-0.62** |
| Tyrosine/  Phenyl-alanine | **0.64** | 0.49 | 0.07 | 0.31 | 0.18 | 0.33 | 0.32 | -0.42 | -0.25 | -0.26 | **-0.62** | 1.00 |

# 2. Quality Control

## Metabolite Quantitation

Biocrates AbsoluteIDQ™ p180 kit (Biocrates Life Sciences AG, Innsbruck, Austria) enables the measurement of 160 endogenous metabolites and 45 metabolite ratios using a combination of flow injection analysis and liquid chromatography tandem mass spectrometry technique. The assay allows simultaneous quantification of 188 metabolites, including 40 acylcarnitines, 21 amino acids, 21 biogenic amines, level of hexoses, 15 sphingolipids and 90 glycerophospholipids. The kit has been validated according to FDA guidelines and Biocrates company holds a ISO 9001:2008 certification of quality. Stable isotope standards are used for the quantification of biogenic amines and amino acids, using 7-point calibration curve. Acylcarnitines, phospho- and sphingolipids, and hexose were quantified by their relative intensity over the chosen isotopically labeled internal standard. In addition, 3 quality control standards are measured to ensure the normalization of signal intensities during inter-plate measurements. The metabolite concentrations were calculated linearly using a combination of Analyst (ABSciex, Framingham, USA) and MetIDQ (Biocrates Life Sciences AG, Innsbruck, Austria) software. This is done for amino acids and biogenic amines in the LC mode. Due to lack of isotopic standards for lipids and acylcarnitines in the FIA mode the results are classified as semi-quantitative.

## **Supplementary Table S4**

**Limits of Detection.** The absolute minimum limit of detection (LOD) for a metabolite is largely dependent on the sensitivity of the mass-spectrometer and the ionization of a metabolite. LODs, lower limit of quantification (LLOQ) and upper limit of quantification (ULOQ) for selected metabolites are given in table S5 (FIA) and table S6 (LC). Metabolites marked with an asterisk (*) are isotope corrected.

| **Analyte** | | **Quality Type (FIA)** | | **Evaluated Quantification** | | |
| --- | --- | --- | --- | --- | --- | --- |
| **MetIDQ Short Name** | **Biochemical Name** | **Valid** | **Semi** | **LOD (μM)** | **LLOQ (μM)** | **ULOQ (μM)** |
| Ac-Orn | Acetyl-ornithine |  | X | 0.15 | 1 | 40 |
| Alpha-AAA | Alpha-aminoadipic acid |  | X | 0.3 | 1 | 80 |
| Carnosine | Carnosine | X |  | 0.1 | 0.5 | 40 |
| Putrescine | Putrescine | X |  | 0.03 | 0.1 | 8 |
| Ile | Isoleucine | X |  | 1.5 | 5 | 400 |
| Leu | Leucine | X |  | 1.5 | 50 | 400 |
| Val | Valine | X |  | 0.5 | 10 | 800 |
| C0 | Carnitine | X |  | 4 | 5 | 120 |
| C2 | Acetylcarnitine | X |  | 0.15 | 0.4 | 35 |
| C4 | Bytyrylcarnitine* | X |  | 0.03 | 0.4 | 12 |
| C5 | Valerylcarnitine* | X |  | 0.04 | 0.4 | 12 |
| C16:1 | Hexadecenoylcarnitine* |  | X | 0.06 |  |  |
| C18:1 | Octadecenoylcarnitine* |  | X | 0.04 |  |  |
| LysoPC a C16:1 | Lysophosphatidylcholine acyl C16:1 |  | X | 0.07 |  |  |
| LysoPC a C17:0 | Lysophosphatidylcholine acyl C17:0 |  | X | 0.05 |  |  |
| PC aa C32:1 | Phosphatidylcholine diacyl C32:1* |  | X | 0.06 |  |  |
| PC aa C32:2 | Phosphatidylcholine diacyl C32:2* |  | X | 0.03 |  |  |
| PC aa C34:3 | Phosphatidylcholine diacyl C34:3* |  | X | 0.01 |  |  |
| PC aa C34:4 | Phosphatidylcholine diacyl C34:4 |  | X | 0.006 |  |  |
| PC aa C36:2 | Phosphatidylcholine diacyl C36:2* |  | X | 0.15 |  |  |
| PC aa C40:5 | Phosphatidylcholine diacyl C40:5* |  | X | 0.04 |  |  |
| PC aa C40:6 | Phosphatidylcholine diacyl C40:6* |  | X | 1.2 |  |  |
| PC ae C34:3 | Phosphatidylcholine acyl-alkyl C34:3 |  | X | 0.015 |  |  |
| PC ae C36:2 | Phosphatidylcholine acyl-alkyl C36:2* |  | X | 0.01 |  |  |
| PC ae C38:2 | Phosphatidylcholine acyl-alkyl C38:2* |  | X | 0.018 |  |  |
| PC ae C40:6 | Phosphatidylcholine acyl-alkyl C40:6* |  | X | 0.025 |  |  |
| SM (OH) C14:1 | Hydroxysphingomyeline C14:1* |  | X | 0.025 |  |  |
| SM (OH) C22:1 | Hydroxysphingomyeline C22:1* |  | X | 0.015 |  |  |
| SM (OH) C22:2 | Hydroxysphingomyeline C22:2* |  | X | 0.01 |  |  |
| SM (OH) C24:1 | Hydroxysphingomyeline C24:1* |  | X | 0.01 |  |  |
| SM C24:0 | Sphingomyeline C24:0* |  | X | 0.13 |  |  |
| SM C24:1 | Sphingomyeline C24:1* |  | X | 0.035 |  |  |
| H1 | Hexoses | X |  | 20 | 200 | 30000 |
